# Design, molecular characterization and therapeutic investigation of a novel CCR8 peptide antagonist that attenuates acute liver injury by inhibiting infiltration and activation of macrophages

**DOI:** 10.1016/j.apsb.2025.02.018

**Published:** 2025-02-21

**Authors:** Eline Geervliet, Sahil Arora, Dagmara Donohue, Carlos Antonio de Albuquerque Pinheiro, Leon W.M.M. Terstappen, Richard Schasfoort, Julieta Paez, Raj Kumar, Ruchi Bansal

**Affiliations:** a Personalized Diagnostics and Therapeutics, Department of Bioengineering Technologies, Technical Medical Centre, Faculty of Science and Technology, University of Twente, the Netherlands; b Institute of Molecular Pathobiochemistry, Experimental Gene Therapy and Clinical Chemistry, RWTH University Hospital Aachen, Germany; c Department of Pharmaceutical Sciences and Natural Products, School of Health Sciences, Central University of Punjab, India; d Developmental BioEngineering, Department of Bioengineering Technologies, Technical Medical Centre, Faculty of Science and Technology, University of Twente, the Netherlands

**Keywords:** CCL1‒CCR8 axis, Intrahepatic monocyte infiltration and differentiation, Liver inflammation, CCR8 antagonizing peptide, *In silico* molecular docking, *In vitro* chemotaxis, *In vivo* acute liver injury

## Abstract

During liver injury, intrahepatic macrophage compartment is augmented by circulating monocytes that infiltrate the liver driven by C–C motif chemokine ligand/C–C motif chemokine receptor (CCL/CCR) axis including CCL1‒CCR8 axis, thereby contributing to liver inflammation. Numerous small molecular receptor antagonists, including R243, have been developed for targeting CCR8; however, these agents face challenges in clinical translation, potentially attributed to their poor pharmacokinetic profiles, lack of target specificity, and potential adverse effects. In this study, we designed four CCR8 antagonizing peptides (AP8i-AP8iv) and performed molecular characterization *in silico* and therapeutic investigation *in vitro* and *in vivo*. Based on *in silico* docking, molecular dynamic simulation using homology build model and *in-vitro* (competitive) binding studies, AP8ii (YEWRFYHG) evidenced highly favorable and selective interactions at the CCR8-active site. AP8ii inhibited CCL1-driven chemotaxis and LPS/IFN*γ*-induced pro-inflammatory activation of monocytes-macrophages *in vitro*. In a CCl_4_-induced acute liver injury mouse model, AP8ii treatment decreased intrahepatic infiltration of circulating monocytes. Moreover, AP8ii reduced liver inflammation, as indicated by decreased F4/80, IL6 and iNOS expression, diminished ALT levels, and attenuated fibrosis, as indicated by reduced collagen-I expression. In conclusion, we report a novel CCR8-antagonizing peptide that inhibited CCL1-driven intrahepatic monocytes infiltration and differentiation into pro-inflammatory phenotype, consequently ameliorating liver inflammation and fibrogenesis in an acute liver injury mouse model.

## Graphical abstract

Liver injury driven by CCL1/CCR8-induced monocyte infiltration-differentiation and HSCs activation cause liver inflammation-fibrosis. This study presents a novel CCR8-antagonizing peptide (AP8) that inhibits liver injury by antagonizing CCL1/CCR8 axis.

**Figure:**
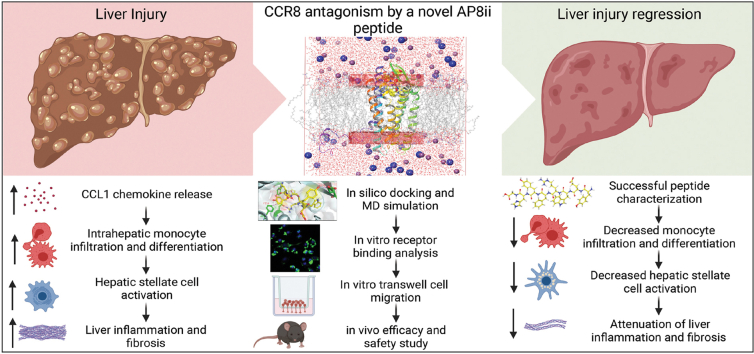

## 1 Introduction

Liver disease represents a burgeoning health concern with no adequate treatment^1^. Acute and/or chronic liver injury are initiated by the acute or chronic damage of hepatocytes followed by liver inflammation and fibrosis terminating in end-stage liver failure or hepatocellular carcinoma (primary liver cancer)^2^. Monocyte infiltration and their subsequent differentiation into monocyte-derived macrophages (MoMFs) contribute to liver inflammation and the commencement of fibrotic processes^3^, ^4^, ^5^, ^6^, ^7^, ^8^. After initial insult, hepatocytes, among other cells in the liver; secrete chemo-attractants such as C–C motif chemokine ligands (CCLs) to instigate intrahepatic infiltration of C–C motif chemokine receptors (CCRs) expressing circulating leukocytes^9^^,^^10^. Currently, 28 CCLs and 10 CCRs have been described that play a central role in physiological and pathological recruitment of immune cells^11^. CCL–CCR axis involved in monocyte infiltration and macrophage activation have gained focused attention in the recent years^12^, ^13^, ^14^, ^15^. While CCL2–CCR2/CCR5 is the most investigated axis in several pathologies, CCL1‒CCR8 axis remain rather underexplored.

The CCL–CCR system is largely promiscuous as many CCLs bind with several CCRs but CCR8 is unique as it only interacts with CCL1 (TCA-3 in mice, I-309 in humans) signifying the physiological importance of this interaction. CCL1‒CCR8 axis also play an important role in several pathologies including liver fibrosis^10^^,^^16^, pulmonary fibrosis^17^^,^^18^, asthma^19^^,^^20^, multiple sclerosis^21^^,^^22^, and cancer^23^^,^^24^. CCR8 is expressed on variety of cell types including circulating monocytes, endothelial cells and thymocytes^12^ where it can interact with MC148 (pox-viral derived) chemokine and vMIP-I (viral macrophage inflammatory protein-I from human herpes virus 8), besides CCL1, that are identified as CCR8 antagonists and agonists, respectively^25^^,^^26^. Upon activation, CCR8 triggers the induction of several adhesion molecules in the integrin family resulting in cell adhesion and intratissue infiltration^27^. Endothelial cells are hypothesized to respond to CCL1, contributing to the establishment of a gradient that facilitate the monocyte infiltration process^27^^,^^28^.

During liver injury, CCR8 is involved in the infiltration and differentiation of innate immune cells including monocytes, neutrophils, and NK cells, while it inhibits CD4^+^ T cell infiltration^16^. Studies using *Ccr8*^*−/−*^ mice evidenced attenuated liver injury as supported by reduced innate immune cells infiltration, increased Th1 *versus* Th2 polarization, decreased inflammation, fibrogenesis, as well as diminished alanine-aminotransferase (ALT) levels in carbon tetrachloride (CCl_4_)- and bile duct ligation (BDL)-induced liver injury mouse models^16^. Furthermore, using *Ccr8*^*−/−*^ mice and a small-molecule CCR8-antagonist R243, a crosstalk between CCR8 and toll-like receptor 4 (TLR4) signaling, and CCL1-induced CCR8-dependent cytokine secretion by macrophages upon LPS stimulation has been reported^27^^,^^29^. In addition, CCR8 involvement in the *trans*-differentiation of TGF*β*-treated mouse alveolar macrophages into myofibroblast-like cells is described indicating the direct role of CCR8 in (lung) fibrosis^30^.

Knowing the critical role of CCL1‒CCR8 axis in several pathologies, efforts have been made in the past years to understand the molecular interaction between CCL1 and CCR8, and to discover CCR8 antagonists^31^. Small-molecule antagonists that are described for CCR8 include oxazolidinones, SB-649701, by GSK^32^; naphthalene-sulfonamides, LMD-D, by Millennium^33^; diazaspiroundecane-based compound, AZ084, by AstraZeneca^34^; benzosulfamide-based compound IPG7236^35^; R243^29^ and LMD-A and LMD-B^36^. However, there are several challenges associated with the clinical application of small-molecule antagonists including lack of target specificity causing adverse effects, and poor bioavailability and pharmacokinetic profile requiring frequent or higher dosing^35^. Biologics including monoclonal antibodies, proteins and peptides are known for high specificity, improved pharmacokinetic properties, and improved therapeutic efficacy^37^, ^38^, ^39^. Therapeutic peptides in comparison to small molecule antagonists offer higher solubility, a greater safety profile, greater selectivity, less immunogenicity, and have low production costs. To date, a total of 80 therapeutic peptides drugs have been approved worldwide, and more than 170 peptides are in active clinical development with many more in preclinical studies^40^. In this study, we designed four CCR8 antagonizing peptides (AP8i-AP8iv), performed *in silico* modeling, docking and molecular dynamics simulation analysis, and *in vitro* (competitive) binding studies of AP8i-AP8iv with CCR8, and investigated the therapeutic efficacy of the most potent peptide (AP8ii) *in vitro* in monocytes and macrophages, and *in vivo* in CCl_4_-induced acute liver injury mouse model. To our best knowledge, this is the first study reporting a novel CCR8-antagonizing peptide with systematic molecular characterization and therapeutic investigation.

## 2 Materials and methods

### 2.1 Materials

Unlabeled and rhodamine B (RhB)-labeled peptides (AP8i-AP8iv, refer to Table 1), and biotinylated AP8ii peptide were synthesized and characterized by Lipopharm (Gdansk, Poland). Peptide characterization data is provided as Supporting Information-peptide characterization. Dulbecco's modified Eagle's medium (DMEM) (Life Technologies, Carlsbad, CA, USA); Roswell Park Memorial Institute (RPMI) 1640, with l-glutamine (Ca RPMI-A, Capricorn scientific, Ebsdorfergrund, Germany); Fetal Bovine Serum (FBS) (F7524, Sigma–Aldrich, St. Louis, MO, USA); Penicillin/Streptomycin, (pen/strep) (CA PS-B, Capricorn scientific); Nunc™ Lab-Tek™ Chamber Slide System (177445, Thermo Fisher Scientific, Wesel, Germany); Corning® Transwell® polycarbonate membrane cell culture inserts (CLS3421, Corning, New York, NY, USA); Lipopolysaccharides (LPS) from *Escherichia coli* O26:B6 (L2654, Sigma–Aldrich); interferon-gamma (IFN-*γ*) Mouse Recombinant (315-05-B, Tebu-bio, Le Perray-en-Yvelines, France); Human Recombinant I-309 (CCL1) (300-37-B, Tebu-bio); Phosphate Buffered Saline (PBS) tablets, (524650-1 EA, Millipore, Watford, UK); formaldehyde molecular biology reagent (F8775, Sigma–Aldrich); Fluoroshield^TM^ with 4,6-diamidino-2-fenylindool (DAPI) (F6057, Sigma–Aldrich); Trypan blue solution 0.4% (T8154, Sigma–Aldrich); Carbon tetrachloride (CCl_4_) (02671, Sigma–Aldrich); Olive oil (O1514, Sigma–Aldrich); hydroxypropyl-*β*-cyclodextrin (778966, Sigma–Aldrich); Kolliphor HS15 (70142-34-6, Sigma–Aldrich); R243 (HY-122219, MedChemExpress, South Brunswick Township, NJ, USA); Bovine Serum Albumin (BSA) (A7906-500G, Sigma–Aldrich); Epredia™ Cryomatrix™ embedding resin, Epredia 6769006 (12542716, Epredia, Runcorn, UK); 3-amino-9-ethylcarbazole (AEC) (A6926, Sigma–Aldrich); *N*,*N*-dimethyl fumarate (DMF) (227056, Sigma–Aldrich); phorbol 12-myristate 13-acetate (PMA, Thermo Fisher Scientific); hematoxylin solution according to Mayer (51275, Sigma–Aldrich); aqueous Mounting Medium Aquatex (1085620050, Sigma–Aldrich); SV Total RNA Isolation System (Z3105, Promega BeNeLux, Leiden, NL); iscript cDNA synthesis kit (1708891, BioRad, Lunteren, NL); SensiMixPlus SYBR & Fluorescein (QT615-20, GC biotech, Waddinxveen, NL).

**Table 1:** Gene-specific primer sets for quantitative RT-PCR.

Gene	Species	Forward primer	Reverse primer
*Gapdh*	Mouse	ACAGTCCATGCCATCACTGC	GATCCACGACGGACACATTG
*Ccr8*	Mouse	TGTTTGGGACTGCGATGTGT	TGATGGCATAGACAGCGTGG
*Adgre1*	Mouse	TGCATCTAGCAATGGACAGC	GCCTTCTGGATCCATTTGAA
*Inos*	Mouse	GGTGAAGGGACTGAGCTGTT	GCTACTCCGTGGAGTGAACAA
*Il6*	Mouse	TGATGCTGGTGACAACCACGGC	TAAGCCTCCGACTTGTGAAGTGGTA
*P*ro*collagen1α1*	Mouse	TGACTGGAAGAGCGGAGAGT	ATCCATCGGTCATGCTCTCT
*Timp1*	Mouse	ATCAGTGCCTGCAGCTTCTT	TGACGGCTCTGGTAGTCCTC
*Il10*	Mouse	TGGGTTGCCAAGCCTTATCG	TTCAGCTTCTCACCCAGGGA
*Arginase1*	Mouse	GTGAAGAACCCACGGTCTGT	CTGGTTGTCAGGGGAGTGTT

### 2.2 In situ analysis of CCR8 antagonizing peptides

#### 2.2.1 Molecular modeling studies

Molecular docking was conducted using the Schrodinger Maestro molecular docking suite (version 13.5) employing the Glide module. A CCR8 antagonist (R243) was initially drawn using ChemDraw Professional and then imported into Maestro in sdf format. Subsequently, all four designed peptides (AP8i-AP8iv) were sketched *via* the build option in the Maestro using their respective sequence. The Ligprep tool was used to prepare the ligand and peptides in 3D format. The OPLS4 force field was applied, and ionization states were set at pH 7.0 ± 2.0. Tautomers were generated for each neutralized or ionized molecule, with chiralities specified for up to one per ligand. The target protein sequence of CCR8 was retrieved from UniProt, and the template was selected based on homology using BLAST and HHpred. The Prime module within the Schrödinger Suite was utilized for modeling. Sequence alignment was achieved to match the target sequence with the selected templates, followed by 3D model generation and refinement using Prime's loop refinement algorithms. Side-chain conformations were predicted and optimized, and model quality was evaluated with Prime's validation tools to ensure structural integrity and accuracy. The final model underwent energy minimization and optimization to improve geometry and energetics.

The sequence of CCR8 was imported into Maestro, and a homology model was constructed *via* the Homology Modeling biologics approach. The protein was prepared using the Protein Preparation Workflow, which involved assigning bond orders, adding hydrogens, and introducing zero-order bonds to metal and disulfide bonds. Additionally, water molecules located beyond 5.00 Å were removed. Following this pre-processing, the protein in the workspace was analyzed and further refined through optimization, removal of excess water, and restrained minimization. After the R243, peptides and protein preparations, a receptor grid was generated, encompassing the CCR8 active site, and a receptor-grid file was created. Flexible R243-CCR8 and APs-CCR8 docking were performed using a prepared ligand (R243) and all four peptides at the active binding site of the prepared CCR8 protein, *via* a selected receptor grid file. The extra precision mode was followed, and the Root Mean Square Deviation (RMSD) was computed, resulting in the determination of the dock score and the binding energy affinity of the ligands^41^.

For mutation studies, the site-directed mutagenesis of the CCR8 protein, specifically the Tyr113 to Ala113 mutation, was performed using the Schrödinger Suite. The mutation was performed using the Prime module by selecting the Tyr113 residue and mutating it to Ala113, ensuring the side chain was properly modeled. The mutated structure was refined *via* Prime's loop refinement algorithms to adjust the local environment and optimize side-chain conformations. Further energy minimization was done to refine the overall structure.

For the receptor (CCR8) specificity, other CCR proteins such as CCR1 (PDB ID: 7VL8), CCR2 (PDB ID: 5T1A), CCR5 (PDB ID: 4MBS), CCR7 (PDB ID: 6QZH), and CCR9 (PDB ID: 5LWE) were prepared following the same protein preparation procedure as mentioned above. The molecular docking-modeling studies with the peptide were performed for the mutation studies and for the receptor specificity analysis as mentioned before.

#### 2.2.2 Molecular dynamics simulation studies

To investigate the behavior and stability of a potent inhibitor within the active site of the CCR8 binding site, molecular dynamics (MD) simulations were performed. The starting point for these simulations was the docking complex of peptide AP8ii and ligand R243 complex with CCR8. The MD simulations followed the standard Desmond protocol, involving a 100 ns simulation for equilibration and production runs.

The system was solvated using the TIP3P water model and neutralized by adding 0.15 mol/L Na^+^ and Cl^‒^ ions. A 10 Å water layer thickness was maintained. Prior to commencing the MD simulations, the complex underwent energy minimization with a maximum of 2000 steps. Temperature and pressure were carefully controlled at 300 K and 1.01325 bar, respectively, using an isothermal-isobaric (NPT) ensemble. Coulomb interactions were computed with a cutoff radius of 9 Å.

### 2.3 *In vitro* studies

#### 2.3.1 Cell lines

Murine RAW264.7 macrophages and human THP-1 monocytes, obtained from the American Type Culture Collection (ATCC, Manassas, VA, USA), were cultured in Roswell Park Memorial Institute (RPMI) 1640 medium supplemented with 10% FBS, 2 mmol/L l-glutamine and 1% penicillin–streptomycin, and were passaged twice a week as per established experimental protocols. All the cell lines used in this study were authenticated with short tandem repeat (STR) profiling and were tested regularly for the absence of mycoplasma contamination by PCR. RAW264.7 macrophages were used for (competitive) binding studies while both RAW264.7 macrophages and THP-1 monocytes were used for CCL1-induced Transwell migration assay.

#### 2.3.2 Bone marrow-derived macrophages

Bone marrow-derived macrophages (BMDMs) were freshly isolated from C57BL/6 mice as described previously elsewhere^42^. Briefly, femurs and tibias were flushed with DMEM with 10% FBS to collect bone marrow. Cells were then triturated 3–5 times through an 18-gauge needle and centrifuged at 1200 rpm for 5 min. After removing supernatant, RBCs were lysed with the lysis buffer and the remaining cells were washed in DMEM and plated at 1 × 10^6^ cells/mL in DMEM supplemented with 1% penicillin/streptomycin, 1% HEPES, 0.001% *β*-mercaptoethanol, 10% FBS, and 20% conditioned medium from mouse 3T3 fibroblasts [obtained from ATCC and cultured in cultured in (DMEM) medium supplemented with 2 mmol/L l-glutamine, 10% FBS and pen/strep]. The fibroblast conditioned medium is required to promote the differentiation of bone marrow cells into macrophages (7–10 days). Media was changed on Days 2, 4, and 6, and cells were re-plated on Day 7 for performing experiments.

#### 2.3.3 Competitive peptide binding studies

RAW264.7 (2 × 10^4^) macrophages were seeded on a Nunc™ Lab-Tek™ Chamber Slide in 300 μL of complete RPMI medium and incubated overnight under standard culture conditions (37 °C, 5% CO_2_). Next day, the cells were activated using 100 ng/mL LPS and 10 ng/mL interferon (IFN)-*γ* and incubated for 24 h. Cells were washed 3 times with 0.5% BSA in RPMI 0% FBS medium and incubated with 10 μmol/L of four different rhodamine B (RhB) conjugated antagonizing peptide for CCR8 (AP8i, AP8ii, AP8iii or AP8-iv) for 4 h at room temperature (RT). After 4 h, cells were washed 3 times with 0.5% BSA in RPMI 0% FBS medium, followed by 3 times PBS and fixed for 20 min at RT with 4% formaldehyde in PBS. After fixation, cells were washed 3 times in PBS, the Nunc™ Lab-Tek™ chambers including glue were carefully removed and cells were mounted in DAPI containing mounting medium. Images were captured at 10 ×, 20 × and 40 ×, and RhB intensity was analyzed using NIH ImageJ software and normalized to the number of nuclei using DAPI. Peptide with highest binding (AP8ii) was used in all subsequent experiments.

For competitive studies, the peptide binding protocol was used however competition with R243, CCL1 and anti-CCR8 antibody was performed. Two types of competitive studies were performed—co-incubation and pre-incubation where macrophages were either co-incubated with equimolar concentration of R243 (10 μmol/L), CCL1 (100 ng/mL) or anti-CCR8 antibody (5.0 μg/mL, Cat. No. MAB-8324, Novus biologicals, Zillow, USA) and AP8ii (10 μmol/L), or preincubated with R243 (10 μmol/L), CCL1 (100 ng/mL) or anti-CCR8 antibody (5.0 μg/mL) for 2 h followed by addition of AP8ii (10 μmol/L) for an additional 4 h. Finally, images were captured at 10 ×, 20 × and 40 ×, and RhB intensity was analyzed using ImageJ software and normalized to the number of nuclei using DAPI.

#### 2.3.4 Surface Plasmon Resonance (SPR) imaging for peptide-receptor interactions

Peptide interaction with CCR8 receptor, expressed on murine RAW264.7 macrophages and human THP-1 monocytes, was confirmed by the SPR imaging experiments. For these experiments, the recently developed CellVysion SPRi system (Vysens B.V. Hengelo, The Netherlands), integrated with a cuvette injection flow chamber for generating two parallel ligand density gradients on the sensor surface and for valve-free injection of samples, was used. “Back-and-forth” flow-based fluidics and for valve-free injection of samples enables unlimited interaction using only 70 μL of samples. The technology seamlessly measures affinity and avidity of biomolecular *i.e.*, ligand–receptor interaction. For this study, streptavidin-functionalized SPR sensors (Interfluidics B.V., Haaksbergen, The Netherlands) were incubated with biotinylated peptides to create gradient of biotinylated peptide by injecting 70 μL of AP8ii peptide (10 μmol/L). Peptides diffuse from the cuvette side to the end of the flow chamber creating a gradient where at the channel entrance a high density of ligands will be present gradually decreasing until there is no ligand density at the outlet of the flow chamber. The gradient ensures the availability of all ligand densities, from very high to low, up to zero. The zero-ligand density location is used as a reference signal to compensate for common mode signals such as bulk refractive index shifts, temperature effects, nonspecific binding to the hydrogel, etc. Following the peptide gradient, 1 × 10^6^ cells/mL RAW264.7 or THP-1 macrophages (differentiated with 50 ng/mL PMA) activated with 100 ng/mL LPS and 10 ng/mL IFN*γ* for 16 h were injected, sedimented, and washed under a fixed shear condition. For CCR8 blocking, species-specific anti-CCR8 antibodies (5.0 μg/mL) were added to the cells 2 h prior to the SPR experiments. The experiments were repeated at least three times by removing the cells using a buffer (PBS + 0.75 mol/L NaCl + 0.1% Tween 20) and by regenerating the sensor surface. White lines in the SPR images show the tipping point of cells binding to the sensor surface indicating peptide receptor interaction at different peptide density. Avidity of cell interaction is the aggregate strength of the number of binding sites in combination with the molecular affinity of the single biomolecular interaction. The ligand density at the tipping point is the characteristic parameter of avidity for cell–receptor interaction.

#### 2.3.5 Analytic HPLC characterization of the AP8ii peptide stability in human serum

Analytic HPLC measurements were conducted in an Agilent 1260 Infinity II Liquid Chromatography system, equipped with a Polaris C18 column and a UV–Vis detector. The samples were analyzed with a mobile phase of MilliQ (solvent A) and acetonitrile (solvent B), both containing 0.1% *v*/*v* of formic acid, using a 1 mL/min flow rate within a run of 22 min. The gradient ramp was from 1% solvent B up to 60% solvent B in 15 min, then from 60% solvent B to 99% solvent in 5 min, finally 2 min at 99% of solvent B. Each injection used 50 μL, out of the 150 μL in the vial. Detection was performed at *λ* = 280 nm. Integration of the signals (*e.g.*, peak area) in the chromatogram was performed using OpenLab CDS (data analysis software).

An adapted protocol based on^43^ was followed for analysis. In a vial, 475 μL of 1 mmol/L peptide solution was mixed with 50 μL of human serum (making 0.95 mmol/L peptide in 10 vol% serum). The sample was incubated at 37 °C for increasing time. At 0, 1, 2, 4 and 24 h, an aliquot of 125 μL was taken from the vial, quenched with 50 μL of 10% TFA solution in MilliQ and centrifuged for 1 min at 13,000 rpm. Then 150 μL of the supernatant was taken for analysis. Control samples containing only peptide (*e.g.*, without serum) and only human serum (*e.g.*, without peptide) were included. All measurements were conducted in duplicates. Data of peak absolute area were expressed as mean ± standard deviation (SD).

#### 2.3.6 Transwell migration assay

RAW264.7 macrophages: for the Transwell migration assay, RAW macrophages (1 × 10^5^) were seeded in the 24-well Transwell inserts with 5.0 μm polycarbonate membranes in 100 μL RPMI complete medium supplemented with 100 ng/mL LPS and 10 ng/mL IFN-*γ* with and without AP8ii or R243 (10 μmol/L). The lower well was filled with 600 μL RPMI complete medium containing 10 ng/mL CCL1 to induce Transwell migration and cells were incubated for 24 h. After 24 h, the inside of the Transwell were cleaned with a cotton swap to remove non-migrated cells. The migrated cells on the bottom side were washed 3 times with PBS and fixed for 20 min at RT with 4% formaldehyde in PBS. After fixation, cells were washed 3 times with PBS and permeabilized for 5 min in ice-cold methanol. Cells were washed again 3 times with PBS and membranes were cut out using a scalpel, put on a glass slide, mounted with DAPI containing mounting medium. Representative images were captured at 10 × under a microscope, and DAPI-stained nuclei were counted using ImageJ.

THP-1 monocytes: Transwell migration assay was performed as described above, with minor modifications. THP-1 (1 × 10^5^) monocytes seeded in the Transwell inserts were activated with 1 μg/mL LPS in the presence and absence of AP8ii or R243 (10 μmol/L). After 24 h, Transwell inserts were discarded and cells in the lower chamber were collected. Cells were centrifuged at 300×*g* for 10 min and supernatant was discarded to remove cell debris. The cell pellets were resuspended in 100 μL of medium, stained with trypan blue and living cells were counted using an automated cell counter.

Bone marrow-derived macrophages (BMDMs): Transwell migration assay was performed as described above, with minor modifications. BMDMs (1 × 10^5^) seeded in the Transwell inserts were activated with 100 ng/mL LPS and 10 ng/mL IFN-*γ* in the presence and absence of AP8ii or R243 (10 μmol/L). After 24 h, the inside of the Transwell were cleaned with a cotton swap to remove non-migrated cells. The migrated cells on the bottom side and in the lower chamber were washed 3 times with PBS and fixed for 20 min at RT with 4% formaldehyde in PBS. After fixation, cells were washed 3 times with PBS and permeabilized for 5 min in ice-cold methanol. Cells were washed again 3 times with PBS and membranes were cut out using a scalpel, put on a glass slide were mounted with DAPI containing mounting medium. The cells in the lower chamber were mounted with DAPI containing mounting medium. Representative images were captured at 10 × under a microscope, and DAPI-stained nuclei were counted using ImageJ.

#### 2.3.7 Cell viability assay

To assess the effects of AP8ii on the cell viability, cells were plated in 24-well plates, cultured overnight and incubated with AP8ii or R243 (10 μmol/L) with or without 100 ng/mL LPS and 10 ng/mL IFN*γ* (for 24 h, cell viability assays were performed using Alamar Blue reagent (Invitrogen, Carlsbad, CA, USA) as per manufacturer's instructions. The results are represented as% cell viability normalized to untreated control cells (at 100%). All measurements were performed in triplicates in three independent experiments.

#### 2.3.8 *In vitro* efficacy on RAW264.7 macrophages - gene expression analysis

RAW264.7 macrophages were plated (1 × 10^6^ cells/well) in 12 well plates and cultured overnight. The cells were then incubated with LPS (100 ng/mL) and IFN*γ* (10 ng/mL) or IL-4 (10 ng/mL) and IL-13 (10 ng/mL) for 24 h. To study the effect of AP8ii and R243; RAW macrophages were incubated with medium alone, 10 μmol/L of AP8ii (with and without 5 μg/mL anti-CCR8 antibodies) and R243 with and without LPS (100 ng/mL) and IFN*γ* (10 ng/mL) for 24 h. Cells were then lysed either with RNA lysis buffer for quantitative real-time PCR analysis. All the efficacy studies were performed at least 3 times independently.

### 2.4 *In vivo* studies

#### 2.4.1 CCl_4_-induced acute liver injury mouse model

All the animal experiments were carried out according to the ethical guidelines for the Care and Use of Laboratory Animals (Utrecht University, The Netherlands). CCl_4_ was prepared in olive oil (1:40 dilution, 63.76 mg/mL) and AP8ii (1 μmol/kg) and R243 (1 μmol/kg) were prepared in hydroxypropyl-*β*-cyclodextrin (10%) and Kolliphor HS15 (5%) containing MilliQ water (85%). Male C57BL/6 mice (12 weeks old) received a single intraperitoneal injection (IP) of olive oil (healthy *n* = 9) or 0.2 mL/kg (318.8 mg/kg) CCl_4_ (vehicle *n* = 9, AP8 *n* = 9 and R243 *n* = 9) on Day 1. On Days 2 and 3, the CCl_4_-treated mice received vehicle, 1 μmol/kg AP8 (1.157 mg/kg) or 1 μmol/kg R243 (0.35744 mg/kg) twice daily. On Day 4, the animals were euthanized, organs (liver, lungs, kidneys, spleen, and heart) were harvested, weighed, and liver tissues and blood were processed for further analyses. Total alanine aminotransferase (ALT) and aspartate transferase (AST) levels were determined in the plasma samples as per standard biochemical assays.

#### 2.4.2 Cell suspension and flow cytometry

Cell suspension were prepared from freshly collected liver tissues using the TissueGrinder (TG, Fast Forward Discoveries GmbH, Mannheim, Germany) as detailed earlier^44^. Prepared cell suspension was washed 3 times with 1 mL PBS containing 2% FBS, and the cells were fixed with 4% formaldehyde (prepared in PBS with 2% FBS) for 30 min at RT. Cells were washed 3 times, stained with rat anti-mouse fluorescent antibodies (CD45-APC, CD11b-PE and F4/80-PE/Cy7, 0.2 μg/100 μL, Biolegend) for 1 h, Hoechst 33,342 (10 μg/100 μL, Thermo Fisher) for 30 min, and analyzed within 7 days. Cells were identified using flow cytometry (BD FACS Aria II BD, Bioscience, San Jose, CA) and the data was analyzed using FlowJo v10.7.0 (Tree Star, Inc., Ashland, OR). For the gating strategy. Contour plots are used to depict the monocyte-macrophage populations (CD11b^++^F4/80^+^ as MoMFs and CD11b^+^F4/80^++^ as KCs) identified using flow cytometric analysis.

#### 2.4.3 Immunohistochemical staining

Liver tissues were harvested, embedded in Cryomatrix™ and fixated/snap-frozen in chilled 2-methylbutane on dry ice. Cryosections (6 μm) were cut using a Leica CM 1860 cryostat (Leica Microsystems, Nussloch, Germany). The sections were air-dried and fixed in acetone for 20 min at RT. Thereafter, sections were rehydrated in PBS and incubated with the primary antibodies: rabbit anti-mouse CCR8 (Cat. No. MAB-8324, Novus biologicals); rat anti-mouse F4/80 (Cat. No. MCA497, BioRad) or goat anti-mouse collagen-I (Cat. No. 1310-01, Southern Biotech, Birmingham, USA) at 4 °C overnight. Next day, sections were washed 3 times in PBS. Endogenous peroxidase activity was blocked by 0.3% H_2_O_2_ prepared in methanol for 30 min. Sections were washed 3 times in PBS and then incubated with horseradish peroxidase (HRP)-conjugated secondary antibody (Dako, Agilent, Abcoude, Netherlands) for 1 h at RT. Thereafter, sections were washed 3 times in PBS and incubated with HRP-conjugated tertiary antibody for 1 h at RT and again washed 3 times in PBS. 3-Amino-9-ethylcarbazole (AEC) solution was freshly prepared by combining 4.5 mL MilliQ, 500 μL 1 mol/L sodium acetate pH 5.5 and 250 μL AEC (1 tablet AEC dissolved in 2.5 mL dimethylformamide, DMF) followed by filtering using a 4.5 μm nylon filter and lastly 5.2 μL 30% H_2_O_2_ was added. Peroxidase activity was developed using AEC solution for 20 min at RT, and nuclei were counterstained with hematoxylin for 5 min. After 5 min of washing under tap water, sections were mounted with Aquatex mounting medium. Slides were digitized using a NanoZoomer 2.0 HT whole slide scanner (Hamamatsu, Shizuoka, Japan) and resulting digital images were visualized at 1680 × 1050-pixel resolution with NDP software and analyzed using ImageJ software.

#### 2.4.4 Co-immunofluorescent staining

Immunofluorescent staining was performed using rabbit anti-mouse CCR8 (Cat. No. MAB-8324, Novus biologicals) and rat anti-mouse F4/80 (Cat. No. MCA497, BioRad) or CCR8 and rat anti-mouse CD11b (BioLegend). Following overnight incubation with primary antibodies (1:100 dilution), liver sections were washed 3 times with PBS and incubated with goat anti-rat secondary antibody (Dako) for 1 h at room temperature. Following 3 times with PBS, liver sections were stained with Alexa Fluor (AF)488 or AF594-conjugated secondary antibodies (ThermoFisher). After 1 h, all sections were washed 3 times in PBS and mounted with DAPI mounting medium. Fluorescent images were made using a Nikon E400 microscope (Nikon). ImageJ was used to make the overlay images.

#### 2.4.5 Quantitative RT-PCR

Total RNA from cells and liver tissues was isolated using GenElute Total RNA Miniprep Kit (Sigma) and SV Total RNA Isolation System (Promega) respectively as per manufacturer's instructions. The RNA concentration was quantified with BioSpec-nano Spectrophotometer (Shimadzu Europa GmbH, Duisburg, Germany) and total RNA (1 μg) was reverse transcribed using the iScript cDNA Synthesis Kit. Real-time PCR was performed using 20 ng of cDNA, pre-tested mouse gene-specific primer sets (listed in Table 1) and 2 × SensiMix SYBR (GC biotech) according to the manufacturer's instructions. PCR was performed in CFX384 Touch Real-Time PCR Detection System (Bio-Rad). Finally, cycle threshold (Ct) values were normalized to reference gene *Gapdh* and fold changes in expression were calculated using the 2^−ΔΔCt^-method.

#### 2.4.6 Graphs and statistical analysis

All graphs were made, and statistical analysis was performed using GraphPad Prism version 10.0.1 (GraphPad Prism, La Jolla, CA, USA). The results are expressed as the mean ± standard error of the mean (SEM). Multiple comparisons between different groups were calculated using the one-way analysis of variance (ANOVA) with the Bonferroni *post hoc* test. Statistical differences between two groups were calculated using a two-tailed unpaired *t*-test. Differences were considered significant when ∗*P* < 0.05, ∗∗*P* < 0.01, ∗∗∗*P* < 0.001, or ∗∗∗∗*P* < 0.0001.

## 3 Results

### 3.1 CCR8 expression correlates with the migration and differentiation of hepatic macrophages

**Figure 1:**
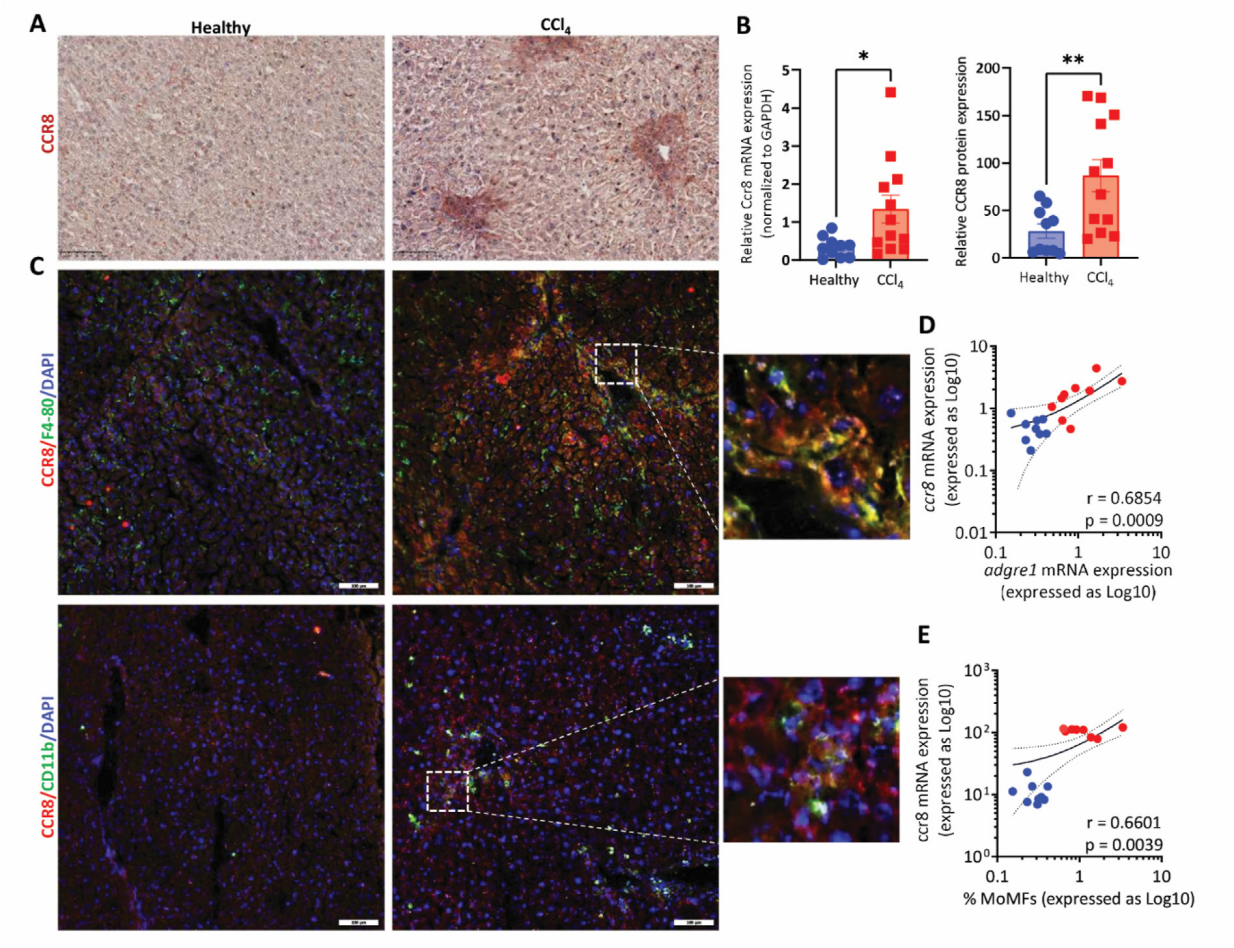
Correlation of CCR8 expression with the migration and differentiation of hepatic macrophages during liver injury. (A) Representative images (scale bar = 100 μm) of CCR8-stained liver sections from healthy mice, *n* = 10 and CCl4 mice, *n* = 12. (B) Quantitative analysis of protein expression and relative mRNA expression of CCR8 in the liver tissues from healthy mice (*n* = 10) and CCl4 mice (*n* = 12). (C) Representative composite images (scale bar = 100 μm) of liver sections from healthy mice, *n* = 10 and CCl4 mice, *n* = 12 stained with CCR8 (red), F4/80 or CD11b (green), and DAPI (nuclear staining, blue). (D) Correlation plot between *Ccr8* mRNA expression and *Adgre1* (F4/80) mRNA expression. (E) Correlation plot between *Ccr8* mRNA expression and% CD11b^++^F4/80^+^ MoMFs population. Results are presented as mean ± SEM. ∗*P* < 0.05, ∗∗*P* < 0.01; *P* denotes statistical significance, and *r* represents Pearson correlation coefficient.

Previous studies have shown that chemokine receptor CCR8 controls the CCL1-directed migration and differentiation of (hepatic) macrophages^16^^,^^18^. In our study, we first analyzed the CCR8 (protein and mRNA) expression in the livers of healthy and CCl_4_-induced acute injury mice. Consistent with the previous study^16^, we observed that protein and gene expression of CCR8 is upregulated during acute liver injury suggesting the relevance of CCR8 pathway in liver injury (Fig. 1A and B). CCR8 expression is localized in the regions of liver injury where (infiltrated) immune cells reside as observed previously^16^ (Fig. 1A). Next, we performed co-immunostainings of CCR8 and F4/80, and CCR8 and CD11b to examine the localization of CCR8 on macrophages (F4/80-positive) and/or on monocytes-derived macrophages (CD11b-positive). We observed a clear co-localization of CCR8 with F4/80 and CD11b confirming that CCR8 is expressed by (infiltrated) macrophages upon liver injury (Fig. 1C and Supporting Information Fig. S1). Notably, CCR8 and CD11b is negligibly expressed in healthy mouse liver sections. We further correlated the expression of *Ccr8* with the expression of *Adgre1* (F4/80 gene, pan macrophage marker) indicative of total hepatic macrophages and found a significant correlation between *Ccr8* and *Adgre1* (*r* = 0.6854; *P* = 0.0009) further confirming that CCR8 is expressed on hepatic macrophages during liver injury (Fig. 1D). Finally, we analyzed the correlation between CCR8 and CD11b^++^ F4/80^+^ infiltrated monocytes-derived macrophages (MoMFs) determined by flow cytometric analysis. We observed a clear correlation between *Ccr8* expression and MoMFs (*r* = 0.6601; *P* = 0.0039) as evidenced by two clusters—low *Ccr8* expression (healthy mice) correlate with low numbers of MoMFs and high *Ccr8* expression (mice with acute liver injury) correlate with high numbers of MoMFs—suggesting that CCR8 is highly expressed on infiltrated MoMFs (Fig. 1E). Altogether, these results indicate that CCR8 is strongly upregulated during liver injury and is expressed on hepatic macrophages that have migrated to the injured liver indicating the role of CCR8 on the migration and differentiation of hepatic macrophages during liver injury.

### 3.2 CCR8 antagonizing peptides

To antagonize the function of CCR8 in order to inhibit the CCL1-driven infiltration of monocytes during liver damage, we designed CCR8 antagonizing peptides. For designing the CCR8 antagonizing peptides, we studied the structure of CCR8, and CCR8 ligands. The *Ccr8* gene encodes a seven transmembrane (TM) CCR8 protein that is a member of G protein-coupled receptor family, and vMIP-I, vMIP-II, MC148 and I-309/CCL1 are recognized as CCR8 ligands^26^. We designed four CCR8 peptides (6–9 amino acids long) based on the protein sequence homology between CCR8 ligands (vMIP-1, v-MIP-II, MC148 and I-309/CCL1) and how these ligands interact with CCR8. The sequences of the designed CCR8-antagonizing peptides (AP8i-iv), their primary structure and basic properties are given in Table 2. Previous studies reported that non-peptide antagonists share common pharmacophore containing central positive nitrogen that interacts with the glutamic acid (Glu286) at the extracellular part of TM7 of CCR8^45^^,^^46^. Hence, in AP8i, AP8ii and AP8iv peptides, we have introduced an arginine that has a positively charged guanidino group, while a lysine residue is introduced in AP8iii. Moreover, flanking groups attached to the positive nitrogen interacts with the chemokine-specific aliphatic and aromatic residues^46^ therefore we introduced Phe (F), Typ (W), Tyr (Y) residues to facilitate the interaction between the peptide antagonists at the CCR8 active site. Following the successful design and synthesis of peptides, we evaluated the interaction of the peptides and R243 with CCR8 *in silico* by homology modeling, docking and molecular dynamics (MD) simulation studies, and *in vitro* in macrophages using fluorescently labeled peptides.

**Table 2:** CCR8 antagonizing peptides and their primary structure and properties (mass and charge).

Name	Peptide sequence	Number of amino acids	Primary structure	Mass	Net charge
AP8i	LDWRHQFIG Leu-Asp-Trp-Arg-His-Gln-Phe-Ile-Gly	9		1170.6	0
AP8ii	YEWRFYHG Tyr-Glu-Trp-Arg-Phe-Tyr-His-Gly	8		1156.5	0
AP8iii	EFHKDWG Glu-Phe-His-Lys-Asp-Trp-Gly	7		917.4	−1
AP8iv	EWRFKG Glu-Trp-Arg-Phe-Lys-Gly	6		821.4	+1

### 3.3 In silico homology modeling, docking and molecular dynamics simulation analysis of CCR8-antagonizing peptides

**Figure 2:**
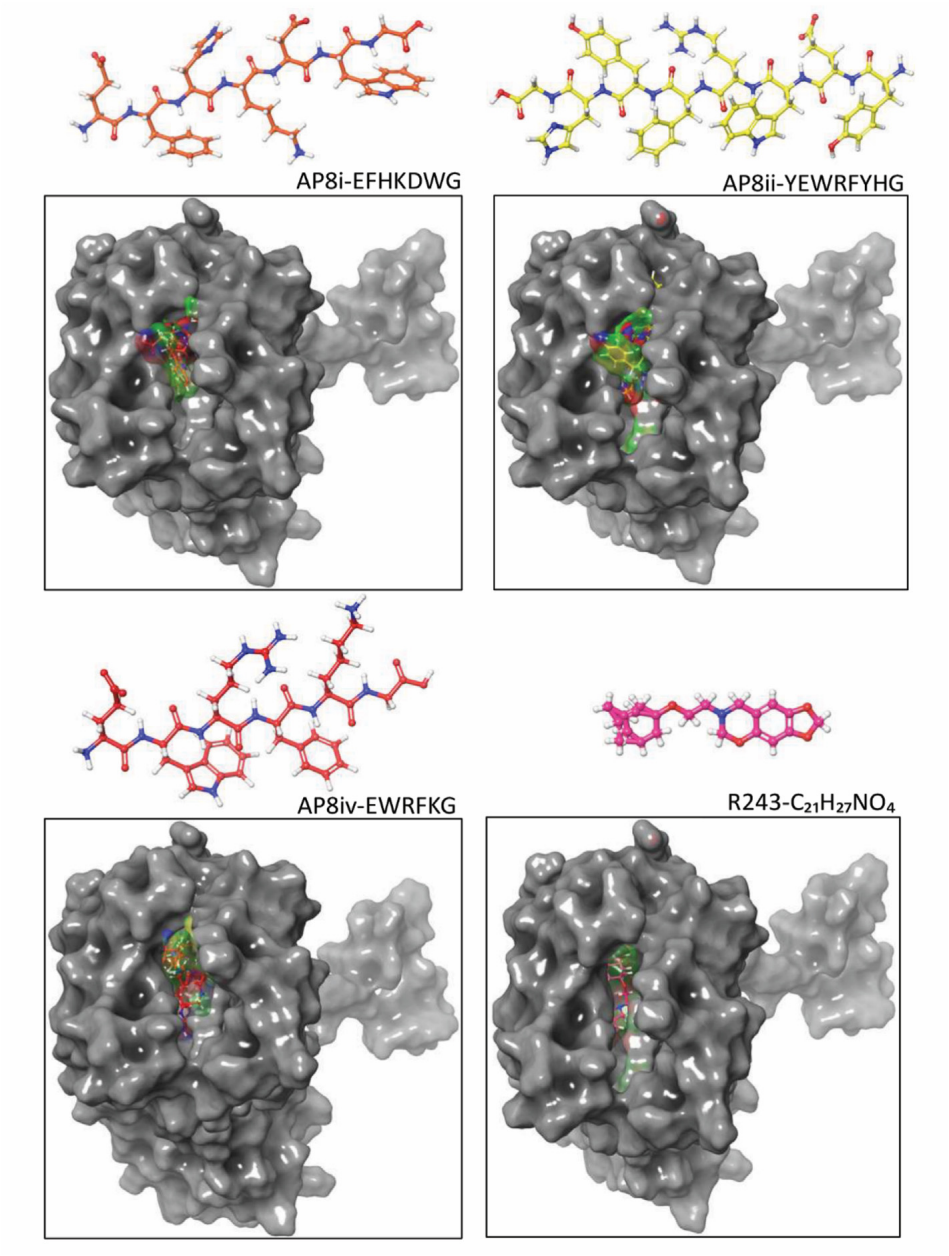
3D structures and docking of peptides: AP8i (orange), AP8ii (yellow), AP8iv (red) and small molecule R243 (magenta), and their respective 3D docking visualization at the active site of CCR8 (Homology build model).

**Figure 3:**
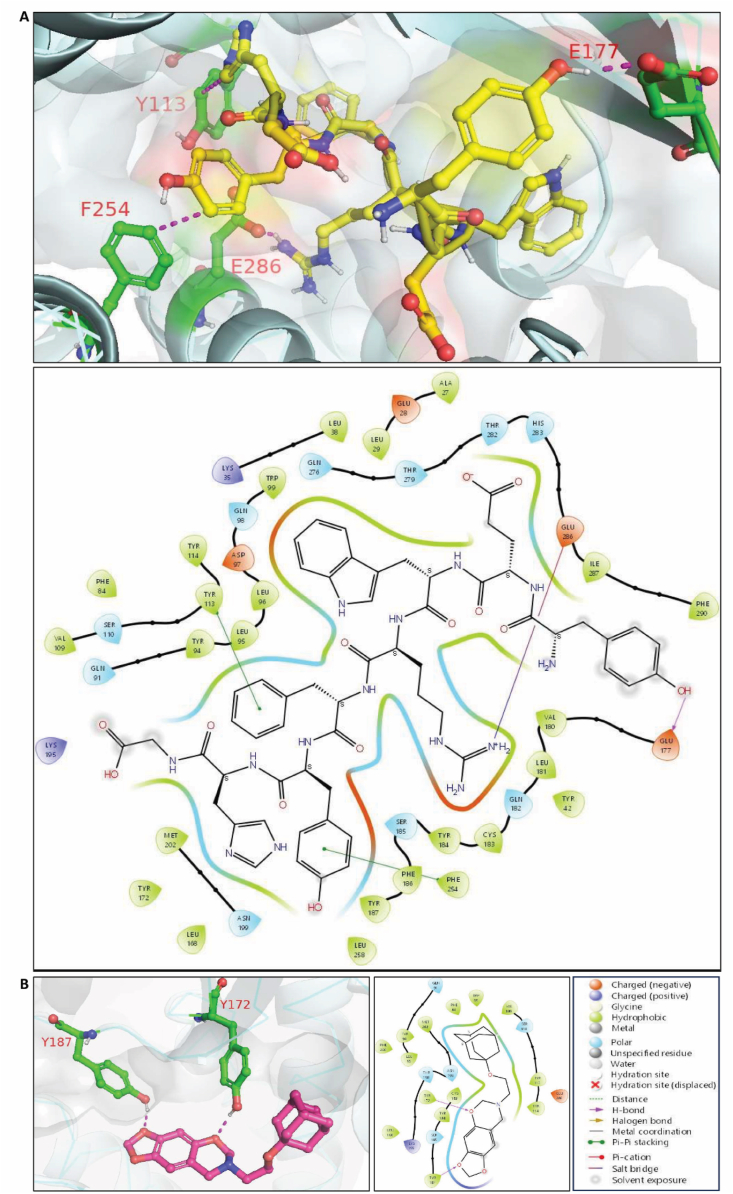
3D and 2D docking poses of (A) AP8ii and (B) R243 at the CCR8 active site based on the homology build model.

Since X-ray crystal structure of CCR8 protein is not available, we first built a homology model of CCR8 as reported earlier by Gadhe et al.^46^. Based on the homology modeling, docking and MD simulation studies of CCR8, the following amino acid residues at CCR8 active site—Gln91, Tyr94, Cys106, Val109, Tyr113, Cys183, Tyr184, Ser185, Lys195, Thr198, Asn199, Met202, Phe254, and Glu286—are proposed to play a role in CCR8 antagonism^46^. The docking of all four designed peptides (AP8i, AP8ii, AP8iii, AP8iv) along with small molecule R243, a selective CCR8 antagonist, was performed at the active site of CCR8 (homology build model). R243 and all AP8 peptides (AP8i, AP8ii and AP8iv), except AP8iii that contains Lys instead of Arg (3D structure shown in Supporting Information Fig. S2) and could not enter the active site, successfully occupied the CCR8 active site (Fig. 2). Based on the homology modeling studies, AP8ii revealed highly favorable interactions with the important amino acid residues present in the CCR8 active site, as shown in Fig. 3A and B. AP8ii showed the hydrogen bonding interaction with Glu177 and *π*-*π* interactions with Tyr113 and Phe254. Furthermore, the aromatic hydrogen bond and salt bridge interactions were visualized with Glu286, in addition to interactions with Leu38, Leu95, Asp97, Leu168, Tyr184, and Lys195 (Fig. 3A and B). Coulombic interaction calculations suggested the crucial roles of Glu286, Lys195, and Tyr113 in CCR8 antagonism^46^ implying that AP8ii peptide exhibit conserved interactions with the important amino acid residues responsible for CCR8 antagonism. Interestingly, similar results and trends of APii-CCR8 interactions were observed using different software such as Mdock PEP and Piper further validating our results (Supporting Information Fig. S3). In comparison, although R243 showed a proper fit in the active site of CCR8, it didn't show stronger interactions necessary for CCR8 antagonism. In fact, R243 molecule displayed interactions (hydrogen bonding) only with Tyr172 and Tyr187 (Fig. 3B). AP8i and AP8iv possessed only fewer interactions at the CCR8 active site (Supporting Information Fig. S4). Altogether these results suggest that among four CCR8-antagonizing peptides (AP8i-AP8iv), AP8ii showed favorable interaction with CCR8, and that AP8ii interacts with amino acid residues that are involved in CCR8 antagonism.

**Figure 4:**
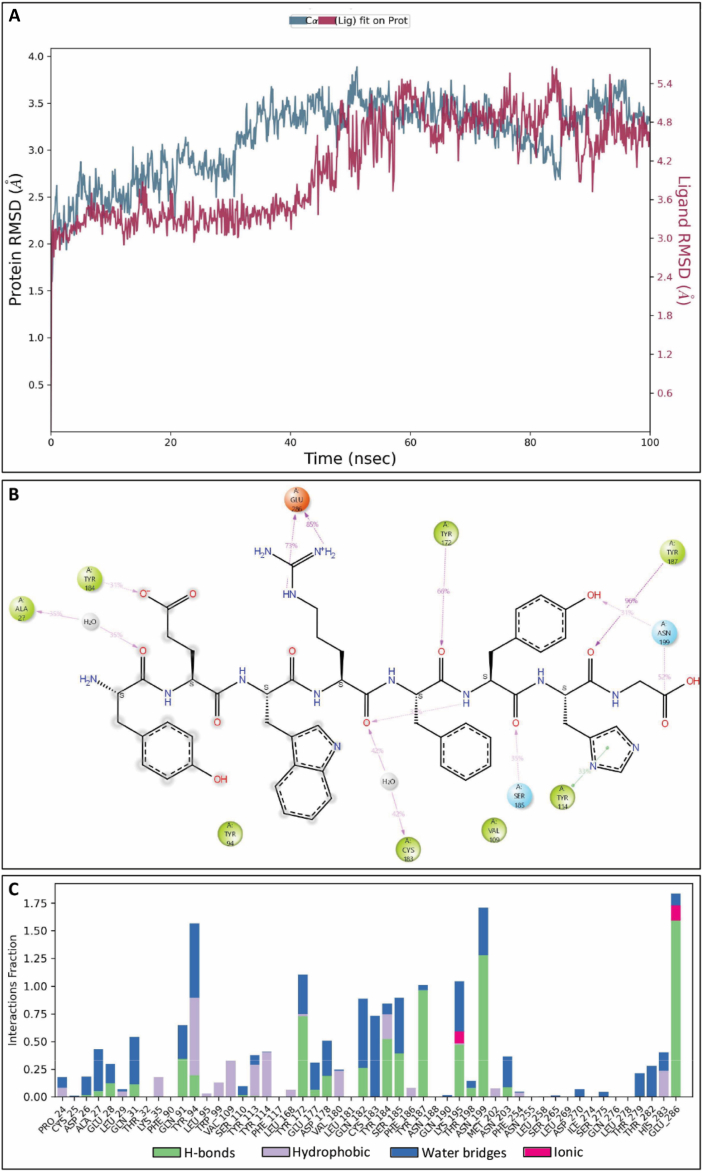
Molecular dynamic simulation studies of AP8ii at the active site of CCR8 (Homology Build Model). (A) RMSD graph showing AP8ii interaction at the CCR8 active site (Homology Build Model) for 100 ns. (B) 2D MD simulation pose of AP8ii at the active site of CCR8 (Homology build model). (C) Interactions (fraction) of peptide AP8ii at the CCR8 active site *via* MD simulation at 100 ns showing the type of Protein–Ligand contacts.

Next, we performed molecular dynamics simulations (MD) using peptide AP8ii complex with CCR8 (homology build model) to explore receptor–peptide interactions and determine the thermodynamic stability of the docked peptide at the active site of the CCR8 protein. Representative system prepared for simulation is shown in Supporting Information Fig. S5A. The simulations were conducted for 100 ns, and the interaction patterns between the AP8ii peptide and amino acids were analyzed. During the MD simulations, stable trajectories were observed for AP8ii with CCR8 in the initial 50 ns. This was evident when examining the temporal changes in potential energy and the protein–ligand complex's root-mean-square deviation (RMSD), as depicted in Fig. 4A. The stabilized range 1.8–3 Å of protein–ligand interaction was observed during 0–50 ns throughout the simulation study. The fluctuation was seen between the protein–ligand complex in the trajectory between 60 and 100 ns and there was a sudden increase in the RMSD possibly due to conformational changes following initial protein–ligand interactions (Fig. 4A). Notably, no substantial structural alterations were observed, and the peptide's conformation remained consistently oriented within the active site of CCR8 whereby AP8ii interacts with Glu286, Asn199, Tyr187, Ser185, Tyr184, Cys183, Tyr172, Tyr114, Val109, Tyr94 and Ala27 (Fig. 4B). Furthermore, the stable interactions of AP8ii with important amino acids Tyr94, Tyr172, Tyr187, Lys195 and Asn199 with ≥1 fraction interactions were visualized and AP8ii retained almost 100% of stable interaction with Glu286 throughout the simulation time, as shown in Fig. 4C and Fig. S5A. Throughout the MD simulations, peptide AP8ii preserved most of the interactions observed in the docking studies while forming new interactions with various amino acid residues (Fig. 4C and Fig. S5A). Protein–ligand contacts (number and density) of AP8ii with CCR8 in each trajectory frame and root-mean square fluctuation (RMSF) graph of protein CCR8 at 100 ns MD simulation are shown in Fig. S5B. RMSF values show the general movement of each residue during the simulation time. The protein–ligand contacts are shown as green lines matching the residue index (Fig. S5B).

**Figure 5:**
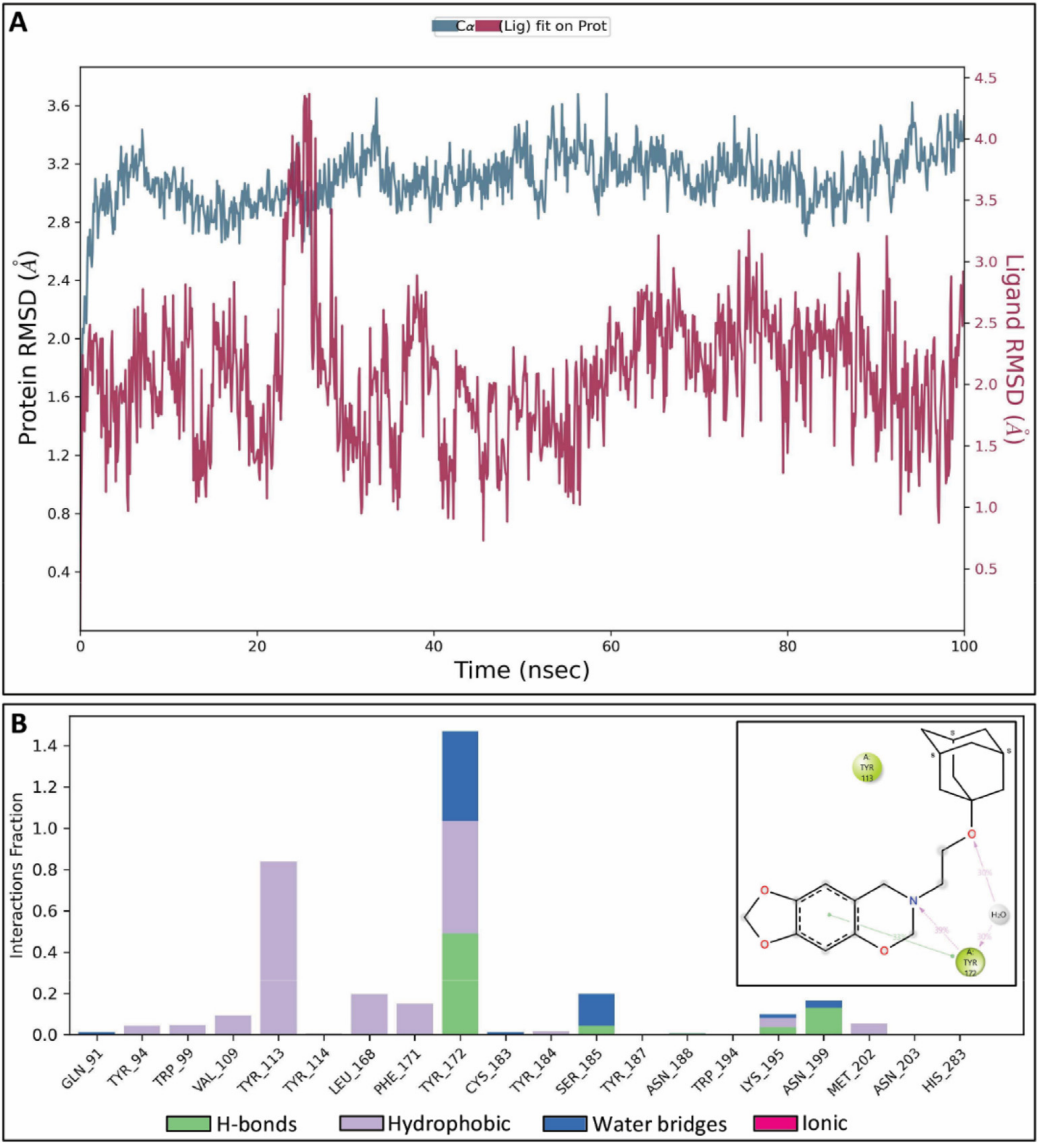
Molecular dynamic simulation studies of R243 at the active site of CCR8 (Homology Build Model). (A) RMSD graph showing R243 interaction at the CCR8 active site (Homology Build Model) for 100 ns. (B) Interactions (fraction) of R243 at the CCR8 active site *via* MD simulation at 100 ns showing the type of Protein–Ligand contacts. Insert: 2D MD simulation pose of R243 at the active site of CCR8 (Homology build model).

Similarly, MD was performed for small molecule CCR8 antagonist, R243, in complex with CCR8 (homology build model) for 100 ns using the same protocol as AP8ii, and the interaction patterns between the test ligand and different amino acids were analyzed as shown in Fig. 5. Representative system prepared for simulation is shown in Supporting Information Fig. S6A. During the MD simulations, stable trajectories were observed for R243 with CCR8 in the initial 0–20 ns and then deviated for 20–30 ns, leading to further increase in RMSD, thereafter gets stabilized (Fig. 5A). This became apparent when observing the time-dependent changes in potential energy and the protein–ligand complex's RMSD. Notably, there were no notable structural alterations, and the ligand's position consistently retained a similar orientation within the active site of CCR8, as confirmed in the docking studies (Fig. 5B). Furthermore, the stable interactions of R243 with Tyr172 and Tyr113 was visualized throughout the simulation time as shown in Fig. 5B and Fig. S6A. The stabilized range 1 Å to 3 Å of protein–ligand interaction was observed throughout the simulation study except in between 20 and 30 ns that deviates above 3 Å, indicating less stability compared to AP8ii. Protein–ligand contacts (number and density) of R243 with CCR8 in each trajectory frame and RMSF graph of protein CCR8 at 100 ns MD simulation are presented in Fig. S6B.

These results show that AP8ii possesses stable interaction with CCR8 involving multiple interactions with amino acid residues present at the active site of the CCR8 as depicted in Supporting Information Fig. S7A and S7B showing the snapshot of AP8ii-CCR8 and R243-CCR8 complex.

To further validate the *in silico* predictions and to confirm the importance of Tyr113 residue in the interaction of R243 and AP8ii with CCR8 antagonism and considering the crucial role of tyrosine residues in protein–ligand interactions, we performed docking analysis following Tyr113 mutation. When Tyr113 is mutated to Ala113, it involves substituting a larger, aromatic amino acid (tyrosine) with a smaller, non-aromatic one (alanine). This alteration could potentially impact the shape of the binding pocket and its interactions with receptor antagonists. Following mutation studies (Tyr113→Ala113), as suggested by Jensen et al.^47^, we observed loss of interaction between CCR8 and R243 or AP8ii (Supporting Information Fig. S8). This loss implies the critical importance of the specific chemical properties or spatial arrangement of the tyrosine residue for receptor antagonism. For AP8ii, despite the loss of interaction following the substitution of Tyr113 with Ala113, we observed an increase in interactions with other nearby amino acids suggesting that the mutation to alanine could have induced structural changes or altered the local microenvironment. For R243, on the other hand, low affinity interactions were observed with CCR8 (Fig. S8). This insight provides valuable information for understanding the molecular basis of CCR8 antagonism and informs rational drug design targeting CCR8.

Finally, we assessed if AP8ii non-specifically interacts with other CCRs. We performed docking analysis of AP8ii with other CCRs including CCR1, CCR2, CCR5, CCR7, and CCR9. We observed no docking pose of AP8ii at the active sites of CCR1, CCR7, and CCR9, and lower docking scores with CCR2 and CCR5 indicative of low affinity interactions whereby AP8ii showed limited interactions with crucial amino acid residues when compared with the co-crystallized ligand (Supporting Information Fig. S9).

### 3.4 *In vitro* binding of CCR8 antagonizing peptides with LPS/IFNγ-activated mouse RAW264.7 macrophages

**Figure 6:**
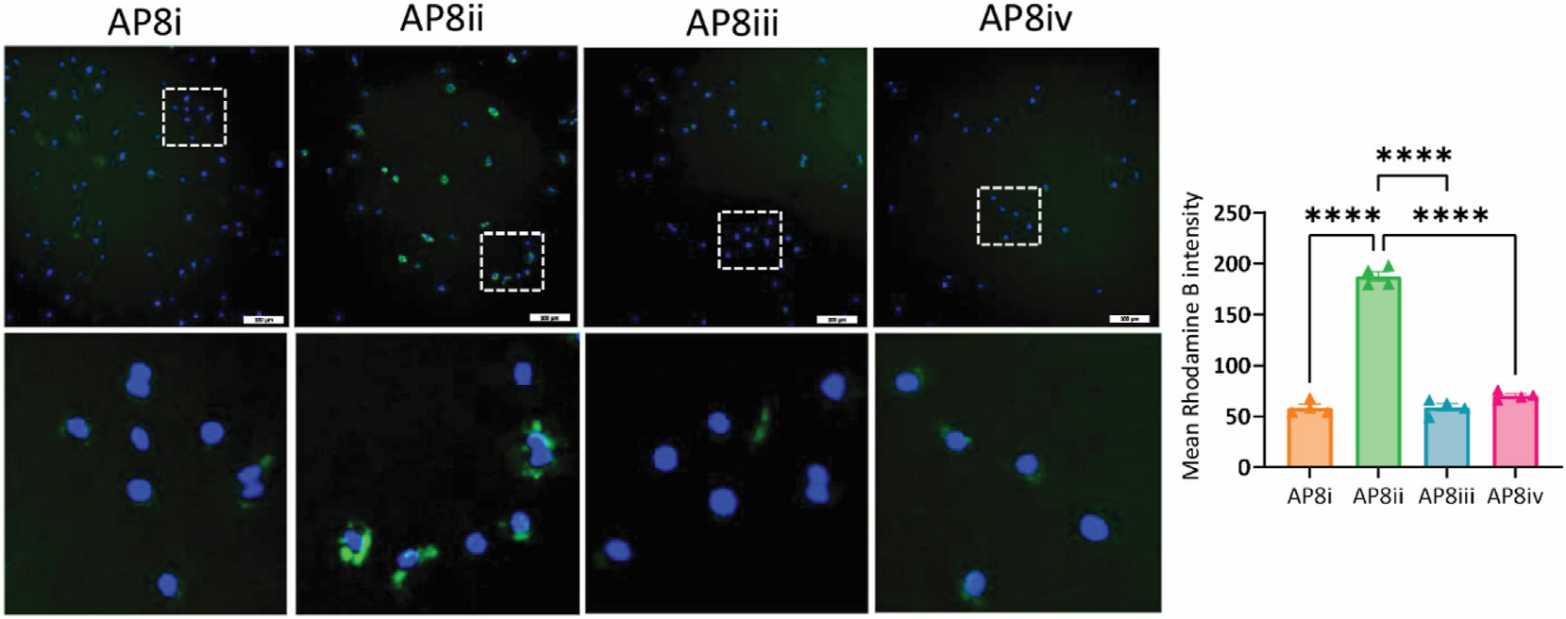
*In vitro* binding analysis of CCR8 antagonizing peptides on LPS/IFN*γ*-activated RAW264.7 macrophages. Representative images and quantitative analysis of AP8i, AP8ii, AP8iii, and AP8iv binding to LPS/IFN*γ*-activated RAW264.7 macrophages after 4 h of incubation at room temperature. The composite and zoomed in image (indicated with the dotted white box) showing DAPI-stained cells (nuclei are stained blue with DAPI) and RhB-labeled peptides (in green). Results from 4 independent experiments (performed in triplicates) are presented as mean ± SEM. ∗∗∗∗*P* < 0.001; One-way ANOVA with Bonferroni *post hoc* test.

To further confirm the results of the docking studies whereby AP8ii (among the peptides) showed the most favorable interactions at the active site of CCR8, we performed the binding studies of all four CCR8 antagonizing peptides with CCR8 expressed on LPS/IFN*γ*-activated mouse RAW264.7 macrophages. For the binding studies, we used rhodamine B (RhB) labeled peptides where RhB was conjugated at the N-terminus of the peptides. LPS/IFN*γ*-activated RAW264.7 macrophages were incubated for 4 h at RT with the RhB-labeled peptides, after which unbound peptides were washed and peptides bound to the macrophages were visualized. DAPI nuclear stain was used to visualize the cells. As can be seen in Fig. 6, we observed highly specific binding and highest fluorescent intensity with AP8ii when compared with the other AP8 (AP8i, AP8iii and AP8iv) peptides confirming our docking results.

### 3.5 Competitive blocking of AP8ii interaction with CCR8, using R243, CCL1 and anti-CCR8 antibodies, on LPS/IFNγ-activated mouse RAW264.7 macrophages

**Figure 7:**
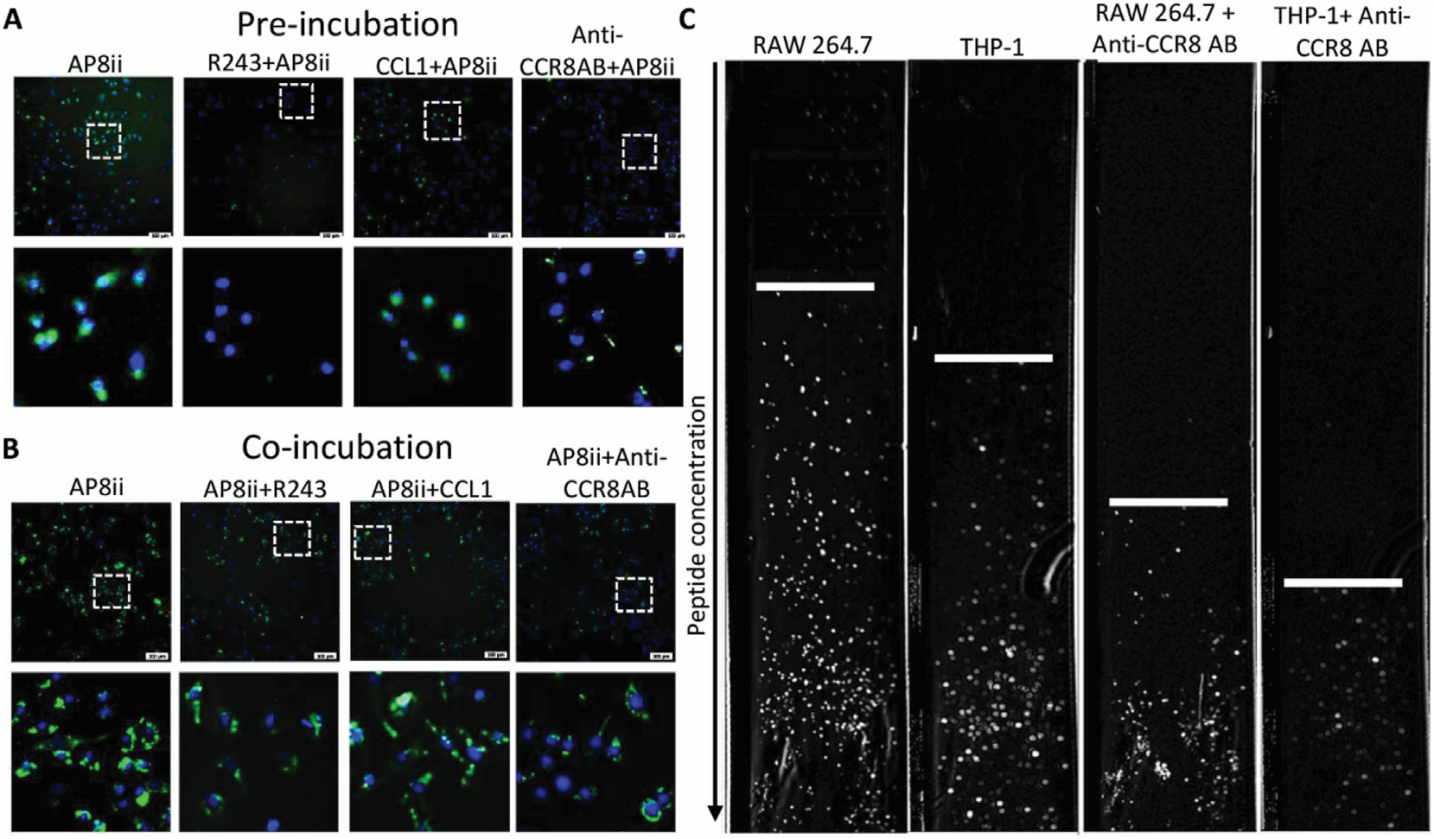
AP8ii–CCR8 interaction analysis. Representative composite and zoomed in images (indicated with the dotted white box) of the composite images of pre-incubation (A) or co-incubation (B) studies performed on LPS/IFN*γ*-activated RAW264.7 macrophages. RhB-labelled AP8ii (10 μmol/L) that was either co-incubated with R243 (10 μmol/L), CCL1 (100 ng/mL), or the anti-CCR8 antibody (5.0 μg/mL) for 4 h or R243 (10 μmol/L), CCL1 (100 ng/mL), or the anti-CCR8 antibody (5.0 μg/mL) was preincubated for 2 h followed by incubation with AP8ii (10 μmol/L) for 4 h. (C) Surface Plasmon Resonance analysis using the CellVysion SPR imaging system. The first two columns show the peptide interaction with LPS/IFN*γ*-activated RAW264.7 and THP-1 macrophages, while the last two columns show peptide binding following anti-CCR8 antibody blocking. Results from 6 independent experiments (performed in duplicates) are presented as mean ± SEM. ∗*P* < 0.05, ∗∗*P* < 0.01; One-way ANOVA with Bonferroni *post hoc* test.

In a previous study, Gadhe et al.^46^*,* showed the involvement of Gln91, Tyr94, Cys106, Val109, Tyr113, Cys183, Tyr184, Ser185, Lys195, Thr198, Asn199, Met202, Phe254, and Glu286 in CCL1 interaction. Moreover, previous mutational study with 29 mutations showed that Gln91, Tyr113, Trp194, Gly206, and Phe254 are important for the CCL1 binding, whereas Tyr42, Phe88, Gln91, Tyr113, Phe254, Leu258, Ser262, and Glu286 are important for the binding of non-peptide agonists^47^. Based on *in silico* homology modeling, docking and MD simulation study, we postulated that AP8ii might interact with amino acid residues that are partly involved in the CCL1 binding and non-peptide CCR8 agonists/antagonists. We therefore questioned if indeed AP8ii interacts partly at the same site as CCL1, R243 or CCR8 antibody *in vitro*. To answer this question, we performed two competitive peptide binding studies – co-incubation and pre-incubation using LPS/IFN*γ*-activated RAW264.7 macrophages. For these studies, RhB-labeled AP8ii (10 μmol/L) was either co-incubated with R243 (10 μmol/L), CCL1 (100 ng/mL), or anti-CCR8 antibody (5.0 μg/mL) for 4 h or R243 (10 μmol/L), CCL1 (100 ng/mL), or the anti-CCR8 antibody (5.0 μg/mL) was pre-incubated for 2 h followed by incubation with AP8ii (10 μmol/L) for 4 h. As can be seen in Fig. 7A and B, the intensity of AP8 is significantly reduced after co- and pre-incubation with R243, CCL1 and anti-CCR8 antibody suggesting R243, CCL1, anti-CCR8 antibody occupy the AP8ii binding site. The effects are more pronounced in pre-incubation compared to co-incubation studies. In the pre-incubation study, R243, CCL1 and anti-CCR8 blocked about 56%, 30% and 53% of AP8ii binding respectively while in co-incubation study, R243, CCL1 and anti-CCR8 blocked about 27%, 20% and 35% of AP8ii binding respectively (Supporting Information Fig. S10). Notably, AP8ii binding was more strongly blocked by R243 and anti-CCR8 antibody compared to CCL1 indicating that AP8ii interacts at the similar site as R243 and anti-CCR8 antibody. These results confirm our docking and MD simulation analysis suggesting indeed AP8ii possess favorable interactions with CCR8, and that our antagonizing peptide AP8ii is an excellent candidate for antagonizing CCR8.

### 3.6 Surface plasmon resonance analysis confirms selective AP8ii interaction with CCR8

To confirm our peptide interaction studies, we performed SPR analysis using the CellVysion SPR imaging system. We coated the SPR sensor with the gradient of AP8ii peptide after which we evaluated the cell interaction expressing CCR8 receptor with or without blocking with anti-CCR8 antibodies. For these experiments, we activated RAW264.7 and PMA-polarized human THP-1 macrophages with LPS and IFN*γ*. To block the CCR8, we incubated the cells with species-specific anti-CCR8 antibodies for 4 h. We observed a clear interaction between LPS/IFN*γ*-activated RAW264.7 macrophages which could be strongly blocked by anti-CCR8 antibodies indicating CCR8-specific binding of AP8ii as evidenced by considerable change in the tipping point of cell binding (Fig. 7C). THP-1 macrophages showed lower interaction with AP8ii peptide which could be attributed to lower CCR8 expression levels on THP-1 macrophages compared to RAW macrophages. Nevertheless, interaction between AP8ii peptide with THP-1 macrophages was blocked by anti-CCR8 antibodies suggesting CCR8-specific binding of AP8ii (Fig. 7C).

### 3.7 AP8ii inhibits CCL1-induced transwell migration in mouse RAW macrophages, human THP-1 monocytes, and freshly isolated primary mouse bone-marrow derived macrophages *in vitro*

**Figure 8:**
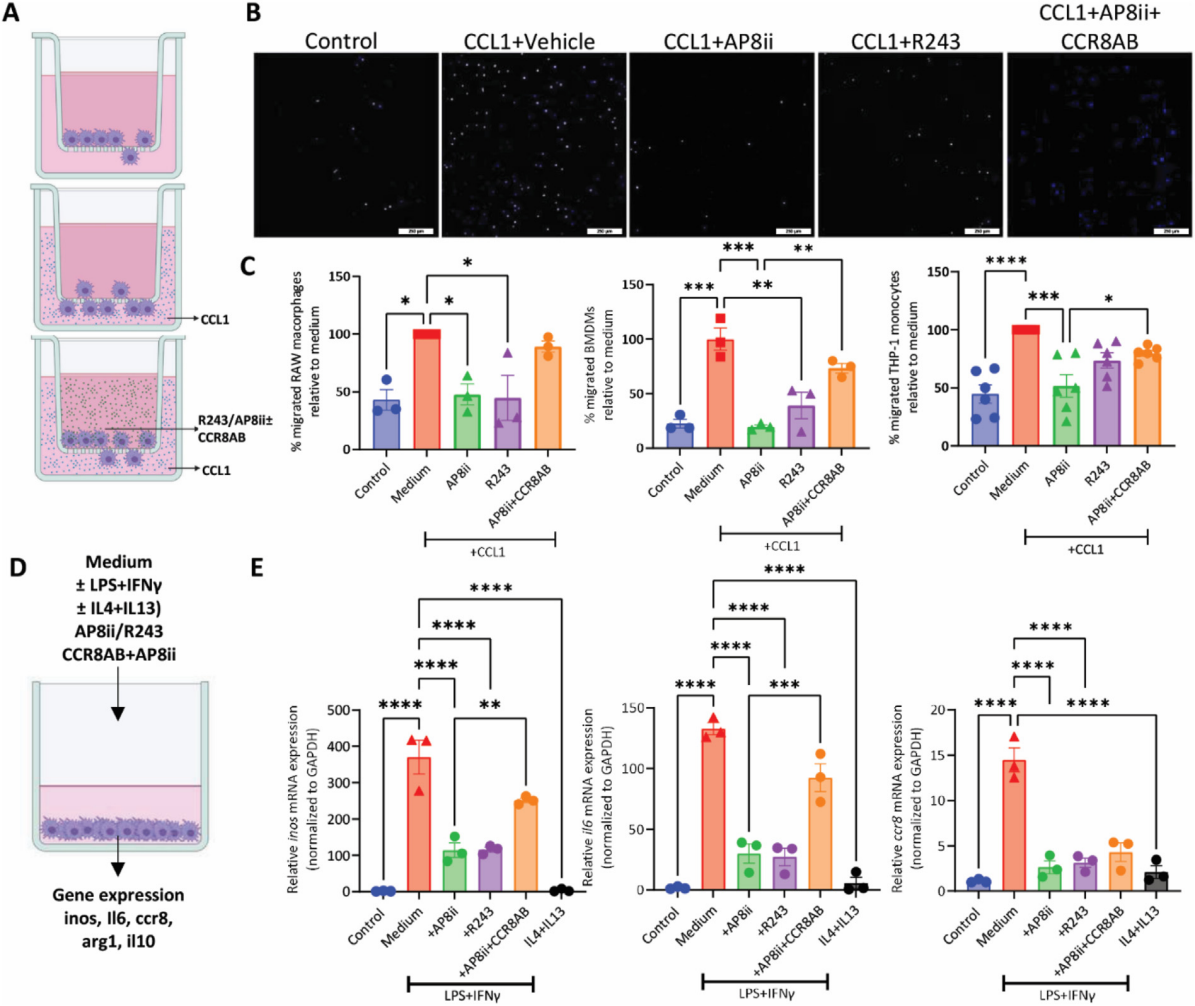
AP8ii inhibits CCL1-induced migration and LPS/IFN*γ*-induced activation of macrophages *in vitro*. (A) Schematic representation of CCL1-induced migration assay; macrophages/monocytes are seeded in the Transwell insert with or without AP8ii, R243 or DMSO (10 μmol/L), and CCL1 (10 ng/mL) is added in the lower well. (B) Representative pictures of migrated nuclei (DAPI)-stained RAW macrophages. (C) Analyzed results of the migration assay performed on mouse RAW macrophages (*n* = 3), BMDMs (*n* = 3) and human THP1 monocytes (*n* = 6). (D) Schematic showing the experimental setup for the experiment. (E) Graphs showing the gene expression analysis of *Inos*, *Il6*, and *Ccr8* performed on mouse RAW264.7 macrophages. The cells were activated with 100 ng/mL LPS + 10 ng/mL IFN*γ* with and without 10 μmol/L of CCR8 antagonizing peptide (AP8ii) or CCR8 antagonist (R243) with anti-CCR8 antibodies (CCR8AB) or 10 ng/mL IL4 and 10 ng/mL IL13 for 24 h followed by gene expression analysis. All results are presented as mean ± SEM from three independent experiments. ∗*P* < 0.05, ∗∗*P* < 0.01, ∗∗∗*P* < 0.001, ∗∗∗∗*P* < 0.0001, One-way ANOVA with Bonferroni *post hoc* test.

We next evaluated the therapeutic efficacy of our antagonizing peptide AP8ii. We used a Transwell migration assay to examine the effect of AP8ii on the CCL1-induced migration of monocytes-macrophages (depicted in Fig. 8A). LPS/IFN*γ*-activated mouse RAW264.7 macrophages, freshly isolated primary murine bone-marrow derived macrophages (BMDMs), and human THP-1 monocytes were seeded in the Transwell inserts while CCL1 was added in the lower chamber to induce migration. 10 μmol/L AP8ii, R243 or the corresponding amount of DMSO (DMSO is used to dissolve AP8ii and R243) was supplemented in the Transwell insert as CCR8 antagonist interacts with CCR8 expressed on monocytes-macrophages. After 24 h, the migrated cells are counted and compared to the vehicle (DMSO) group (set at 100%). Results show that CCL1 significantly upregulated the Transwell migration of mouse RAW macrophages, BMDMs and human THP-1 monocytes (Fig. 8B and C). CCR8 antagonism using our antagonizing peptide decreased this Transwell migration in mouse RAW macrophages, BMDMs as well as human THP-1 monocytes (Fig. 8B and C). R243, on the other hand, induced a significant reduction in the migration of RAW macrophages and BMDMs, not in human THP-1 monocytes. Notably, addition of anti-CCR8 antibodies rescued the inhibitory effect of AP8ii indicating that the macrophage migration is induced *via* CCL1‒CCR8 axis (Fig. 8B and C). Anti-CCR8 antibodies did not inhibit macrophage migration suggesting that CCR8 antibody blocks the interaction of AP8ii with CCR8 while do not antagonize CCR8 function. Altogether, these results demonstrate that our antagonizing peptide AP8ii inhibits the migration of both mouse and human monocytes/macrophages *via* CCR8 antagonism.

### 3.8 AP8ii inhibits LPS/IFNγ-induced activation of mouse RAW macrophages *in vitro*

We further evaluated the efficacy of AP8ii peptide on the activation of RAW264.7 macrophages *in vitro* (Fig. 8D)*.* We observed that AP8ii significantly inhibited the gene expression of major inflammatory markers (inducible nitric oxide synthase, *Inos* and Interleukin 6, *Il6)* in LPS/IFN*γ*-activated RAW macrophages (Fig. 8E). To note that AP8ii did not activate RAW macrophages in steady non-activated state (Supporting Information Fig. S11). In addition, AP8ii inhibited *Ccr8* gene expression in both non-activated and LPS/IFN*γ*-activated macrophages (Fig. 8E and Fig. S11). Similar to AP8ii, R243 also inhibited the gene expression of *Inos and Il6* in LPS/IFN*γ*-activated RAW macrophages (Fig. 8E) and induced mild (non-significant) activation of RAW macrophages in steady non-activated state (Fig. S11). Also, R243 significantly inhibited *Ccr8* gene expression in both non-activated and LPS/IFN*γ*-activated macrophages (Fig. 8E and Fig. S11). Importantly, the inhibition by AP8ii peptide was almost completely rescued by co-incubation with anti-CCR8 antibodies indicating AP8ii anti-inflammatory effects are due to CCR8 antagonism (Fig. 8E). Interestingly, the *Ccr8* expression levels inhibited by AP8ii were not rescued by anti-CCR8 antibodies (Fig. 8E). Finally, we evaluated if AP8ii (or R243) polarize the macrophages in anti-inflammatory (M2) phenotype. We therefore analyzed the gene expression of M2-specific markers *i.e.*, interleukin 10 (*Il10)* and arginase 1 (*Arg1)* in non-activated macrophages and in LPS/IFN*γ*-activated macrophages with and without AP8ii and R243 treatment (Fig. S11). We observed no significant induction in the *Il10*and *Arg1* with AP8ii while mild induction with R243 could be seen in non-activated macrophages. In LPS/IFN*γ*-activated RAW macrophages, AP8ii and R243 showed slight reduction in *Arg1* mRNA levels while no change in *Il10* expression levels was observed. For both *Il10* and *Arg1* levels, coincubation with anti-CCR8 antibodies showed opposite effects than AP8ii again confirming that AP8ii effects are mediated *via* CCR8 antagonism (Fig. S11). Anti-CCR8 antibodies did not inhibit macrophage activation confirming our previous results suggesting that CCR8 antibody blocks the interaction of AP8ii with CCR8 while do not antagonize CCR8 function. Altogether these results indicate that AP8ii inhibits activation/polarization of pro-inflammatory macrophages *via* CCR8 antagonism while do not induce anti-inflammatory macrophage differentiation.

### 3.9 AP8ii attenuates the intrahepatic infiltration of monocyte-derived macrophages *in vivo* in acute liver injury mouse model

**Figure 9:**
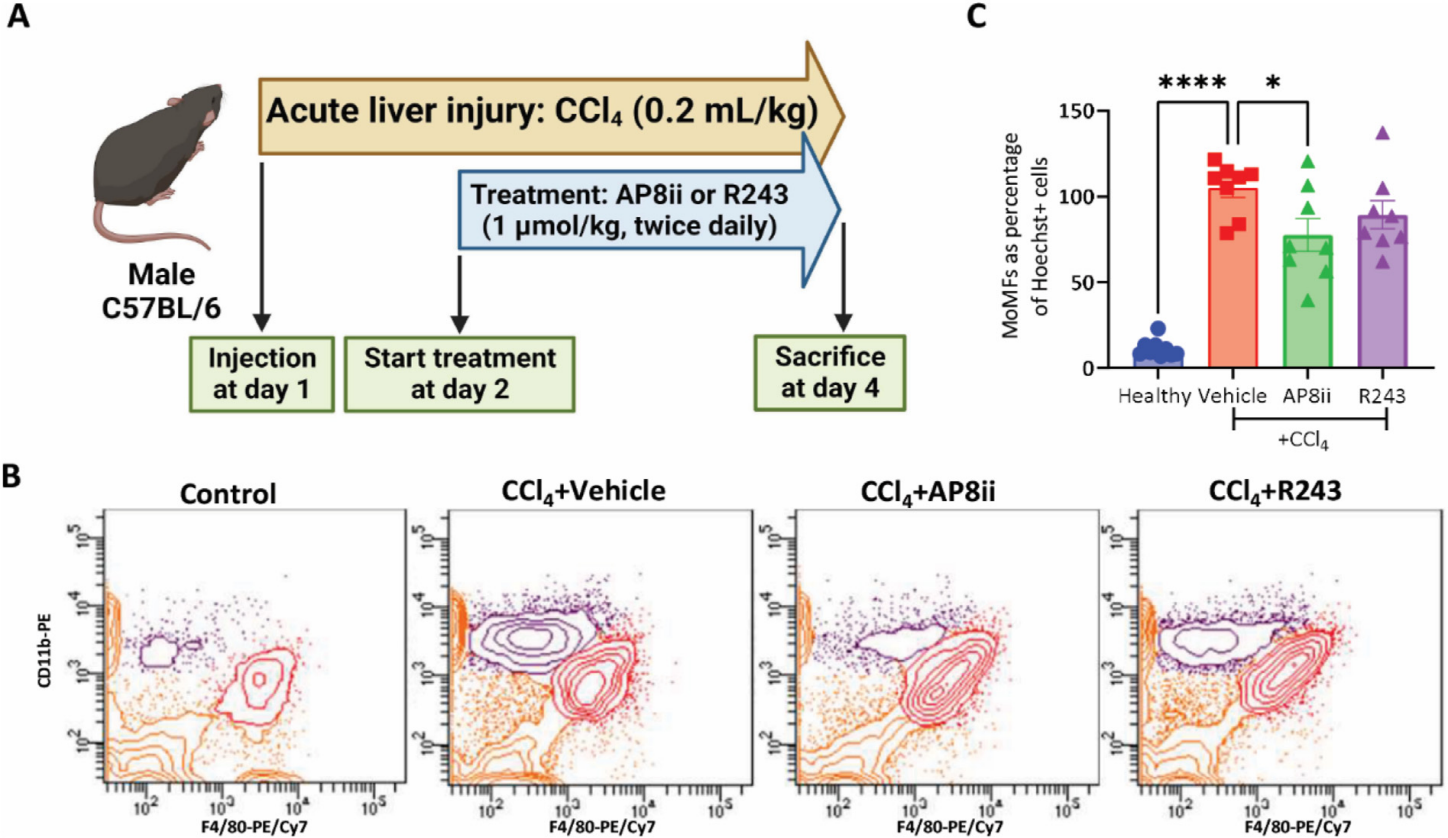
AP8ii inhibits CCL1-induced monocyte migration *in vivo*. (A) Schematic representation of CCl_4_-induced acute liver injury mouse model. (B) Representative flow cytometric (contour) plots of nucleated CD45^+^ cells where MoMFs (in purple) are depicted as CD11b^++^ F4/80^+/−^ events (C) Quantitative analysis showing MoMFs as percentage of Hoechst + cells. All results are presented as mean ± SEM. ∗*P* < 0.05, ∗∗∗*P* < 0.001, ∗∗∗∗*P* < 0.0001, One-way ANOVA with Bonferroni *post hoc* test.

After successful *in vitro* studies, we evaluated the efficacy of AP8ii *in vivo* in a CCl_4_-induced acute liver injury model. Mice were injected with olive oil (healthy, *n* = 9) or 0.2 mL CCl_4_/kg (vehicle, AP8ii or R243, *n* = 8 each) to induce liver injury, followed by 2 days of treatment with 1 μmol/kg AP8ii or 1 μmol/kg R243 twice daily, Fig. 9A. To investigate the infiltration of MoMFs, small pieces of liver were mechanically dissociated, and single cell suspension was stained with Hoechst, CD45-APC, CD11b-PE and F4/80-PE/Cy7 and cells were gated as per the gating strategy explained in the materials and methods section. MoMFs (CD11b^++^ F4/80^+^) shown in purple were identified in different groups (representative flow cytometric plot presented in Fig. 9B) and the quantified results can be seen in Fig. 9C. The results show that the total amount of MoMFs (as% of Hoechst positive cells) in the livers is significantly upregulated during acute liver injury, and AP8ii induced a significant decrease in MoMFs, whereas R243 showed no significant inhibition. The relative amount of CD11b^+^ F4/80^++^ KCs (as% of Hoechst positive cells) did not change significantly in CCl_4_ group, while the absolute amount significantly increased in all the groups, compared to the healthy group (Supporting Information Fig. S12).

### 3.10 AP8ii ameliorates liver inflammation and early fibrosis during acute liver injury *in vivo*

**Figure 10:**
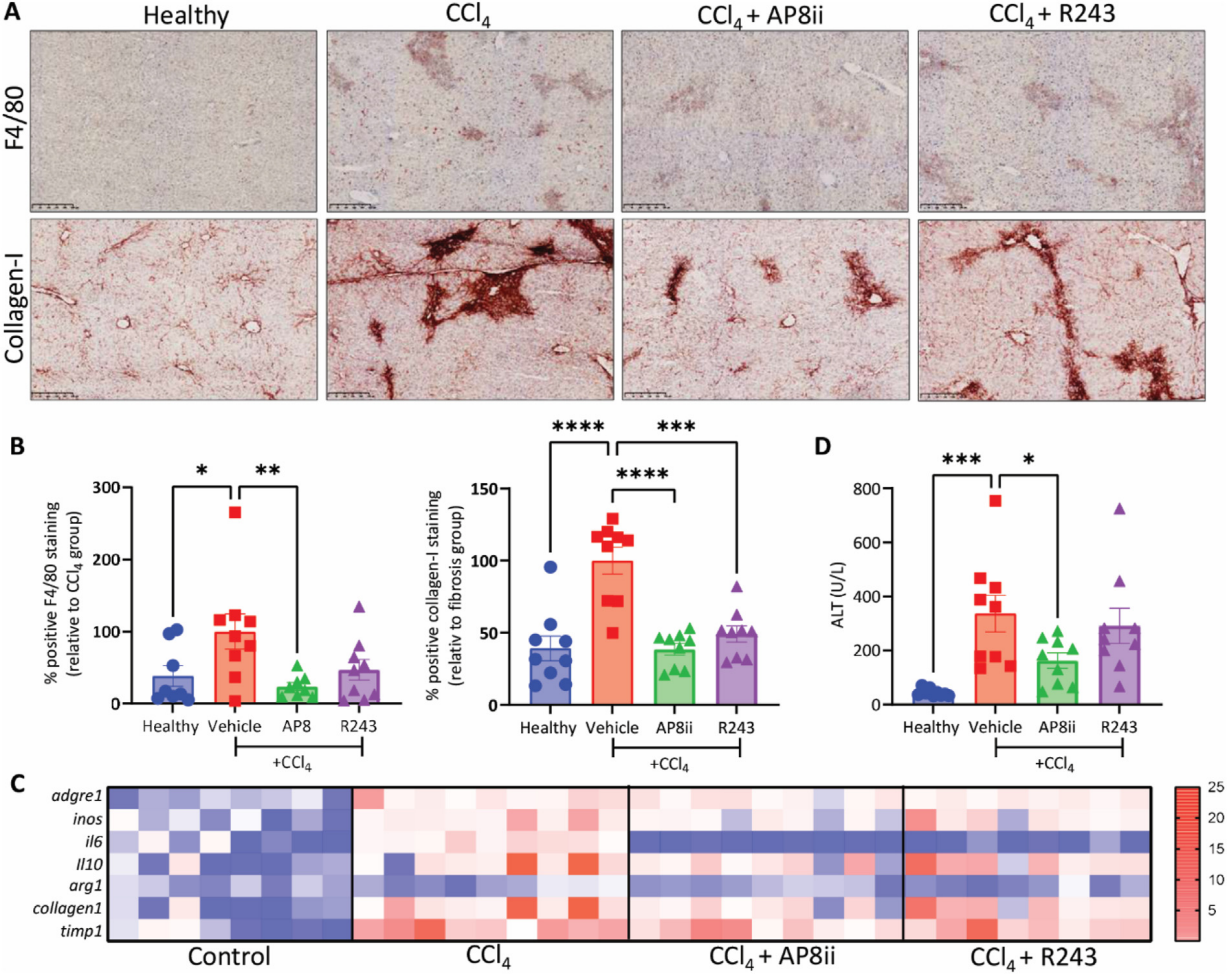
AP8ii ameliorates liver inflammation and early fibrosis during acute liver injury *in vivo*. (A) Representative images and (B) quantitative analysis of F4/80 and collagen-I-stained liver (healthy, vehicle, AP8 and R243 mice, *n* = 9 mice per group) sections. (C) Heat map showing the relative gene expression of *Adgre1* (F4/80), *Il6* (interleukin-6), procollagen1a1 (collagen-I) and *Timp1*, normalized with GAPDH) analysed in the liver (healthy, vehicle, AP8 and R243 mice, *n* = 9 mice per group). All results are presented as mean ± SEM. ∗*P* < 0.05, ∗∗*P* < 0.01, ∗∗∗*P* < 0.001, ∗∗∗∗*P* < 0.0001, One-way ANOVA with Bonferroni *post hoc* test.

Recruited monocytes differentiate into macrophages and become activated into pro-inflammatory macrophages and drive inflammation during liver injury^6^. Our *in vitro* and *in vivo* results showed that AP8ii can effectively inhibit CCL1-driven monocyte recruitment. We next evaluated if reduced monocyte-macrophage recruitment alleviates liver inflammation during acute liver injury. Therefore, we examined the expression of F4/80 (pan-macrophage marker) by immunohistochemical staining on liver sections from healthy and CCl_4_-mice with and without R243 pr AP8ii treatment. As can be seen in Fig. 10A and B, F4/80 staining was increased and was localized in the areas of liver injury, upon CCl_4_-administration, while AP8ii significantly decreased the number of F4/80 positive macrophages in the liver as evidenced by reduced F4/80 staining. This was further supported by gene expression analysis of liver tissues where mRNA levels of *Adgre1* (F4/80 gene) was reduced in AP8ii-treated mice (Fig. 10C and Supporting Information Fig. S13). Since the CCl_4_-administration induced monocyte infiltration before AP8ii treatment, and AP8ii treatment inhibited the subsequent infiltration of monocytes, we questioned if AP8ii inhibits the pro-inflammatory activation/polarization of monocytes/macrophages that infiltrated the liver following liver injury. Aligned with our *in vitro* results, we observed that AP8ii indeed inhibited the pro-inflammatory activation/polarization of monocytes/macrophages as evidenced by the reduced *Inos* (marker for pro-inflammatory macrophages) and *Il6* (pro-inflammatory cytokine) gene expression (Fig. 10C and Fig. S13). We also examined if AP8ii influenced the macrophage polarization into anti-inflammatory M2 phenotype. In line with our *in vitro* studies, we found no significant difference in *Il10* (anti-inflammatory and anti-fibrotic cytokine secreted mainly by alternatively activated M2 macrophages and *Arg1* (M2 marker) (Fig. 10C and Fig. S13).

Besides inflammation, infiltrated MoMFs are also responsible for the onset of fibrosis in the liver^5^^,^^6^. Therefore, we evaluated the expression of collagen-I (fibrosis marker) at protein and mRNA level in the liver tissues. Here again, we saw a significant reduction in collagen-I protein and mRNA expression levels in AP8ii-treated mice indicating amelioration of early liver fibrosis (Fig. 10A–C and Fig. S13). Moreover, we analyzed *Timp1* (tissue inhibitor of metalloproteinases) produced by activated HSCs and hepatic macrophages, known to play a pivotal role in matrix remodeling during liver injury. A previous study revealed that silencing of *Timp1* attenuates HSCs proliferation suggesting the central role of TIMP-1 in liver fibrosis^48^. In our study, we observed that AP8ii inhibited *Timp1* mRNA expression further suggesting attenuation of early fibrosis during acute liver injury (Fig. 9C and Fig. S13). In comparison with AP8ii, R243 showed no or relatively less pronounced effects *in vivo* (Fig. 9A–C and Fig. S13).

### 3.11 Stability and safety profile of AP8ii

To investigate the stability of AP8ii peptide in human serum, analytic HPLC experiments were performed. The peptide was incubated in 10% *v*/*v* human serum at 37 °C for increasing times, after which the sample was quenched in acidic medium, centrifuged to reduce the presence of serum components and analyzed by analytic HPLC. The chromatogram trace of the sample after 24 h of incubation (Supporting Information Fig. S14) shows that the peak at retention time (rt) ∼ 10.6 min corresponds to the intact peptide, while the peaks at rt = 7, 9 and 13 min belong to serum components that remained in solution, even after the centrifugation step. Control experiments run with only peptide and only serum corroborated this peak assignment (see Supporting Information—HPLC analysis). Importantly, the peptide remained stable during the first 4 h of exposure to serum, indicated by the absence of significant differences in the peak absolute areas of the signal at rt ∼10.6 min. Moreover, >82% of peptide remained intact after 24 h of incubation (Fig. S14). Therefore, our results indicate that, under the investigated conditions, the *t*_1/2_ of the AP8ii peptide is larger than 24 h; thus, supporting the high stability of the antagonizing peptide.

Finally, we evaluated the safety profile of our antagonizing peptide both *in vitro* and *in vivo*. *In vitro*, on mouse (RAW264.7 and BMDMs) and human (THP-1) macrophages, the peptide AP8ii showed no significant toxicity in different conditions as assessed by Alamar blue-based metabolic assay (Supporting Information Fig. S15). R243, on the other hand, showed mild toxicity in some of the conditions (Fig. S15). We monitored the body weight of the animals and examined the organ-to-body weight ratio of 5 major organs, liver, lungs, kidneys, spleen, and heart (Supporting Information Fig. S16A–S16G)^49^. We observed no significant difference in body weight and organ-to-body weight ratio in different treatment groups (Fig. S16A–S16F). Importantly, the weight of healthy animals and AP8ii-treated animals increased during treatment, while the weight of vehicle-treated and R243-treated animals decreased. Weight loss could be a sign of decreased appetite, as a result of side effects of treatment, similar to the weight reduction after CCl_4_. Healthy and AP8ii treated mice showed recovery of weight during treatment, while vehicle treated and R243 treated mice did not. Lastly, we analyzed ALT and AST plasma levels as an indicators of liver function, Fig. 9D and Fig. S16I. ALT and AST levels were significantly upregulated in vehicle treated animals and showed a decrease in ALT (and a trend in AST) in AP8ii-treated mice, but no changes in R243-treated mice were observed (Fig. 9D and Fig. S16I). These results indicate a safer and efficient therapeutic effect of CCR8-antagonizing peptide AP8ii compared to small molecule CCR8 antagonist R243.

## 4 Discussion

Upon liver injury, circulating monocytes are recruited to the liver, mature to macrophages and play a crucial role in pathological inflammation and fibrosis during liver disease^4^^,^^8^. In addition to the extensively investigated CCL2/CCR2 axis^50^, an alternative pathway for monocyte recruitment involves the relatively less studied CCL1/CCR8 axis^16^. Studies using *Ccr8*^*−/−*^ mice evidenced notable reduction in the infiltration of immune cells, particularly the pro-inflammatory Ly6C^high^ monocytes, resulting in decreased inflammation and fibrosis during liver injury in mouse models^16^. Notably, in contrast to most CCRs that interact with multiple ligands, CCR8 stands out due to its unique binding to a single agonizing ligand, CCL1, thereby rendering it an exceptionally viable target for interference in monocyte recruitment^31^. Given the specificity and improved pharmacokinetics and bioavailability profile compared to small molecule inhibitors, peptides emerge as a promising candidate for receptor antagonism^51^. In this study, we designed four different CCR8 antagonizing peptides, evaluated their interaction with CCR8 (CCR8 antagonism) *in silico* by homology modeling, docking and MD simulation studies and *in vitro* using macrophages.

Our study confirmed the upregulation of CCR8 at both the mRNA and protein levels during liver injury. Moreover, the increased CCR8 expression correlates with the markers of macrophage-driven inflammation and MoMFs suggesting the involvement of CCR8 in the recruitment and maturation of MoMFs during liver injury^16^. We thereafter designed four CCR8 antagonizing peptides based on the analysis of CCR8 binding ligands and CCR8 active site. Based on the previous studies, non-peptide antagonists share common pharmacophore containing central positive nitrogen that interacts with the glutamic acid (Glu286) at the CCR8 active site^45^^,^^46^. Hence, we designed AP8i, AP8ii and AP8iv peptides with an Arg, while we replaced Arg with a Lys in AP8iii. Moreover, it is shown that the groups attached to the positive nitrogen interacts with the aliphatic and aromatic residues present in the active site of CCR8^46^ therefore we introduced Phe (F), Typ (W), Tyr (Y) residues next to the Arg/Lys residue to facilitate the high-affinity interaction between the peptide antagonists at the CCR8 active site.

The *in silico* docking and MD simulations studies highlighted the important interactions of AP8ii (among four AP8 peptides) and R243 with amino acid residues of CCR8, indicating their high affinity toward the protein compared to other designed peptides. Furthermore, AP8ii occupied the cavity more firmly than R243 in the Homology build CCR8 model. While R243 only displayed fewer and weak interactions with CCR8, AP8ii showed multiple and high-affinity interactions with CCR8. The docking score of peptides AP8ii was found to be −2.849, better than R243 with dock score −2.467, indicating better binding affinity of AP8ii. Similarly, AP8ii exhibited a better binding pose compared to R243 when docked at the homology-build CCR8 protein active site. Gadhe et al.^46^, observed Glu286, Lys195 and Tyr113 to be crucial for CCR8 antagonism. AP8ii displayed aromatic hydrogen bond and salt bridge interactions with Glu286, *π*–*π* interactions with Tyr113 and Phe254 and a pivotal hydrogen bonding with Glu177. In contrast, R243 displayed only hydrogen bonding interaction with Tyr172 and Tyr187 amino acid residues. Our findings further indicated that AP8ii maintained consistent interactions with these crucial amino acid residues, whereas R243 although accommodates in the active site, yet it did not exhibit the requisite interactions with the essential amino acid residues. Consequently, these outcomes underscore the superior antagonistic efficacy of AP8ii over R243. Further, our results were validated through different software's and MD simulations supporting better binding affinity of AP8ii at the active site of CCR8 compared to R243. We further confirmed the higher affinity of AP8ii over other peptides using *in vitro* binding studies where AP8ii showed distinctively higher cell interaction possibly *via* CCR8 as CCL1, R243 and anti-CCR8 antibody partially inhibited the interaction of AP8ii with CCR8 in a co-incubation study, more prominently in pre-incubation studies where AP8ii was added following CCL1, R243 or anti-CCR8 antibody. CCL1, R243 and CCR8 antibody only induced partial inhibition in AP8ii binding suggesting AP8ii interacts at different and more sites on CCR8 than CCL1, R243 and/or CCR8 antibody binding site. In a recent study, it has been demonstrated that CCL1 follows a two-step, two-site binding sequence to CCR8^52^. CCL1 engages the CCR8 orthosteric core through polar interactions with Tyr94, Asp97 and His283, and hydrophobic interactions with Tyr184. These interactions facilitate receptor activation whereby CCL1 triad S25-M26-Q27 interacts with CCR8 residues Glu286 and Gln91–Tyr172 and Gln27–Tyr114 and Tyr184 respectively^52^. Our peptide antagonizes with these interactions as supported by our docking and MD simulation studies. These results indicate that AP8ii possibly act as an orthosteric inhibitor inhibiting the interaction of CCL1 (and CCR8 ligands) and/or allosteric antagonist interacting at the different site and causing a conformational shift preventing the interaction of CCL1 however further studies are needed to validate this possibility.

To examine the therapeutic efficacy of AP8ii, we performed an *in vitro* CCL1-induced Transwell chemotaxis assay. Given the substantial homology (71%) between human and murine CCR8, both receptors respond to human and murine CCL1^13^. Human CCL1 induced significant Transwell chemotaxis of murine RAW macrophages as well as human THP-1 monocytes. Intriguingly, our AP8ii significantly inhibited the CCL1-induced chemotaxis of RAW macrophages and THP-1 monocytes. After successful inhibition *in vitro*, we investigated the recruitment of monocytes to the liver in an acute CCl_4_-induced *in vivo* mouse model. Upon recruitment facilitated by chemokine ligands, Ly6C^high^ monocytes infiltrate the liver and subsequently undergo maturation into pro-inflammatory macrophages^53^. To explore the inhibitory effects of AP8ii in an *in vivo* setting, it is essential to discern between CD11b^high^ F4/80^intermediate^ MoMFs and F4/80^high^ CD11b^low^ liver resident KCs^54^. We found, as expected, that the MoMF population was significantly decreased with AP8ii, further emphasizing the efficacy of AP8ii in inhibiting CCR8-dependent monocyte recruitment during liver inflammation. We finally investigated the effects of CCR8 inhibition on inflammation and fibrosis in our acute CCl_4_-induced *in vivo* mouse model. We observed that CCR8 antagonism resulted in decreased liver inflammation and early fibrosis as confirmed by downregulated inflammatory and fibrotic markers. Initially it has been thought that the antifibrotic effects of CCR8 inhibition are indirect stemming from the reduced infiltration of MoMFs and the subsequent decrease in inflammation^16^. However, more recent insights have put forth the suggestion that the CCL1/CCR8 axis might directly contribute to the process of fibrogenesis^30^^,^^55^. Liu et al.^55^, described that CCR8-expressing fibroblasts are recruited and activated by CCL1. The significant downregulation of collagen-I expression in AP8ii treated mice, is possibly not only caused by decreased infiltration of MoMFs and decreased inflammation, but also by decreased recruitment of fibroblasts. In addition to its role in fibroblast recruitment, CCR8 is implicated in the process of fibrogenesis through the *trans*-differentiation of macrophages into myofibroblasts, facilitated by the action of TGF*β*^30^. Consequently, inhibition of CCR8 on infiltrated macrophages, CCR8 could be a direct antifibrotic. However, the direct role of AP8ii on fibroblasts recruitment and on macrophages-myofibroblasts *trans*-differentiation warrants further investigation.

## 5 Conclusions

In conclusion, in this study, we report a design, molecular characterization and therapeutic investigation of a CCR8 antagonizing peptide AP8ii that inhibited monocyte-macrophage chemotaxis *in vitro* and ameliorated MoMFs infiltration, liver inflammation and early fibrosis *in vivo*. We further demonstrated the safety profile of AP8ii for possible clinical application for the treatment of pathologies that involves CCL1‒CCR8 axis.

## Author contributions

Eline Geervliet and Sahil Arora – Methodology, Investigation, Validation, Visualization, Data curation, Writing – original draft. Carlos Antonio de Albuquerque Pinheiro: Methodology, Investigation, Validation, Visualization, Data curation, Writing – review & editing. Leon WMM Terstappen, Julieta Paez and Raj Kumar: Resources, Data curation, Supervision, Writing – review & editing. Ruchi Bansal: Conceptualization, Methodology, Formal analysis, Resources, Data curation, Writing – review & editing, Visualization, Supervision, Project administration, Funding acquisition.

## Conflicts of interest

The authors have no conflicts of interest to declare.

## Appendix A Supplementary information

The following are the Supporting Information to this article.
