# Supplementary material for: Design, molecular characterization and therapeutic investigation of a novel CCR8 peptide antagonist that attenuates acute liver injury by inhibiting infiltration and activation of macrophages
Source: Acta Pharm Sin B. 2025 Feb 21;15(4):2114–33. doi: 10.1016/j.apsb.2025.02.018 (PMC12137978; doi:10.1016/j.apsb.2025.02.018)
Supplement: Multimedia component 1 [file mmc1.pdf]

# Supplementary Figures

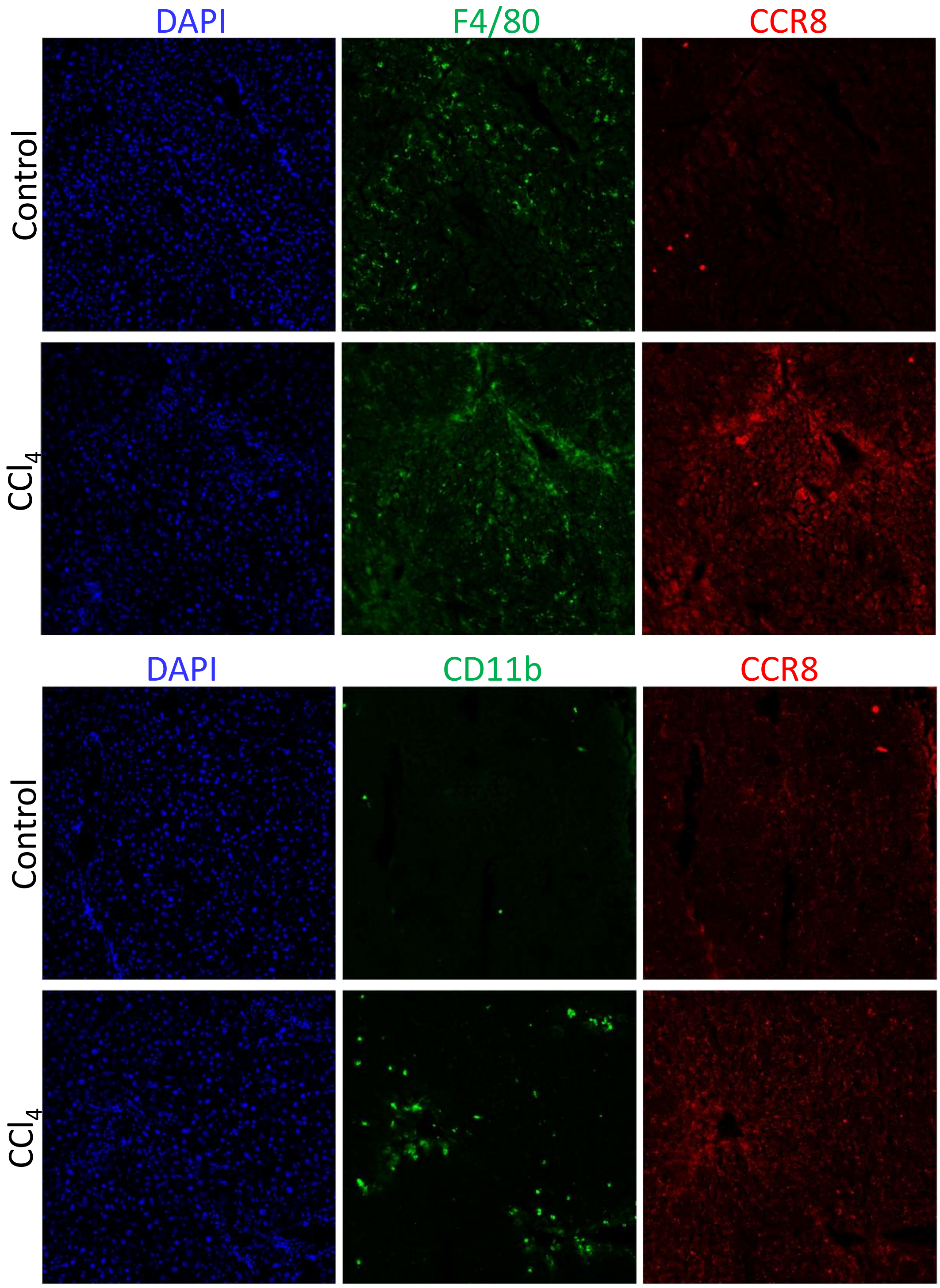


***Supplementary Figure S1.*** *Representative single-channel images (scale bar = 100 µm) of liver sections from healthy mice, n=10 and $CCl_4$ mice, n=12 stained with F4/80 or CD11b (green), CCR8 (red), and DAPI (nuclear staining, blue).*

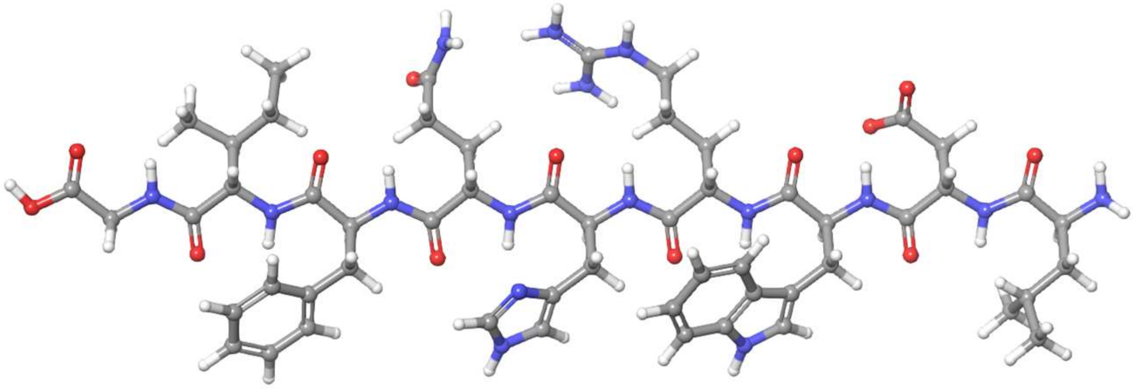

***Supplementary Figure S2. 3D structure of peptide AP8iii.***

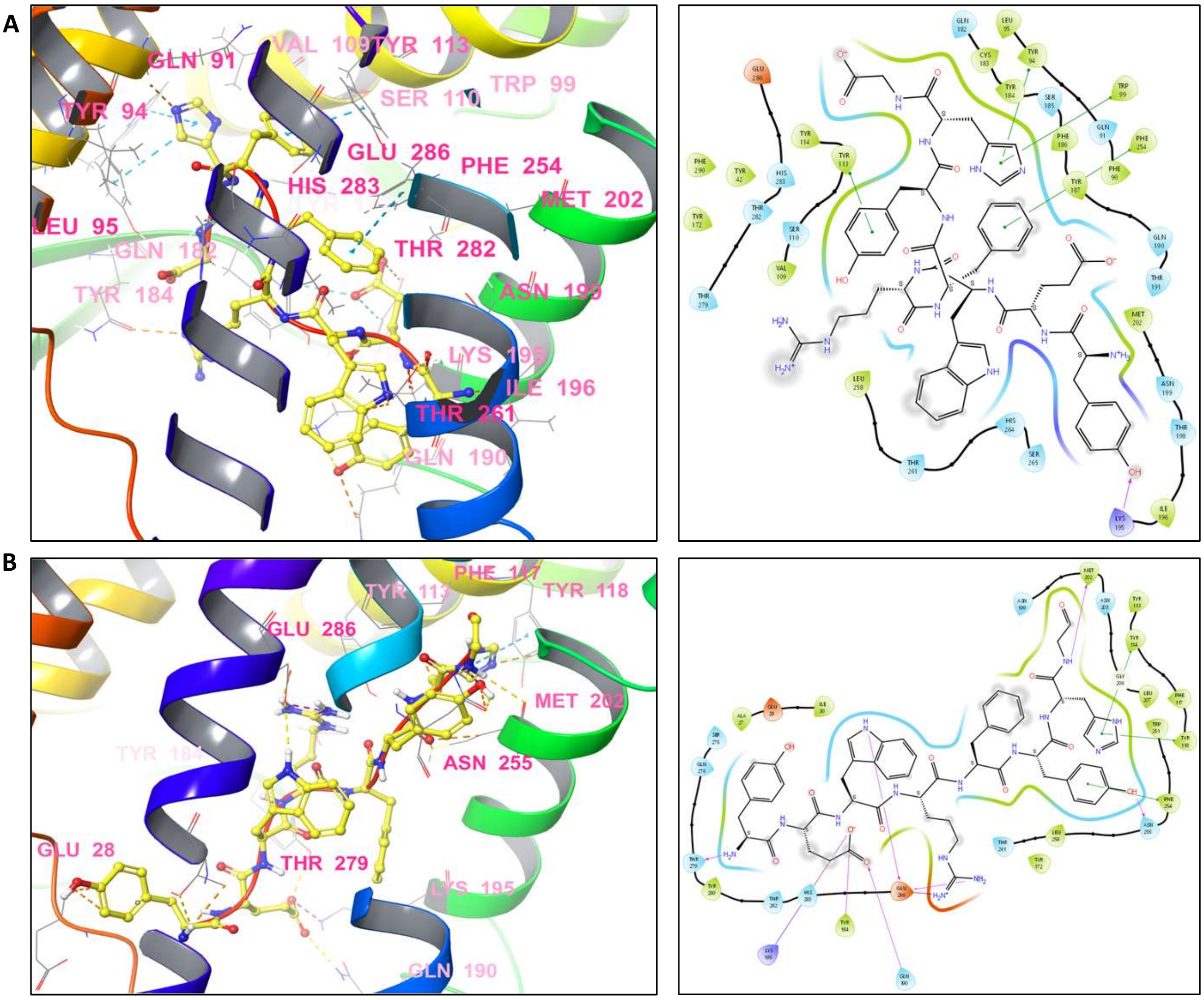


***Supplementary Figure S3. 3D (Left) and 2D (Right) docking poses of peptide AP8ii using different softwares*** *(A) Mdock PEP, and (B) Piper depicting binding sites of AP8ii at the CCR8 active site (homology build model).*

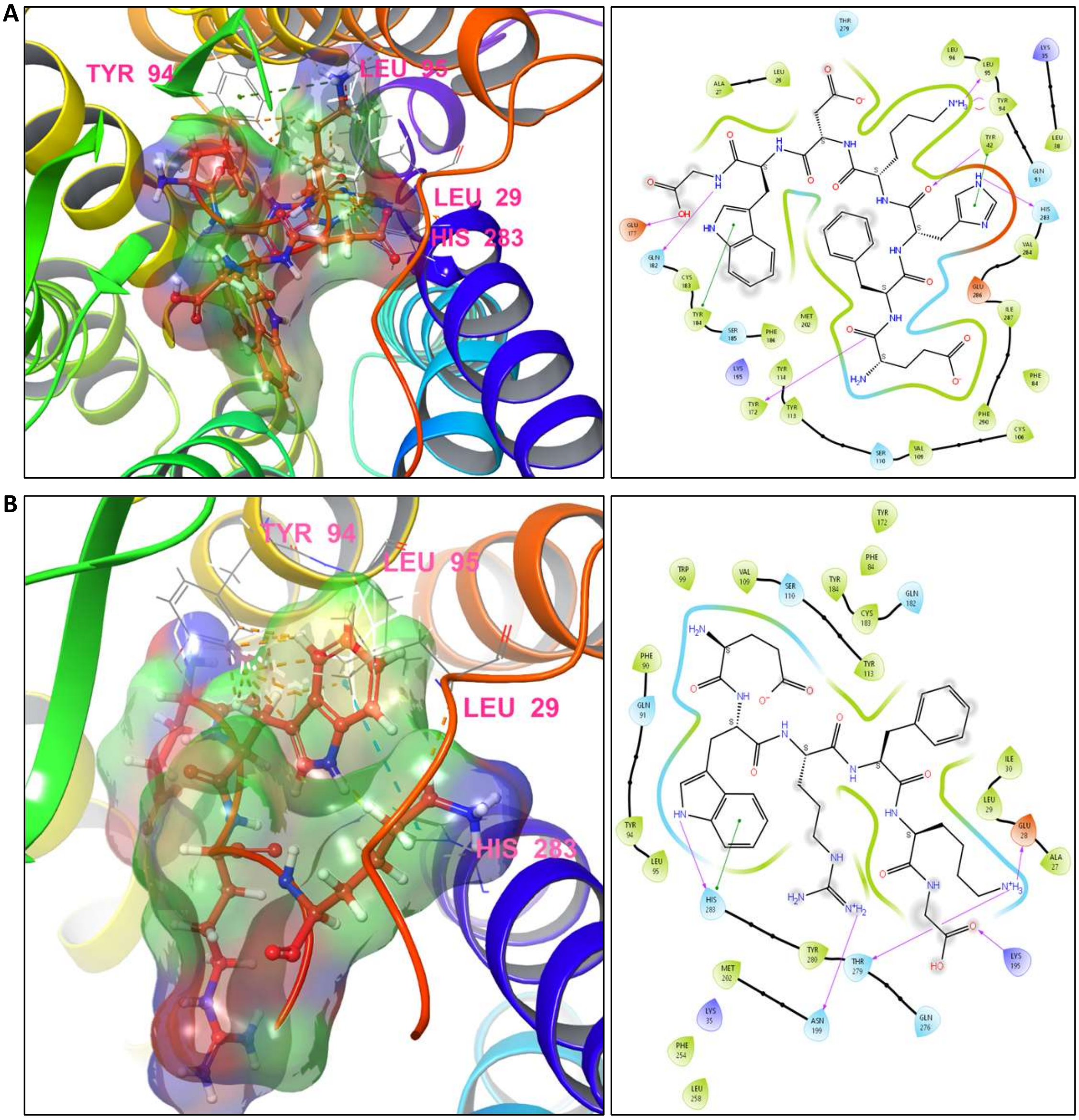


***Supplementary Figure S4. 3D (Left) and 2D (Right) docking poses of peptides A) AP8i and B) AP8iv at CCR8 active site (homology build model).***

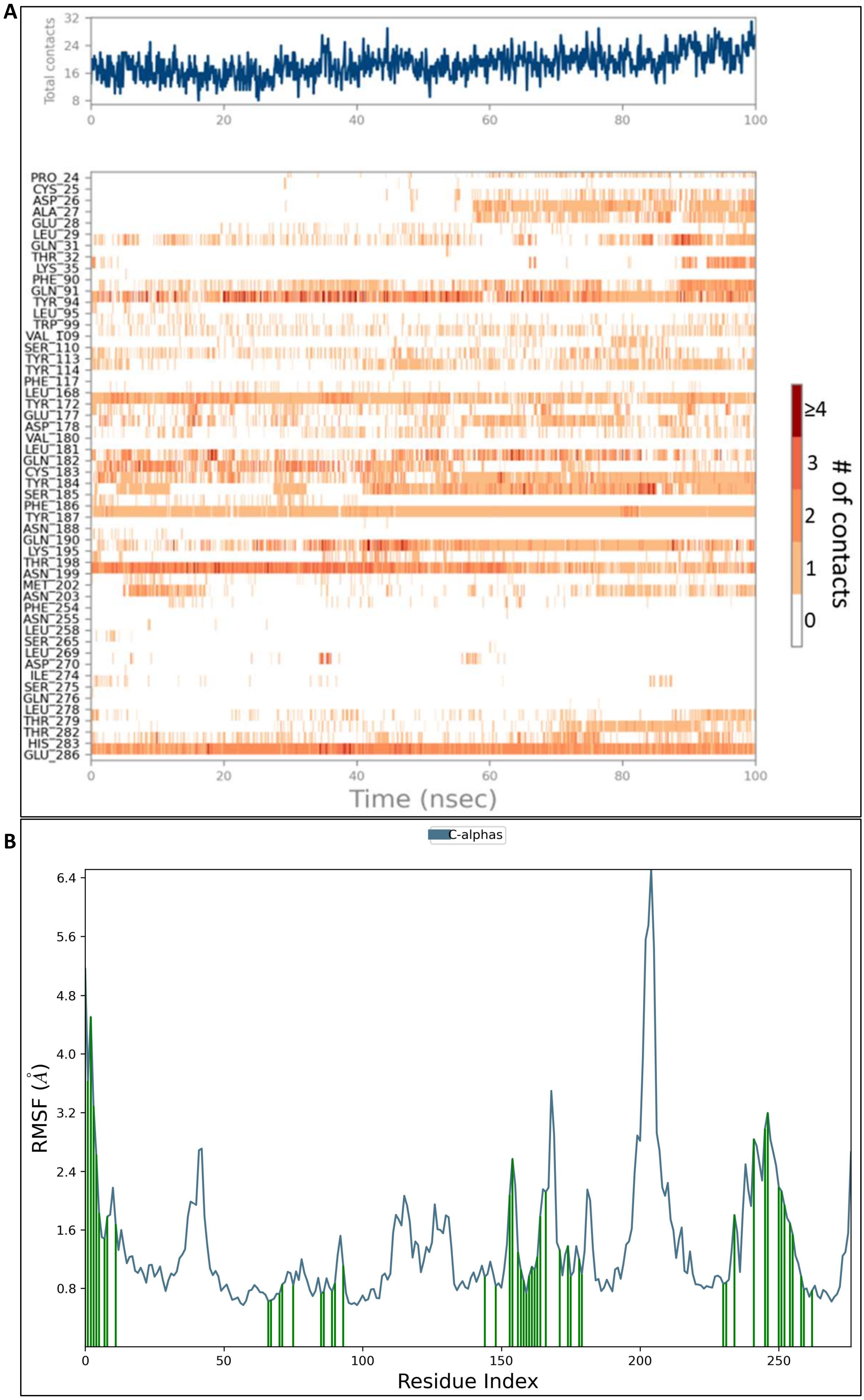


***Supplementary Figure S5. Protein-Ligand contacts of AP8ii with CCR8 in each trajectory frame.*** *A timeline representation of the interactions and contacts (H-bonds, Hydrophobic, Ionic, Water bridges) summarized in **Figure 4C**. **(A)** Plot showing the total number of specific contacts the protein makes with the ligand over the course of the trajectory. On the Y-axis, the residues are shown that interact with the ligand in each trajectory time frame (ns) shown on the X-axis. Some residues make more than one specific contact with the ligand, which is represented by a darker shade of orange, according to the scale shown to the right of the plot. **(B)** RMSF graph of protein CCR8 at 100 ns MD simulation. On this plot, peaks indicate areas of the protein that fluctuate the most during the simulation. Typically, the tails (N- and C-terminal) fluctuate more than any other part of the protein. Secondary structure like alpha helices and beta strands are usually more rigid than the unstructured part of the protein, and thus fluctuate less than the loop regions. Ligand (AP8ii) contacts with the protein residues that interact with the ligand are marked with green-coloured vertical bars.*

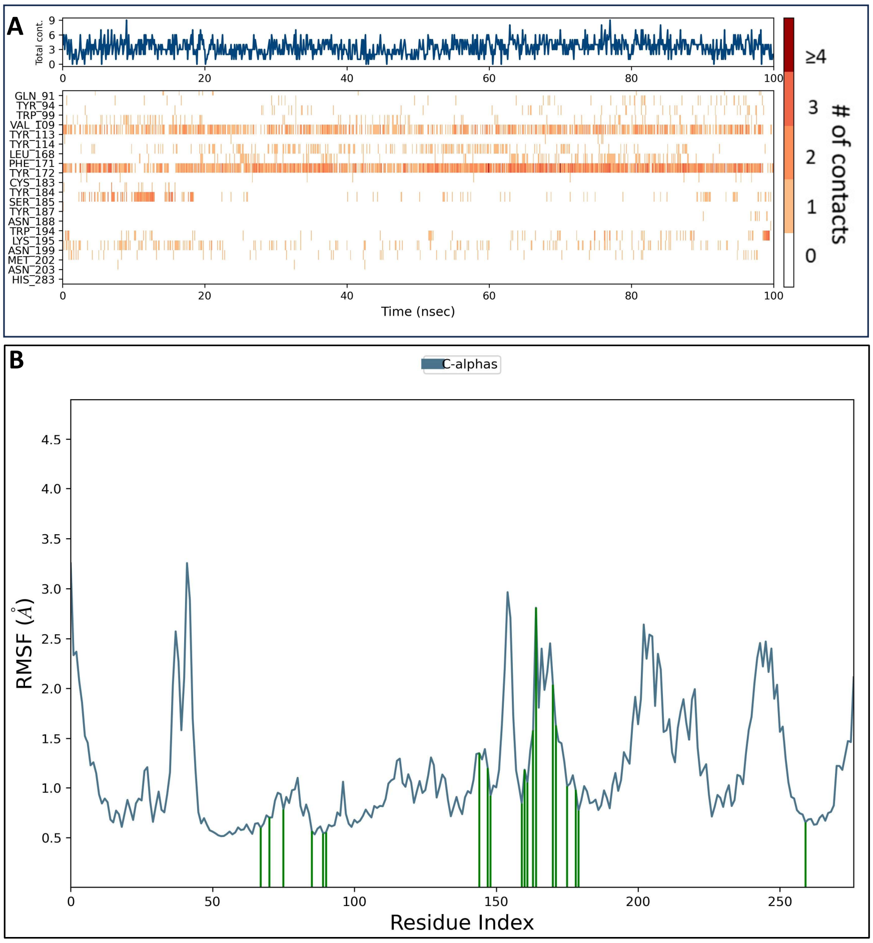


***Supplementary Figure S6. Protein-Ligand contacts of R243 with CCR8 in each trajectory frame.*** *A timeline representation of the interactions and contacts (H-bonds, Hydrophobic, Ionic, Water bridges) summarized in* ***Figure 5B****.* ***(A)*** *Plot showing the total number of specific contacts the protein makes with the ligand over the course of the trajectory. On the Y-axis, the residues are shown that interact with the ligand in each trajectory time frame (ns) shown on the X-axis. Some residues make more than one specific contact with the ligand, which is represented by a darker shade of orange, according to the scale shown to the right of the plot.* ***(B)*** *RMSF graph of protein CCR8 at 100 ns MD simulation. On this plot, peaks indicate areas of the protein that fluctuate the most during the simulation. Typically, the tails (N- and C-terminal) fluctuate more than any other part of the protein. Secondary structure like alpha helices and beta strands are usually more rigid than the unstructured part of the protein, and thus fluctuate less than the loop regions. Ligand (R243) contacts with the protein residues that interact with the ligand are marked with green-coloured vertical bars.*

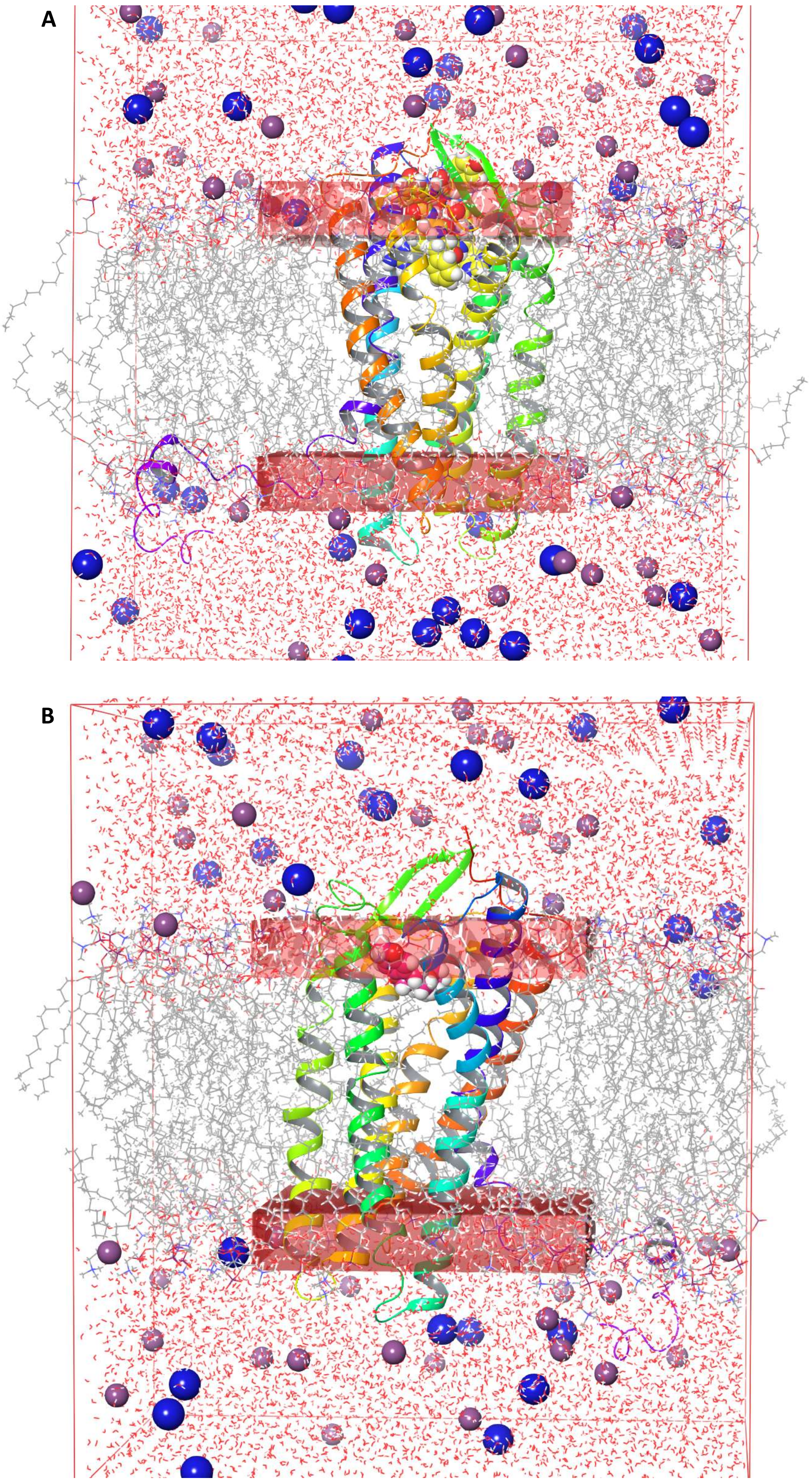


***Supplementary Figure S7. A. Snapshot of complex AP8ii-CCR8 (A) and R243-CCR8 (B).*** *CCR8 is represented by the helices and AP8ii or R243 is shown by space filling VDW style. DPPC lipids are represented by grey lines and SOL by red lines. Cl is represented by magenta, and Na by blue spheres.*

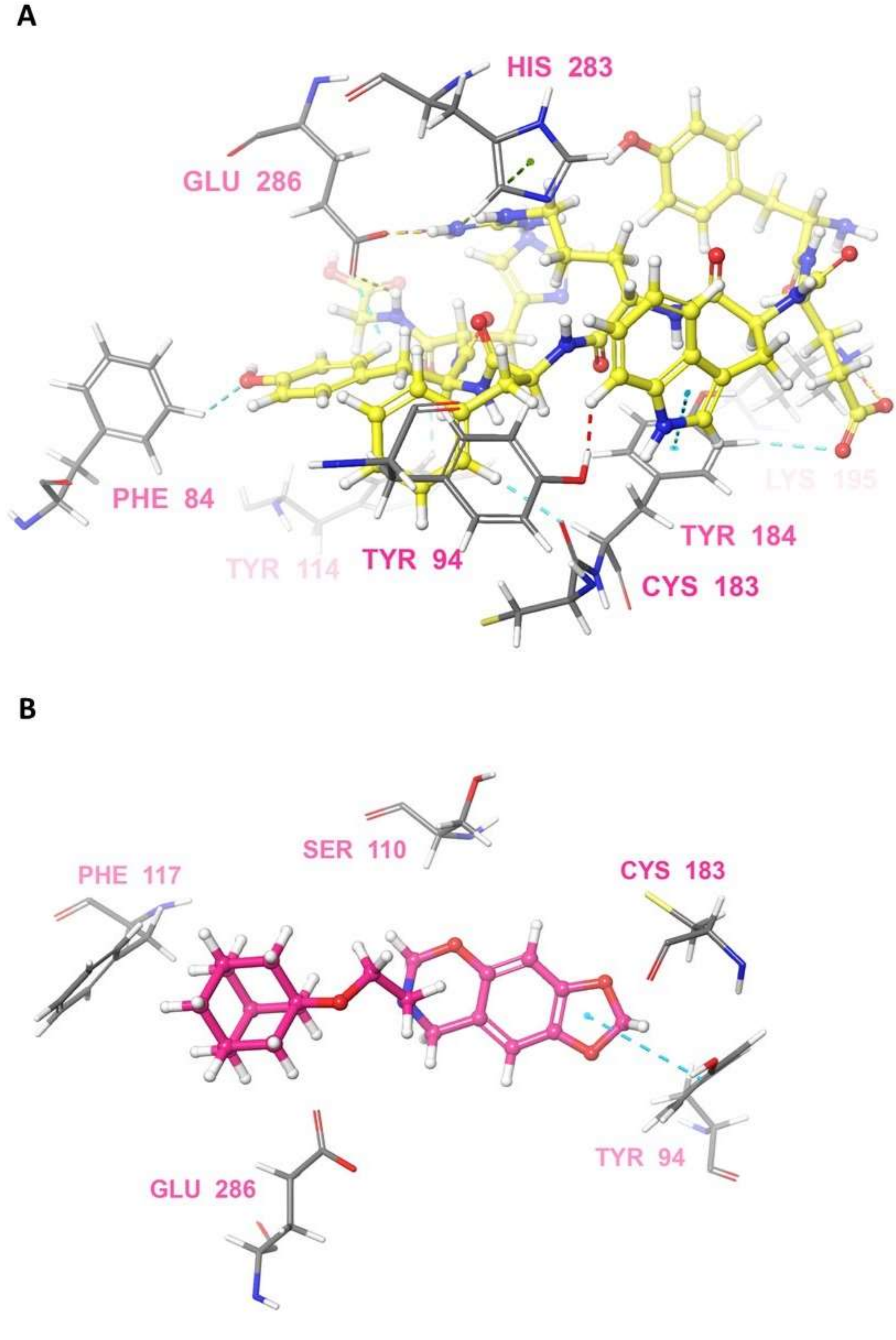


***Supplementary Figure S8.*** *Three-dimensional (3D) interaction analysis of (A) AP8ii and (B) R243 at the active site of mutated CCR8 protein where Tyr113 was replaced with Ala113.*

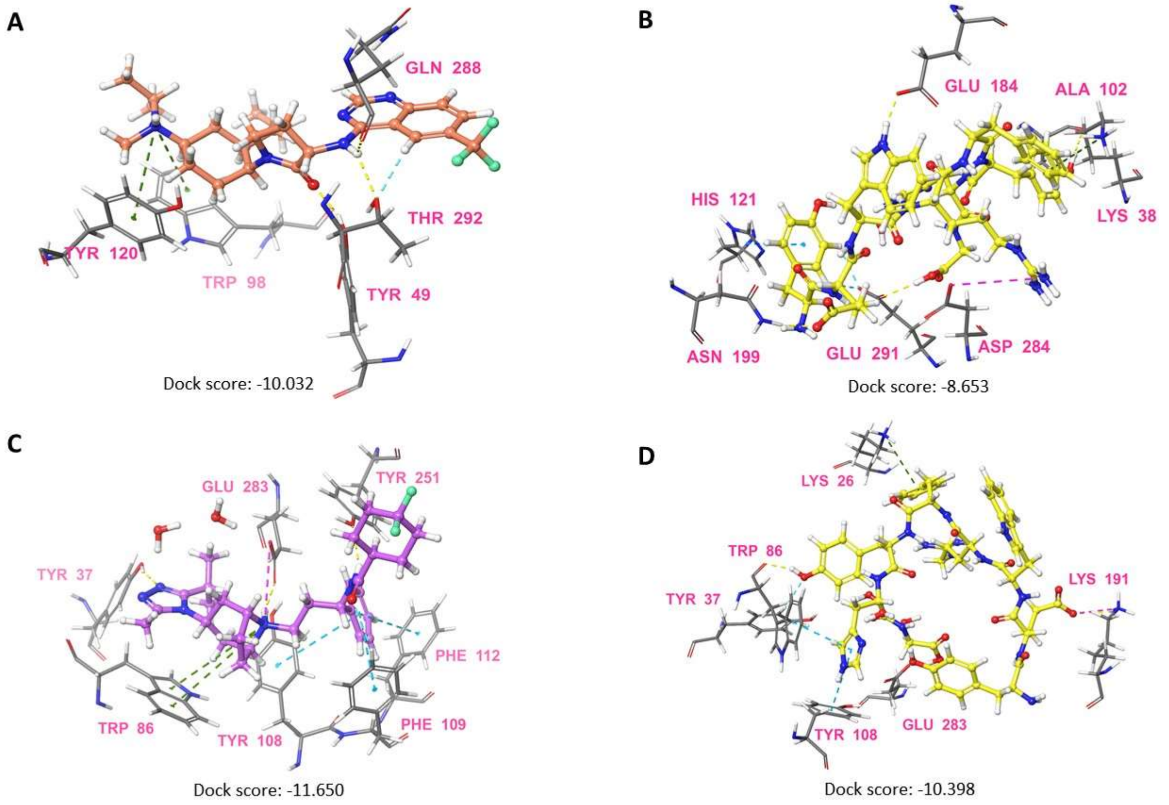


***Supplementary Figure S9.*** *Three-dimensional (3D) interaction analysis of A) co-crystallized ligand, B) AP8ii at the active site of CCR2 (PDB: 5T1A), C) co-crystallized ligand, and D) AP8ii at the active site of CCR5 (PDB: 4MBS).*

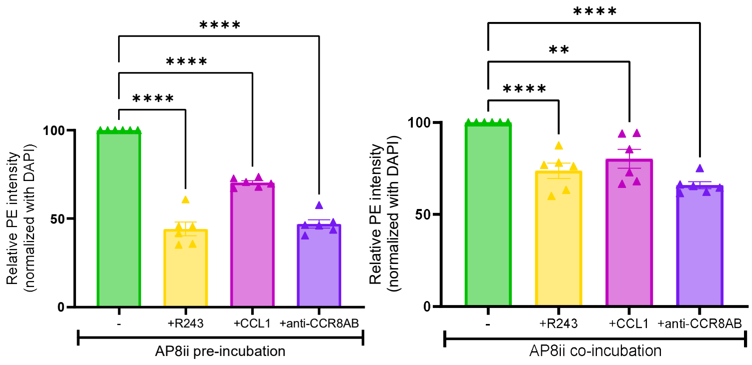


***Supplementary Figure S10. AP8ii-CCR8 interaction analysis.*** *Quantitative analysis of the images from co-incubation or pre-incubation studies (refer to Figure 7). RhB-labelled AP8ii (10 µM) that was either co-incubated with R243 (10 µM), CCL1 (100 ng/mL), or the anti-CCR8 antibody (5.0 µg/mL) for 4 h or R243 (10 µM), CCL1 (100 ng/mL), or the anti-CCR8 antibody (5.0 µg/mL) was preincubated for 2 h followed by incubation with AP8ii (10 µM) for 4 h. All results are presented as mean ± SEM from three independent experiments. **p < 0.01, ****p<0.0001, One-way ANOVA with Bonferroni post hoc test.*

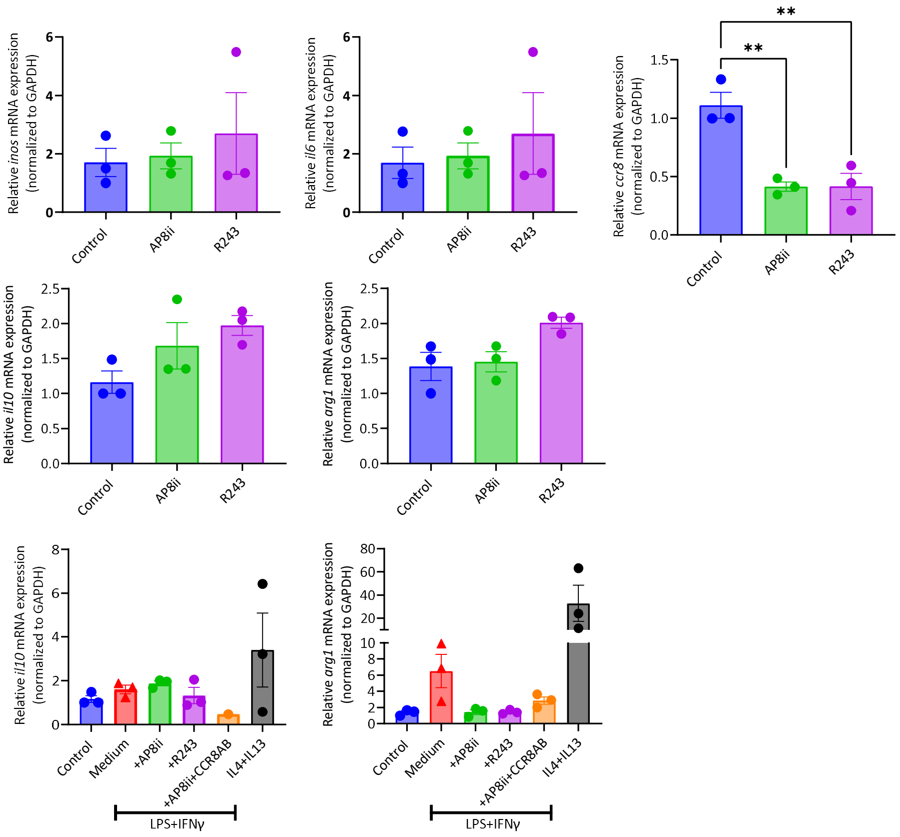


***Supplementary Figure S11. Effect of AP8ii and R243 on mouse RAW macrophages.*** *Graphs showing the gene expression analysis of inos, il6, ccr8, il10, and arg1 performed on mouse RAW 264.7 macrophages. The cells were incubated with and without 100 ng/mL LPS + 10 ng/mL IFNγ with and without 10 µM of CCR8 antagonist (R243) or CCR8 antagonizing peptide (AP8ii) with or without anti-CCR8 antibodies (CCR8AB) for 24 hours followed by gene expression analysis. All results are presented as mean ± SEM from three independent experiments. **p < 0.01, One-way ANOVA with Bonferroni post hoc test.*

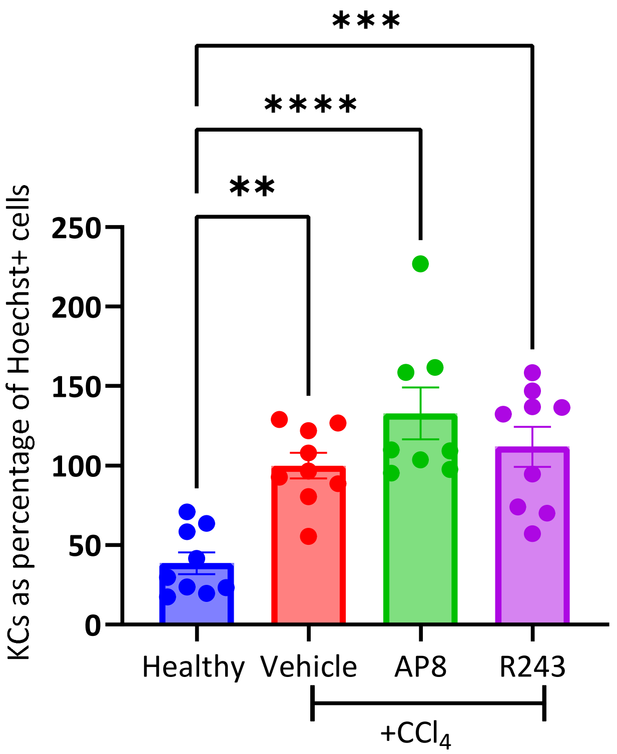


***Supplementary Figure S12: Quantitative flow cytometric analysis showing KCs as percentage of Hoechst+ cells***. *All results are presented as mean ± SEM. **p<0.01, ***p < 0.001, ****p<0.0001, One-way ANOVA with Bonferroni post hoc test.*

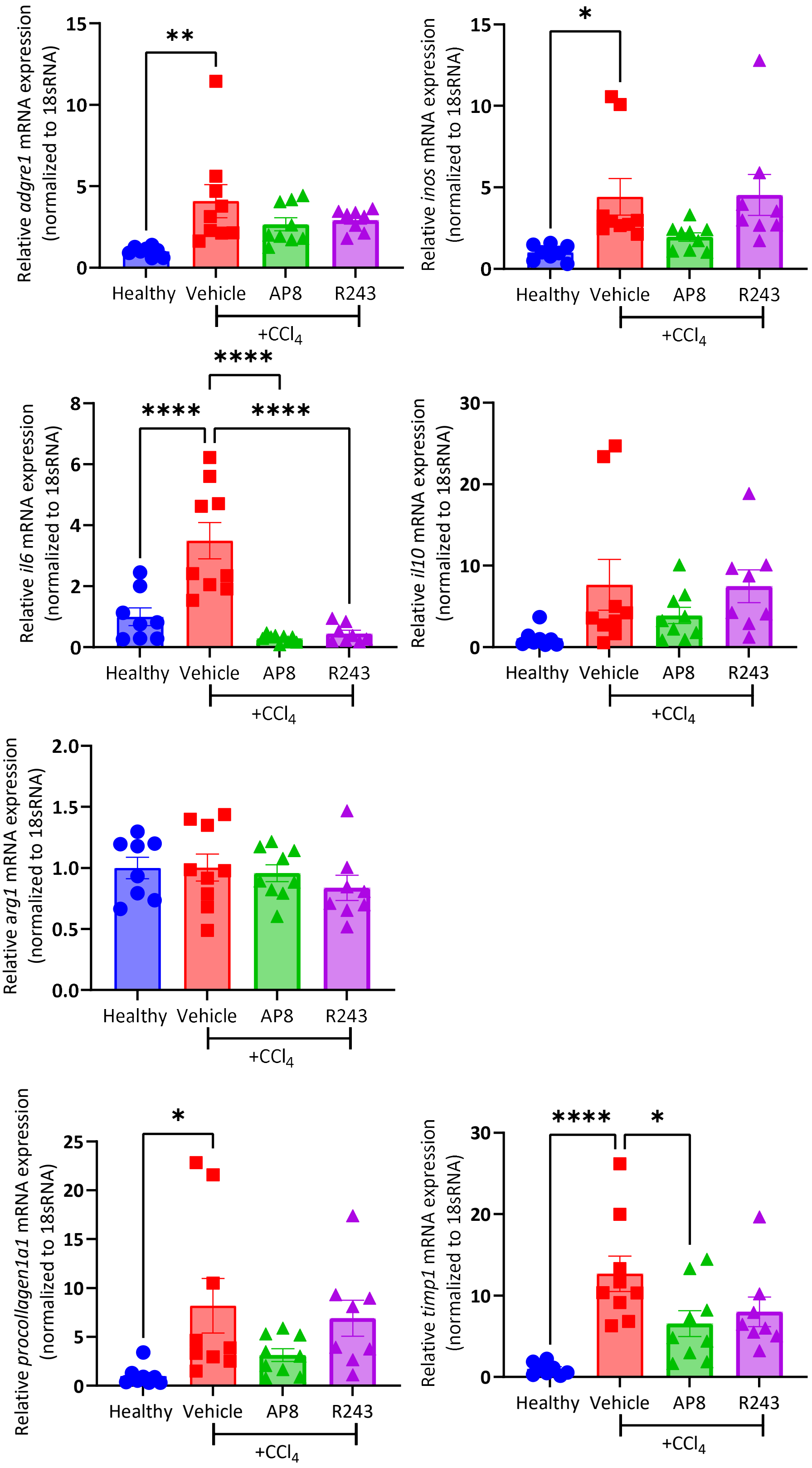


***Supplementary Figure S13. AP8ii ameliorates liver inflammation and early fibrosis during acute liver injury in vivo.*** *Relative gene expression of adgre1 (F4/80), inos (inducible nitric oxide synthase), il6 (interleukin-6), il10, arg1 (arginase 1), procollagen1a1 (collagen-I) and timp1 (tissue inhibitor of metalloproteinase I), normalized with 18s RNA) analysed in the liver (healthy, vehicle, AP8 and R243 mice, n=8-9 mice per group). All results are presented as mean ± SEM. *p<0.05, **p<0.01, ***p<0.001, ****p<0.0001, One-way ANOVA with Bonferroni post hoc test.*

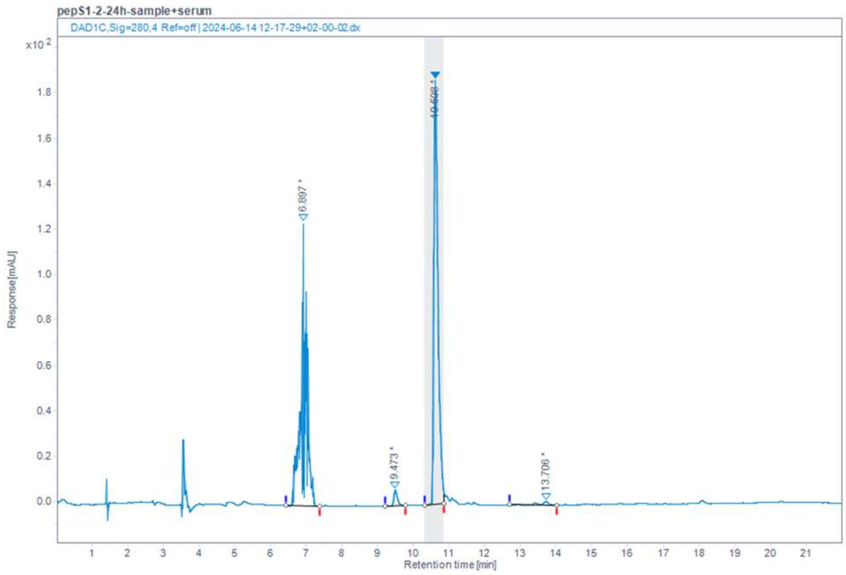


| Incubation time in serum (h) | Area (Mean ± SD) (a.u)<br><br>At rt ~ 10.6 min |
|---|---|
| 0 | 1726 ± 46 |
| 1 | 1800 ±27 |
| 2 | 1792 ± 40 |
| 4 | 1751 ± 12 |
| ... | |
| 24 | 1428 ± 105 |

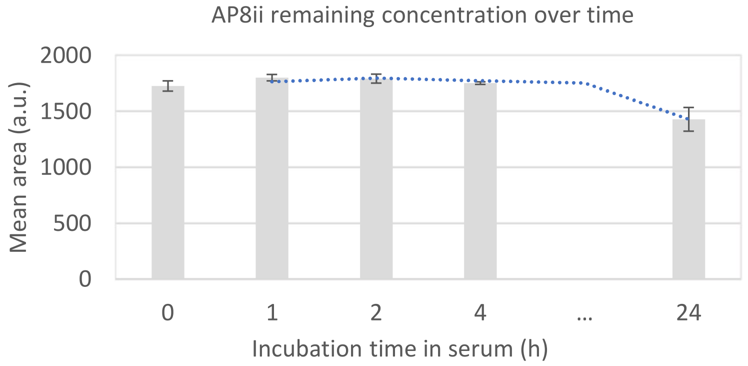


**Supplementary Figure S14.** ***Stability studies of AP8ii peptide upon increasing incubation time in 10 % v/v human serum and 37° C, as measured by analytic HPLC ($\lambda$= 280 nm used for detection).*** *Chromatogram trace (top) of the peptide sample after 24 h of incubation, where the colored peak at retention time (rt) ~ 10.6 min corresponds to the intact peptide. The other peaks at rt = 7, 9 and 13 min are assigned to the serum components. Quantification plot (bottom) shows high stability of peptide within the first 4 h, and >82% of peptide remains intact after 24 h.*

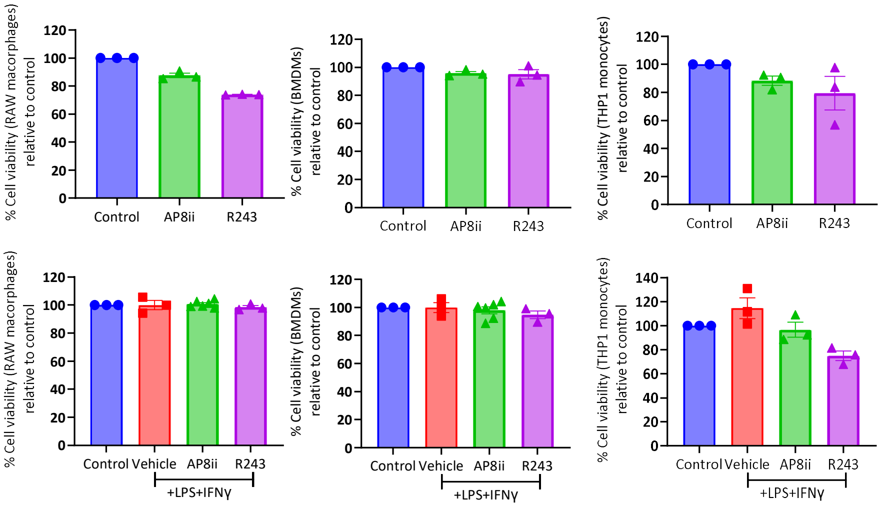


***Supplementary Figure S15. Effect of AP8ii and R243 on the cell viability of mouse and human macrophages.*** *Graphs showing the results of Alamar Blue cell viability assays performed on (A) mouse RAW 264.7 macrophages; (B) mouse primary bone marrow derived macrophages (BMDMs); and (C) human THP-1 monocytes. The cells were incubated with and without 100 ng/mL LPS + 10 ng/mL IFNγ with and without 10 µM of CCR8 antagonizing peptide (AP8ii) or CCR8 antagonist (R243) for 24 hours followed by Alamar blue assay. All results are presented as mean ± SEM from three independent experiments. The differences are not significant when compared with respective vehicle group.*

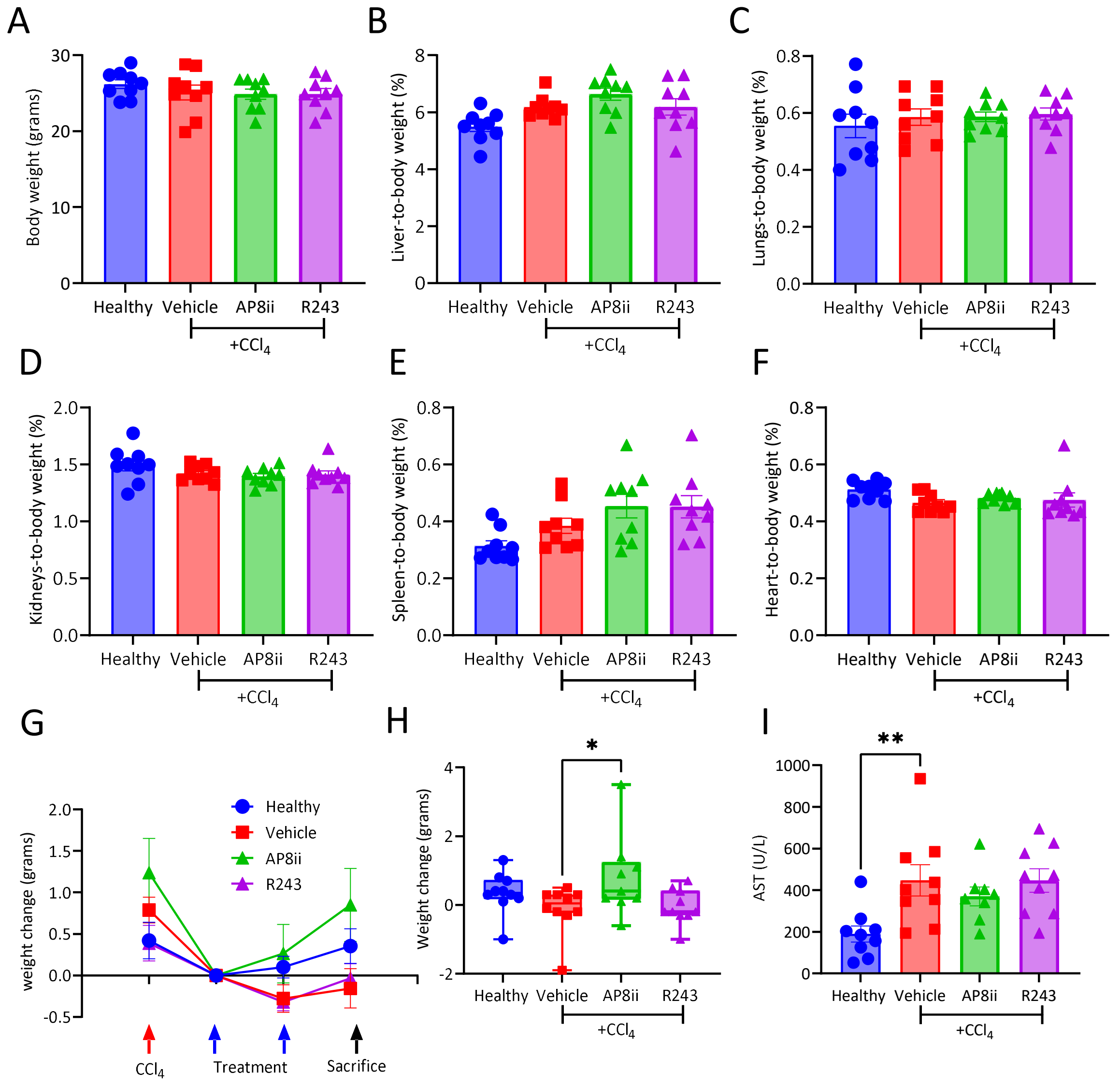


***Supplementary Figure S16. Safety profile of AP8ii.*** *(A-F) Body weight and organ to body weight ratio (healthy, vehicle, AP8 and R243 mice, n=9 mice per group) (G-H) Body weight change during treatment (I) AST levels. All results are presented as mean ± SEM. *p<0.05, One-way ANOVA with Bonferroni post hoc test.*
