# Supplementary material for: Design, molecular characterization and therapeutic investigation of a novel CCR8 peptide antagonist that attenuates acute liver injury by inhibiting infiltration and activation of macrophages
Source: Acta Pharm Sin B. 2025 Feb 21;15(4):2114–33. doi: 10.1016/j.apsb.2025.02.018 (PMC12137978; doi:10.1016/j.apsb.2025.02.018)
Supplement: Multimedia component 2 [file mmc2.pdf]

Supplementary experiments and data – HPLC analysis

<u>Control 1 – AP8ii Peptide only</u>

Peptide and MilliQ (no serum)

The peak at rt~ 10.6 min is assigned to the pure peptide, and a wavelength = 280 nm was chosen for detection (see UV-vis profile corresponding to the peptide).

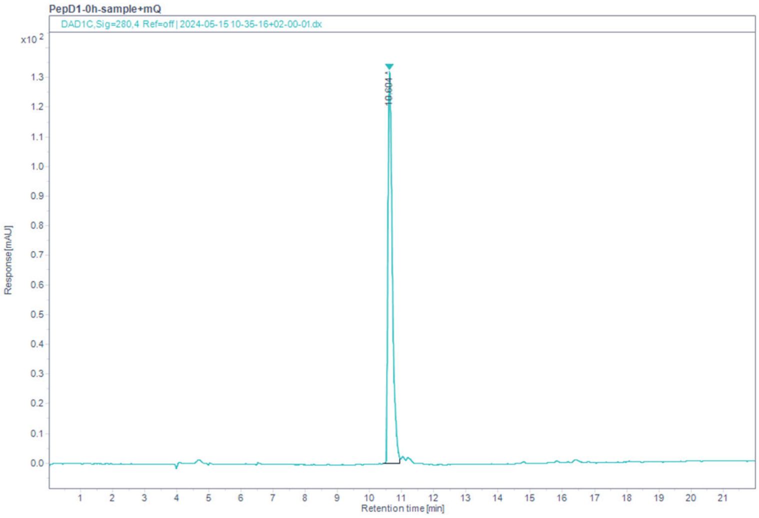

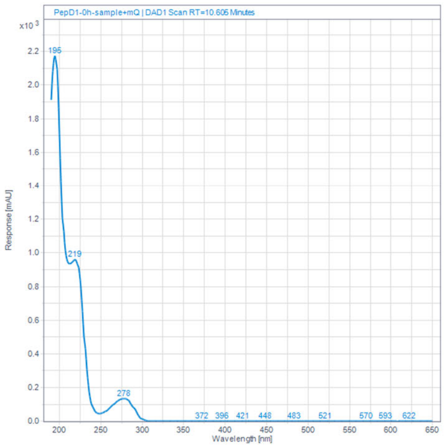

Signal description	RT (min)	Area (mAU·s)	Height (mAU)	Start time (min)	End time (min)
AP8ii	10,604	1263,752	132,091	10,406	10,945

<u>Control 2 – Human Serum only</u>

Serum and MilliQ water (no peptide, different injection preparations)

The peaks at rt ~ 7, 9 and 13 min are assigned to remaining serum components in the solution. Surprisingly, the relative intensity of those peaks varied as a function of the pipetting speed (slow vs fast) and the absence of the centrifugation step.

Stacked graph of different treatment of serum samples

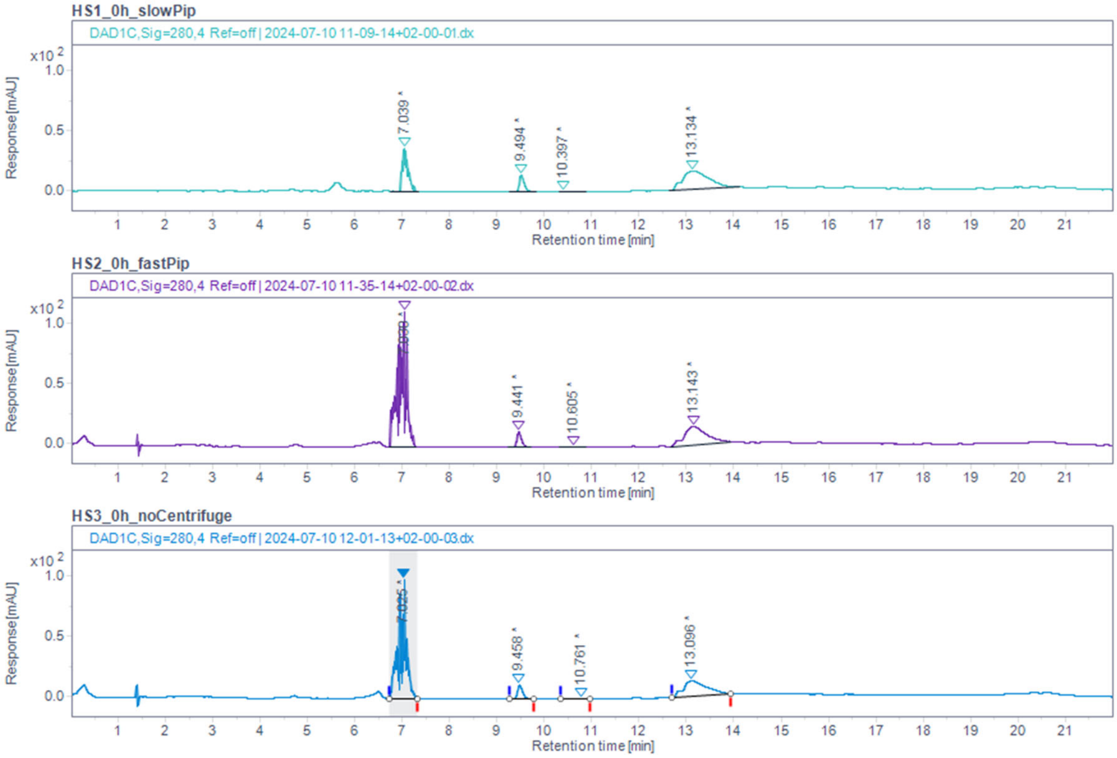

Raw data: Slow pipetting

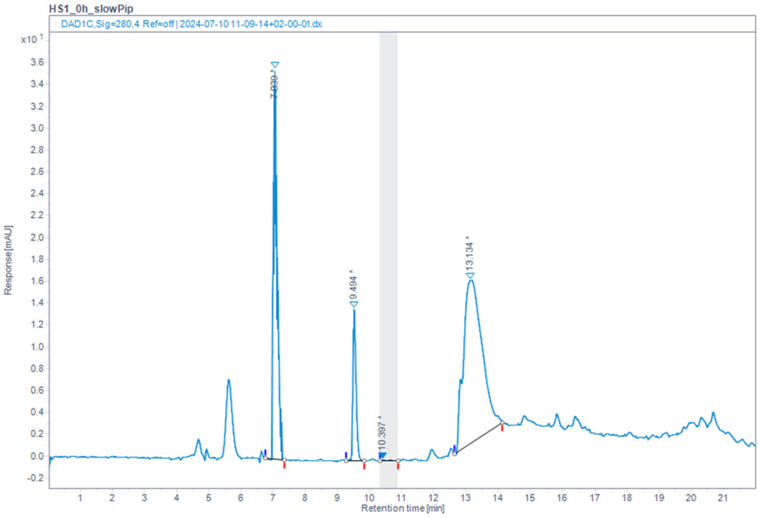

Signal description	RT (min)	Area (mAU·s)	Height (mAU)	Start time (min)	End time (min)
S1	7,039	282,59	35,674	6,733	7,336
S2	9,494	90,434	13,863	9,257	9,815
AP8ii	10,397	0,721	0,063	10,31	10,883
S3	13,134	606,716	14,976	12,642	14,135

Fast pipetting

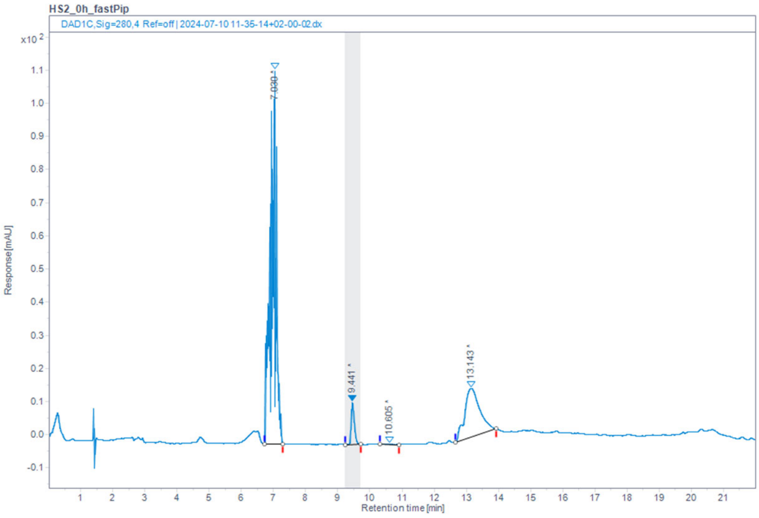

Signal description	RT (min)	Area (mAU·s)	Height (mAU)	Start time (min)	End time (min)
S1	7,03	1119,296	112,721	6,708	7,286
S2	9,441	83,948	12,64	9,242	9,709
AP8ii	10,605	1,343	0,103	10,32	10,892
S3	13,143	484,416	14,832	12,663	13,924

Without centrifugation step

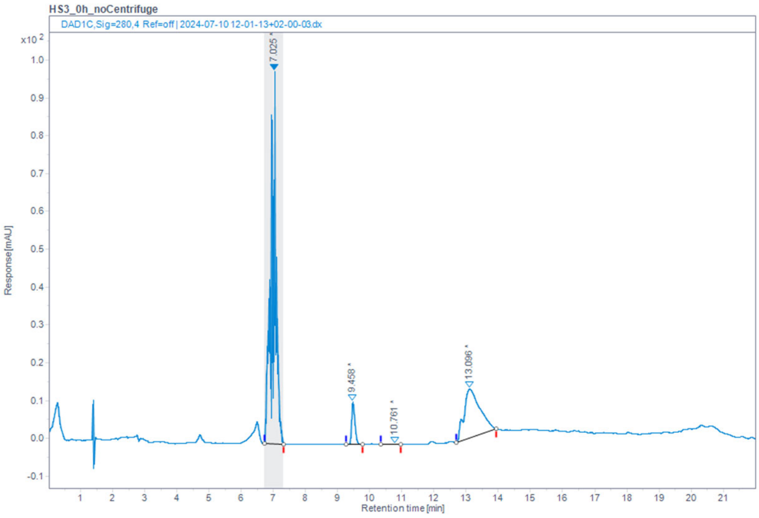

Signal description	RT (min)	Area (mAU·s)	Height (mAU)	Start time (min)	End time (min)
S1	7,025	908,301	98,516	6,719	7,323
S2	9,458	78,223	11,261	9,247	9,775
AP8ii	10,761	0,019	0,048	10,334	10,949
S3	13,096	447,906	13,007	12,697	13,935

Degradation study of AP8ii peptide in human serum

Representative raw data sets are included for each time point, and the UV-Vis spectrum showed corresponds to the peptide peak, rt ~10.6 min.

t = 0 h

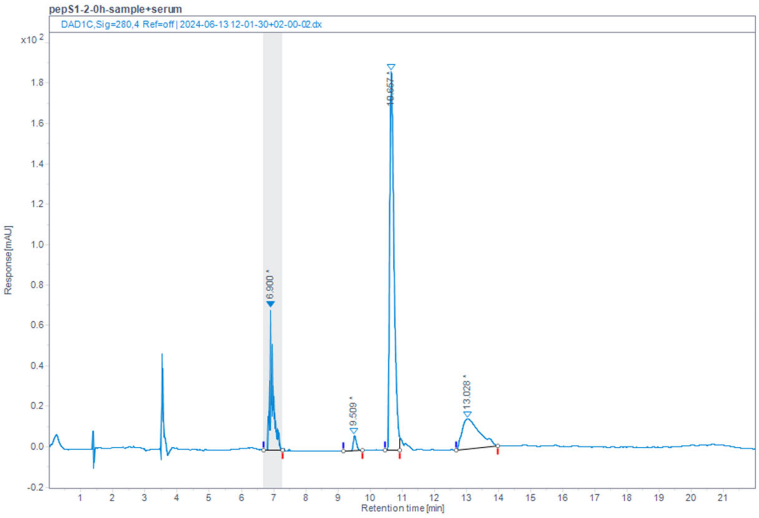

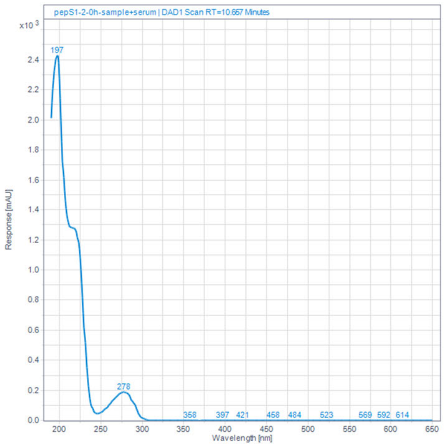

Signal description	RT (min)	Area (mAU·s)	Height (mAU)	Start time (min)	End time (min)
S1	6,9	404,439	69,922	6,678	7,273
S2	9,509	50,533	7,48	9,184	9,764
AP8ii	10,657	1759,329	187,195	10,482	10,936
S3	13,028	550,823	14,733	12,684	13,987

t = 1 h

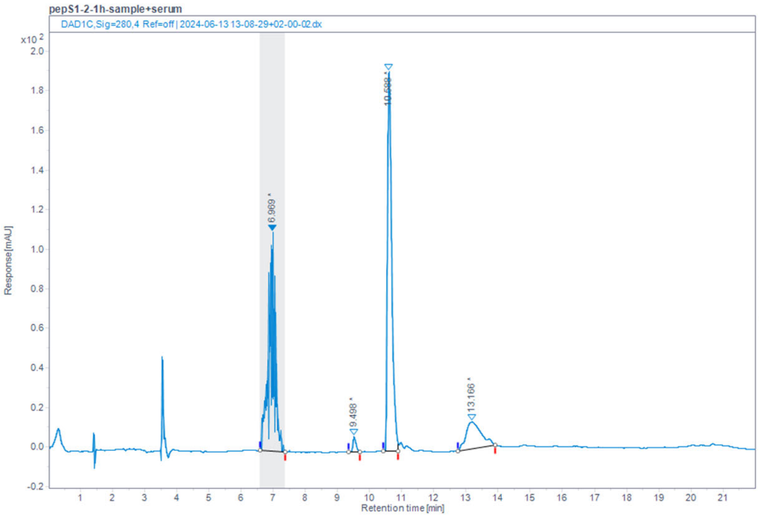

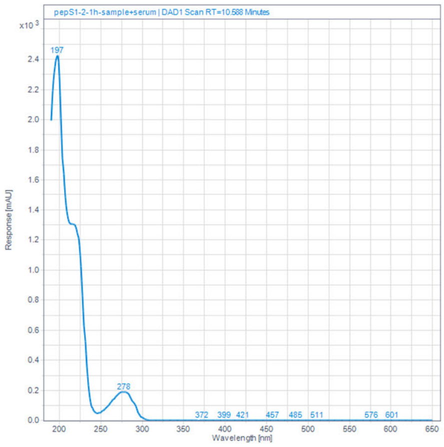

Signal description	RT (min)	Area (mAU·s)	Height (mAU)	Start time (min)	End time (min)
S1	6,969	1315,978	110,863	6,575	7,377
S2	9,498	50,036	7,715	9,327	9,681
AP8ii	10,588	1781,064	191,569	10,431	10,877
S3	13,166	455,58	13,614	12,738	13,906

t = 2 h

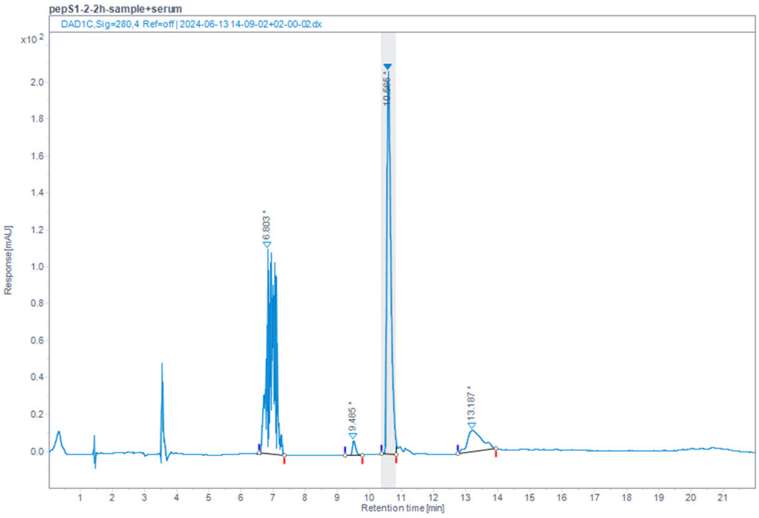

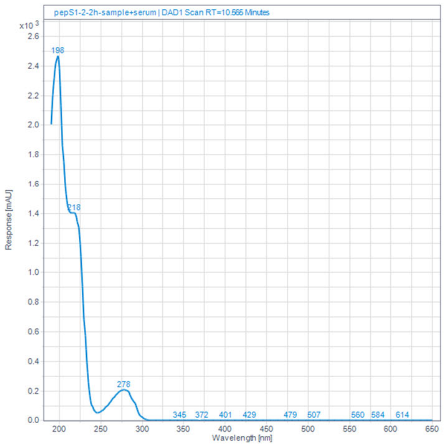

Signal description	RT (min)	Area (mAU·s)	Height (mAU)	Start time (min)	End time (min)
S1	6,803	1550,454	110,813	6,549	7,325
S2	9,485	53,418	7,937	9,232	9,772
AP8ii	10,565	1820,286	207,218	10,353	10,831
S3	13,187	368,102	11,551	12,736	13,925

t = 4 h

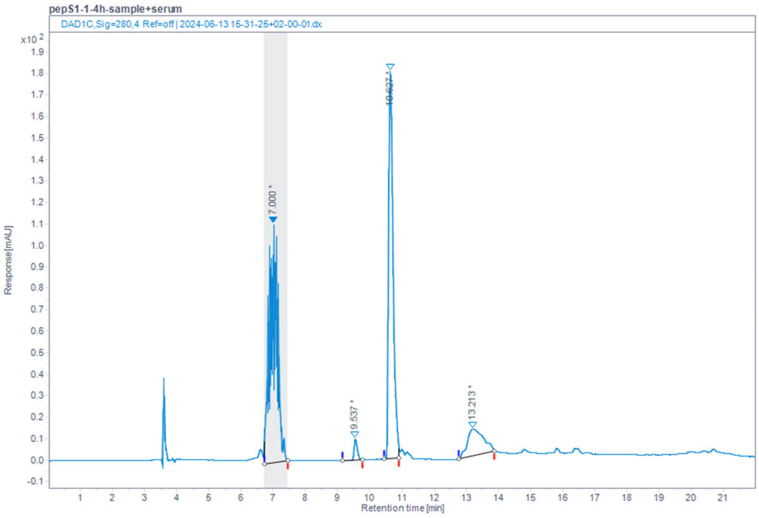

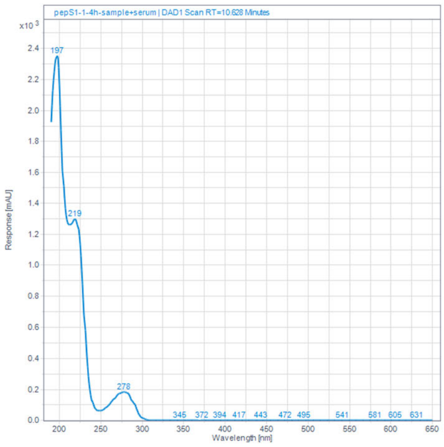

Signal description	RT (min)	Area (mAU·s)	Height (mAU)	Start time (min)	End time (min)
S1	7	1598,228	111,08	6,704	7,454
S2	9,537	64,475	9,775	9,161	9,777
AP8ii	10,627	1759,858	180,095	10,457	10,898
S3	13,213	438,071	12,46	12,773	13,889

t = 24 h

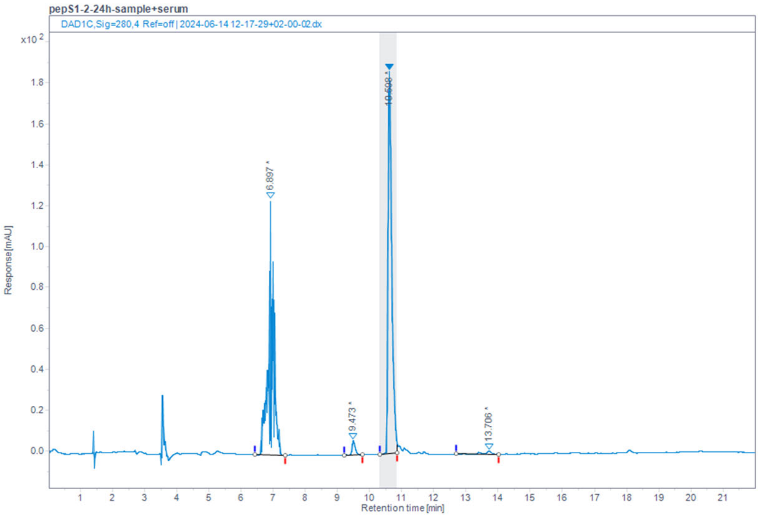

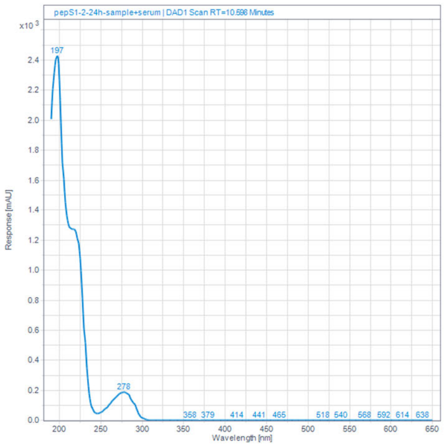

Signal description	RT (min)	Area (mAU·s)	Height (mAU)	Start time (min)	End time (min)
S1	6,897	1055,475	124,874	6,42	7,377
S2	9,473	40,825	6,794	9,199	9,765
AP8ii	10,598	1502,277	186,599	10,302	10,844
S3	13,706	16,376	1,628	12,699	14,011
