# Supplementary material for: Design, molecular characterization and therapeutic investigation of a novel CCR8 peptide antagonist that attenuates acute liver injury by inhibiting infiltration and activation of macrophages
Source: Acta Pharm Sin B. 2025 Feb 21;15(4):2114–33. doi: 10.1016/j.apsb.2025.02.018 (PMC12137978; doi:10.1016/j.apsb.2025.02.018)
Supplement: Multimedia component 3 [file mmc3.pdf]

PEPTIDEWEB.COM
Phone: +48 783 522 366
E-mail: info@peptideweb.com
Website: www.peptideweb.com
Bysewska 26b
80-298 Gdansk
Poland

21-01-2020

Sequence: LDWRHQFIG - AP8i

Average mass: 1171.324 Da
Monoisotopic mass: 1170.593 Da

Positive scan
m/z: 100-1250

z	Calculated m/z	Measured m/z
1	1171.60	1171.64
2	586.30	586.62
3	391.21	391.49

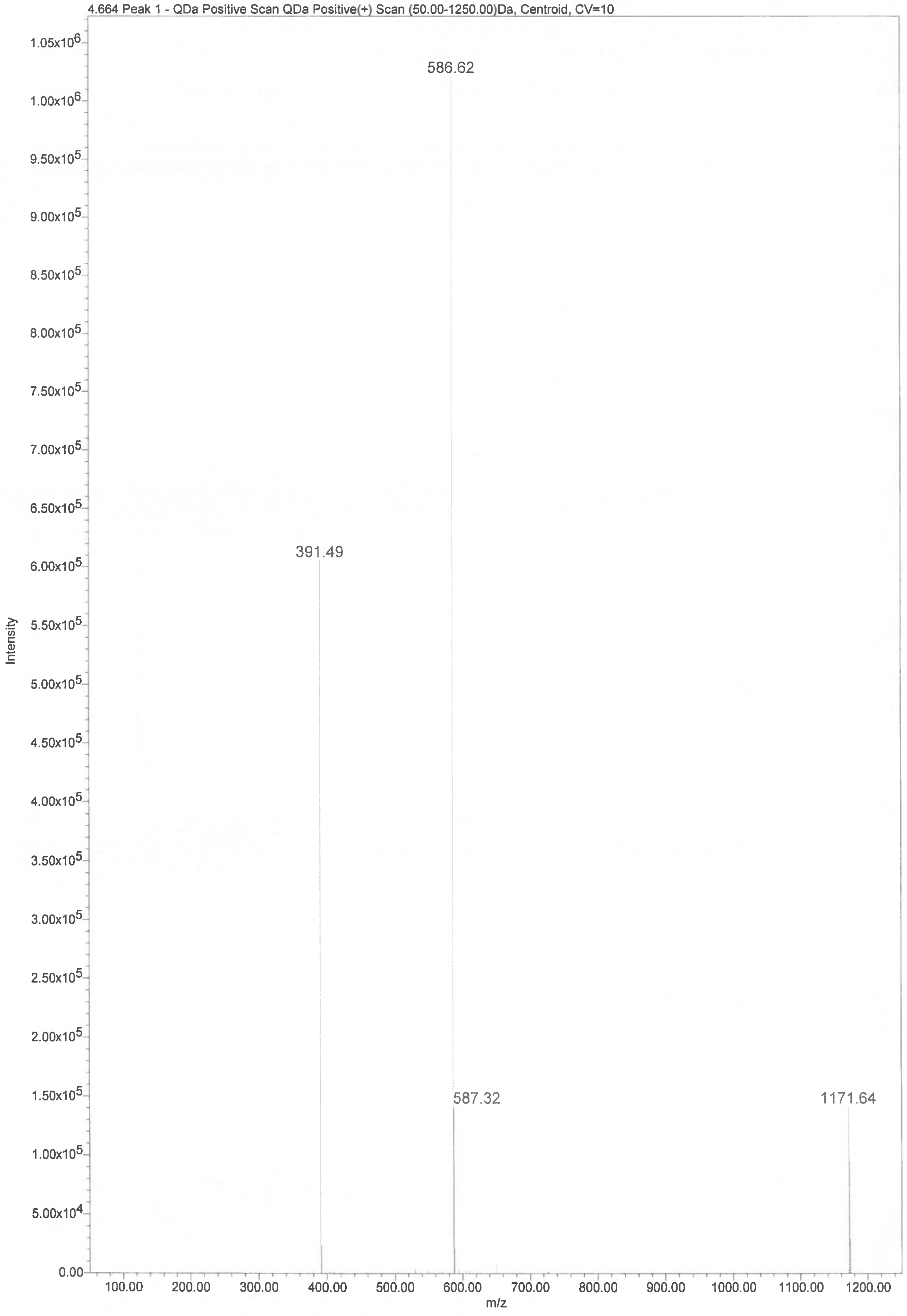
4.664 Peak 1 - QDa Positive Scan QDa Positive(+) Scan (50.00-1250.00)Da, Centroid, CV=10
586.62
391.49
587.32
1171.64
Intensity
m/z

Phone: +48 783 522 366
E-mail: info@peptideweb.com
Website: www.peptideweb.com

Bysewska 26b
80-298 Gdansk
Poland

Chromatographic analysis

RP-HPLC

Column:	Phenomenex, Luna C18(2), 5 µm, 100 Å, 4,6x150 mm
Mobile phase:	A – water, B – acetonitrile; both containing 0.1% TFA, v/v
Gradient:	10% -100%, 10 minutes
Flow:	2 ml/min
Detection:	UV, 214 nm

Chromatogram : LDWRHQFIG, 10-100%, 2ml, 214nm, 10 min58_channel1

System : HPLC
Method : 10-100%, 2ml, 214nm, 10 min
User : User1

Acquired : 2020-01-08 15:35:27
Processed : 2020-01-19 14:59:31
Printed : 2020-01-19 14:59:35

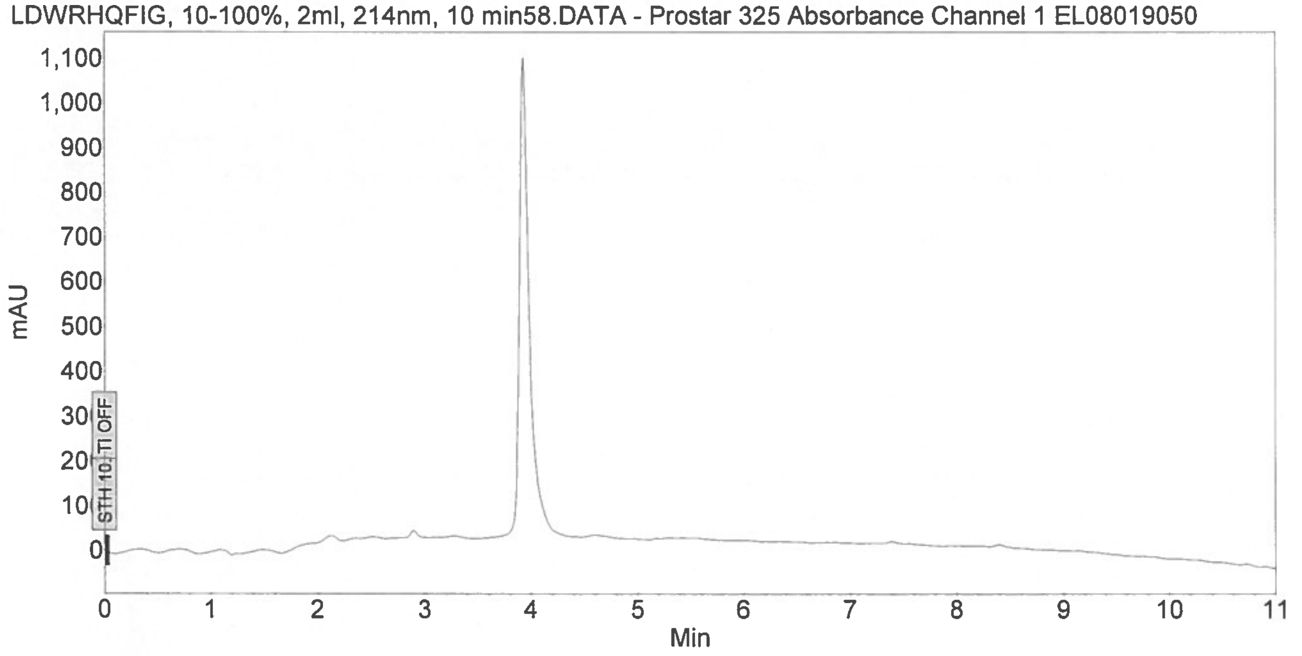

Peak results :

Index	Name	Time [Min]	Quantity [% Area]	Height [mAU]	Area [mAU.Min]	Area % [%]
Total			0.00	0.0	0.0	0.000

PEPTIDEWEB.COM
Phone: +48 783 522 366
E-mail: info@peptideweb.com
Website: www.peptideweb.com
Bysewska 26b
80-298 Gdansk
Poland

21-01-2020

Sequence: YEWRFYHG – AP8ii

Average mass: 1157.253 Da
Monoisotopic mass: 1156.509 Da

Positive scan
m/z: 100-1250

z	Calculated m/z	Measured m/z
1	1157.52	1157.61
2	579.26	579.63
3	386.51	386.84

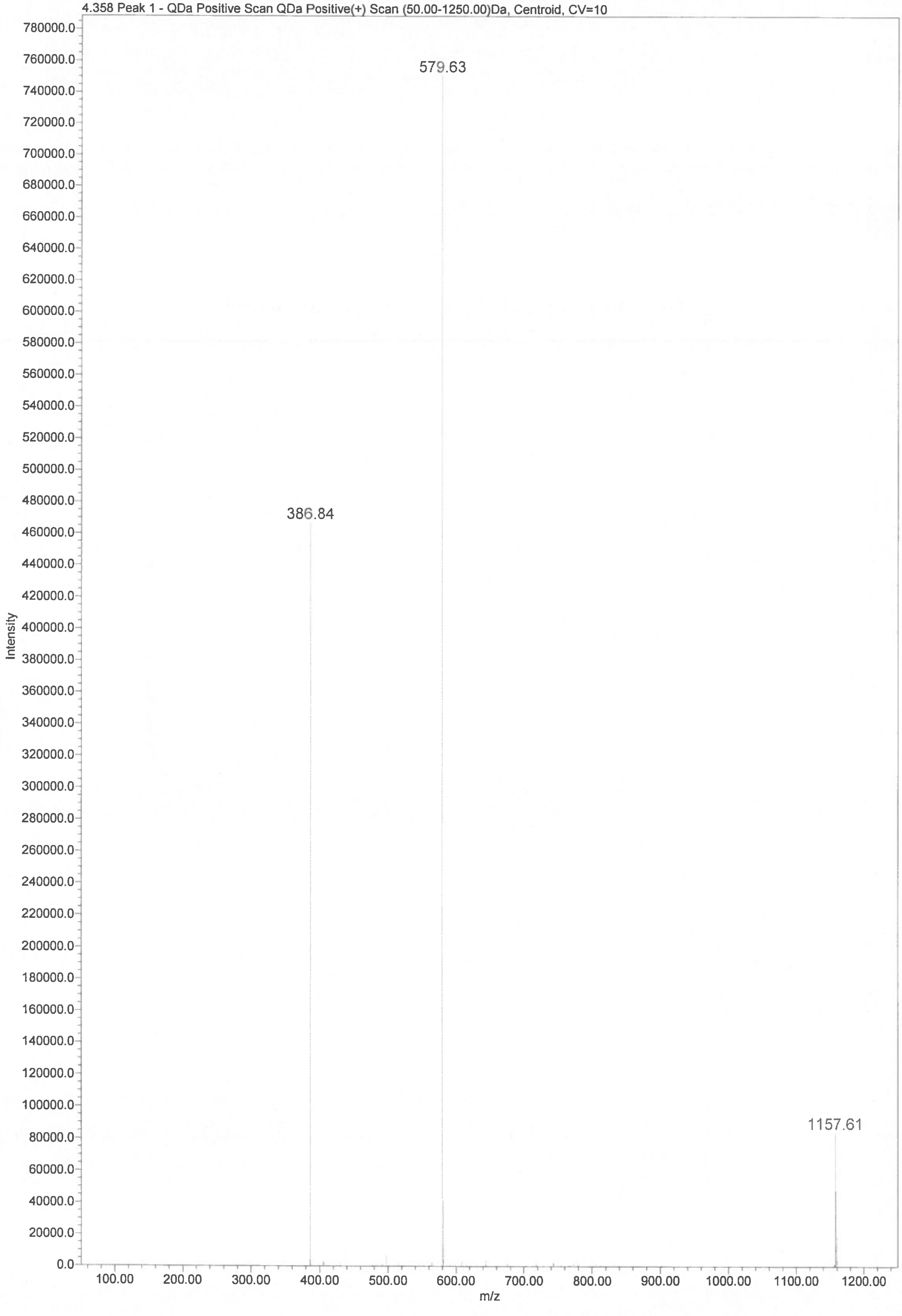
4.358 Peak 1 - QDa Positive Scan QDa Positive(+) Scan (50.00-1250.00)Da, Centroid, CV=10
Intensity
m/z
579.63
386.84
1157.61

Chromatographic analysis

RP-HPLC

Column:	Phenomenex, Luna C18(2), 5 µm, 100 Å, 4,6x150 mm
Mobile phase:	A – water, B – acetonitrile; both containing 0.1% TFA, v/v
Gradient:	10% -100%, 10 minutes
Flow:	2 ml/min
Detection:	UV, 214 nm

Chromatogram : YEWRFYHG, 10-100%, 2ml, 214nm, 10 min57_channel1

System : HPLC
Method : 10-100%, 2ml, 214nm, 10 min
User : User1

Acquired : 2020-01-02 14:41:31
Processed : 2020-01-19 16:03:19
Printed : 2020-01-19 16:03:25

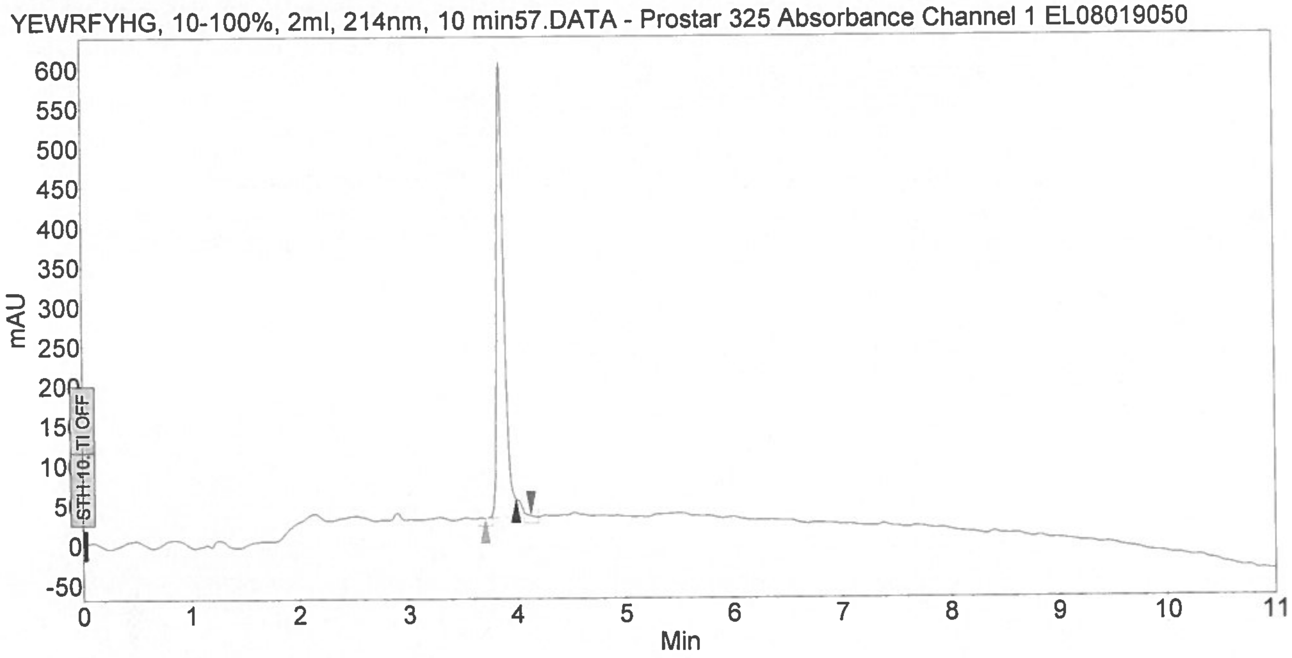

Peak results :

Index	Name	Time [Min]	Quantity [% Area]	Height [mAU]	Area [mAU.Min]	Area % [%]
1	UNKNOWN	3.86	97.18	573.7	43.3	97.185
2	UNKNOWN	3.99	2.82	21.8	1.3	2.815
Total			100.00	595.4	44.6	100.000

PEPTIDEWEB.COM
Phone: +48 783 522 366
E-mail: info@peptideweb.com
Website: www.peptideweb.com
Bysewska 26b
80-298 Gdansk
Poland

21-01-2020

Sequence: EFHKDWG - AP8iii

Average mass: 917.977 Da
Monoisotopic mass: 917.403 Da

Positive scan
m/z: 100-1250

z	Calculated m/z	Measured m/z
1	918.41	918.44
2	459.71	459.91
3	306.81	307.03

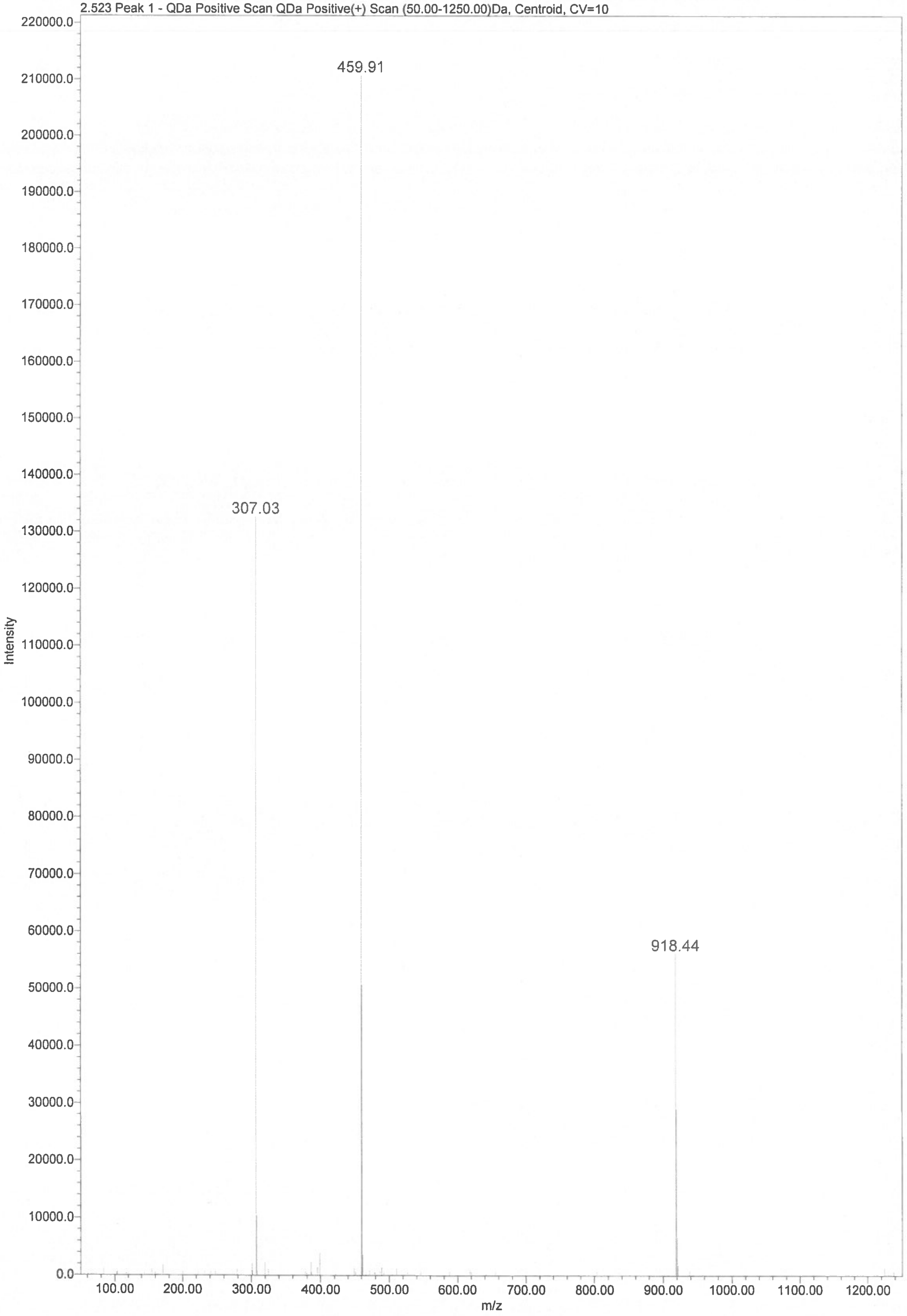
2.523 Peak 1 - QDa Positive Scan QDa Positive(+) Scan (50.00-1250.00)Da, Centroid, CV=10
459.91
307.03
918.44
Intensity
m/z

Phone: +48 783 522 366
E-mail: info@peptideweb.com
Website: www.peptideweb.com

Bysewska 26b
80-298 Gdansk
Poland

Chromatographic analysis

RP-HPLC

Column:	Phenomenex, Luna C18(2), 5 µm, 100 Å, 4,6x150 mm
Mobile phase:	A – water, B – acetonitrile; both containing 0.1% TFA, v/v
Gradient:	10% -100%, 10 minutes
Flow:	2 ml/min
Detection:	UV, 214 nm

Chromatogram : EFHKDWG, 10-100%, 2ml, 214nm, 10 min57_channel1

System : HPLC
Method : 10-100%, 2ml, 214nm, 10 min
User : User1

Acquired : 2019-12-16 13:05:36
Processed : 2020-01-19 16:02:37
Printed : 2020-01-19 16:02:42

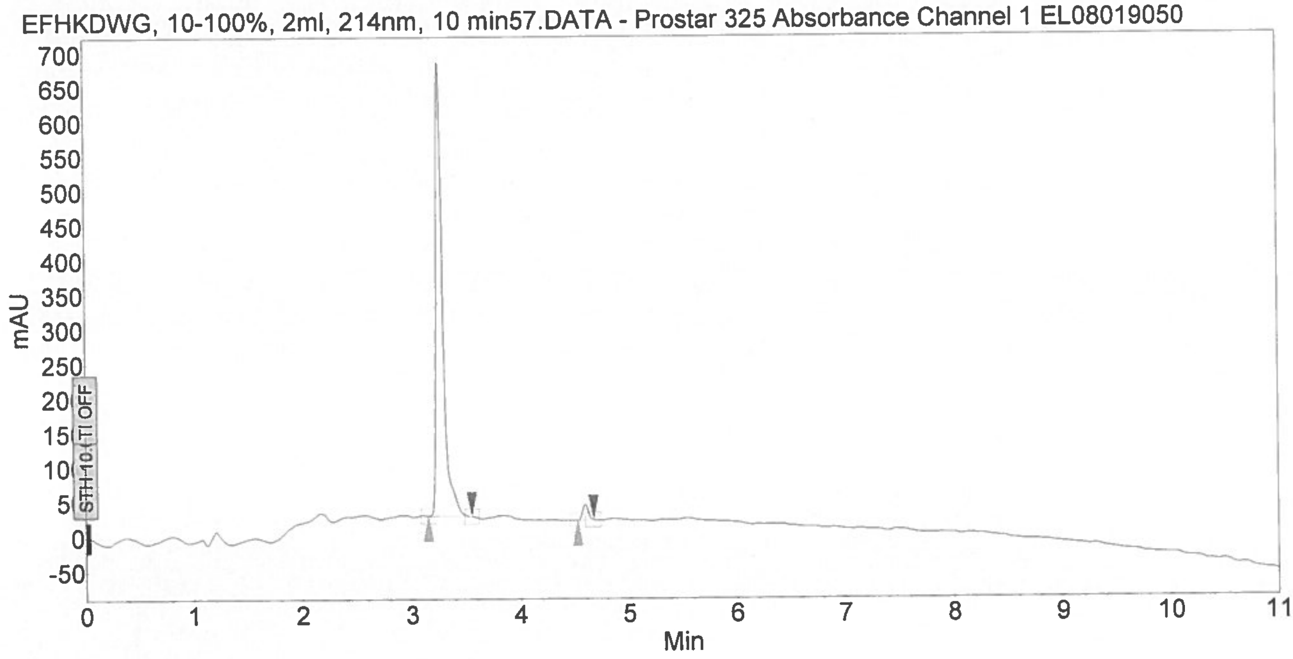

Peak results :

Index	Name	Time [Min]	Quantity [% Area]	Height [mAU]	Area [mAU.Min]	Area % [%]
1	UNKNOWN	3.26	97.19	656.6	47.9	97.193
2	UNKNOWN	4.59	2.81	22.3	1.4	2.807
Total			100.00	678.9	49.3	100.000

PEPTIDEWEB.COM
Phone: +48 783 522 366
E-mail: info@peptideweb.com
Website: www.peptideweb.com
Bysewska 26b
80-298 Gdansk
Poland

21-01-2020

Sequence: EWRFKG – AP8iv

Average mass: 821.934 Da
Monoisotopic mass: 821.418 Da

Positive scan
m/z: 100-1250

z	Calculated m/z	Measured m/z
1	822.43	822.49
2	411.72	411.95
3	274.81	274.98

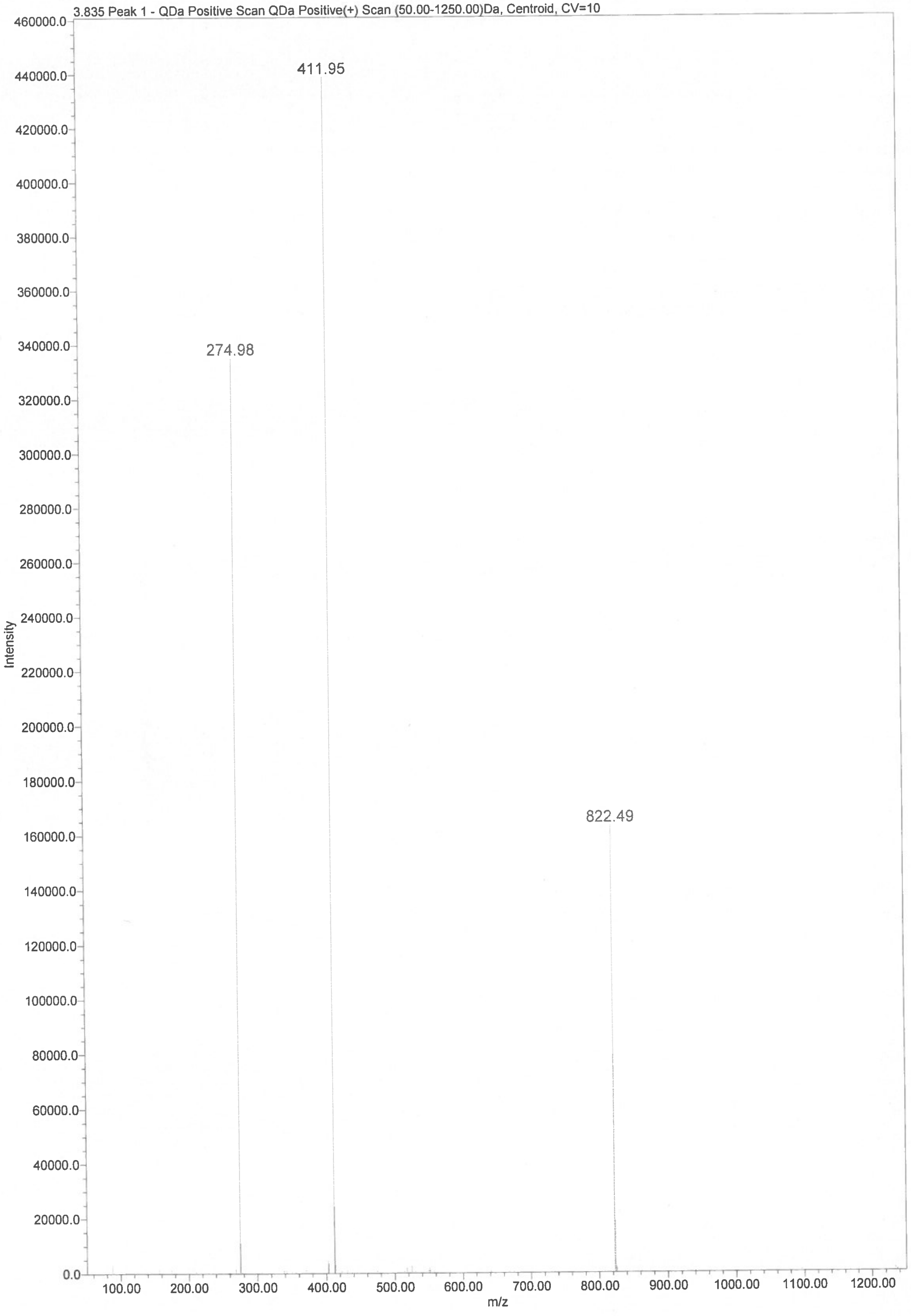

3.835 Peak 1 - QDa Positive Scan QDa Positive(+) Scan (50.00-1250.00)Da, Centroid, CV=10
411.95
274.98
822.49
Intensity
m/z
460000.0
440000.0
420000.0
400000.0
380000.0
360000.0
340000.0
320000.0
300000.0
280000.0
260000.0
240000.0
220000.0
200000.0
180000.0
160000.0
140000.0
120000.0
100000.0
80000.0
60000.0
40000.0
20000.0
0.0
100.00
200.00
300.00
400.00
500.00
600.00
700.00
800.00
900.00
1000.00
1100.00
1200.00

Chromatographic analysis

RP-HPLC

Column:	Phenomenex, Luna C18(2), 5 µm, 100 Å, 4,6x150 mm
Mobile phase:	A – water, B – acetonitrile; both containing 0.1% TFA, v/v
Gradient:	10% -100%, 10 minutes
Flow:	2 ml/min
Detection:	UV, 214 nm

Chromatogram : EWRFKG, 10-100%, 2ml, 214nm, 10 min58_channel1

System : HPLC
Method : 10-100%, 2ml, 214nm, 10 min
User : User1

Acquired : 2020-01-03 17:16:03
Processed : 2020-01-19 14:58:27
Printed : 2020-01-19 14:58:34

EWRFKG, 10-100%, 2ml, 214nm, 10 min58.DATA - Prostar 325 Absorbance Channel 1 EL08019050

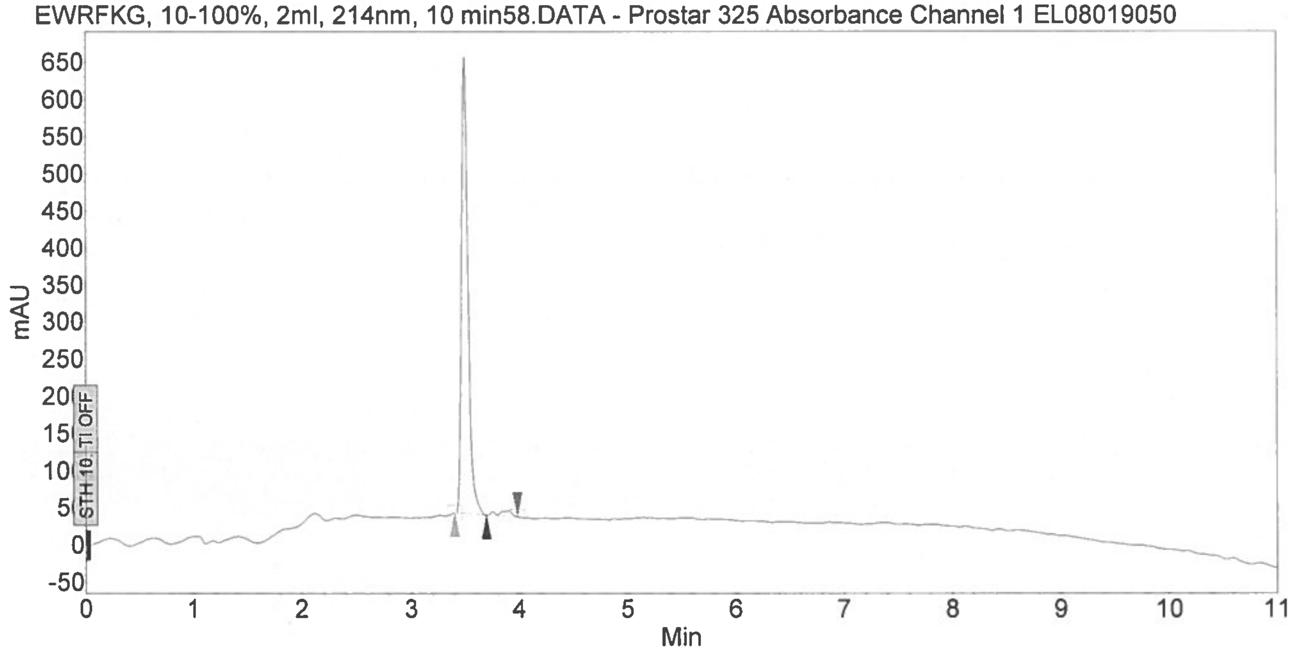

Peak results :

Index	Name	Time [Min]	Quantity [% Area]	Height [mAU]	Area [mAU.Min]	Area % [%]
1	UNKNOWN	3.50	97.79	614.1	43.1	97.794
2	UNKNOWN	3.90	2.21	7.3	1.0	2.206
Total			100.00	621.5	44.1	100.000

PEPTIDEWEB.COM
Phone: +48 783 522 366
E-mail: info@peptideweb.com
Website: www.peptideweb.com
Bysewska 26b
80-298 Gdansk
Poland

21-01-2020

Sequence: RhB-LDWRHQFIG   RhB-AP8i

Average mass: 1596.849 Da
Monoisotopic mass: 1595.816 Da

Positive scan
m/z: 100-1250

z	Calculated m/z	Measured m/z
1	1596.82	-
2	798.92	799.35
3	532.95	533.06

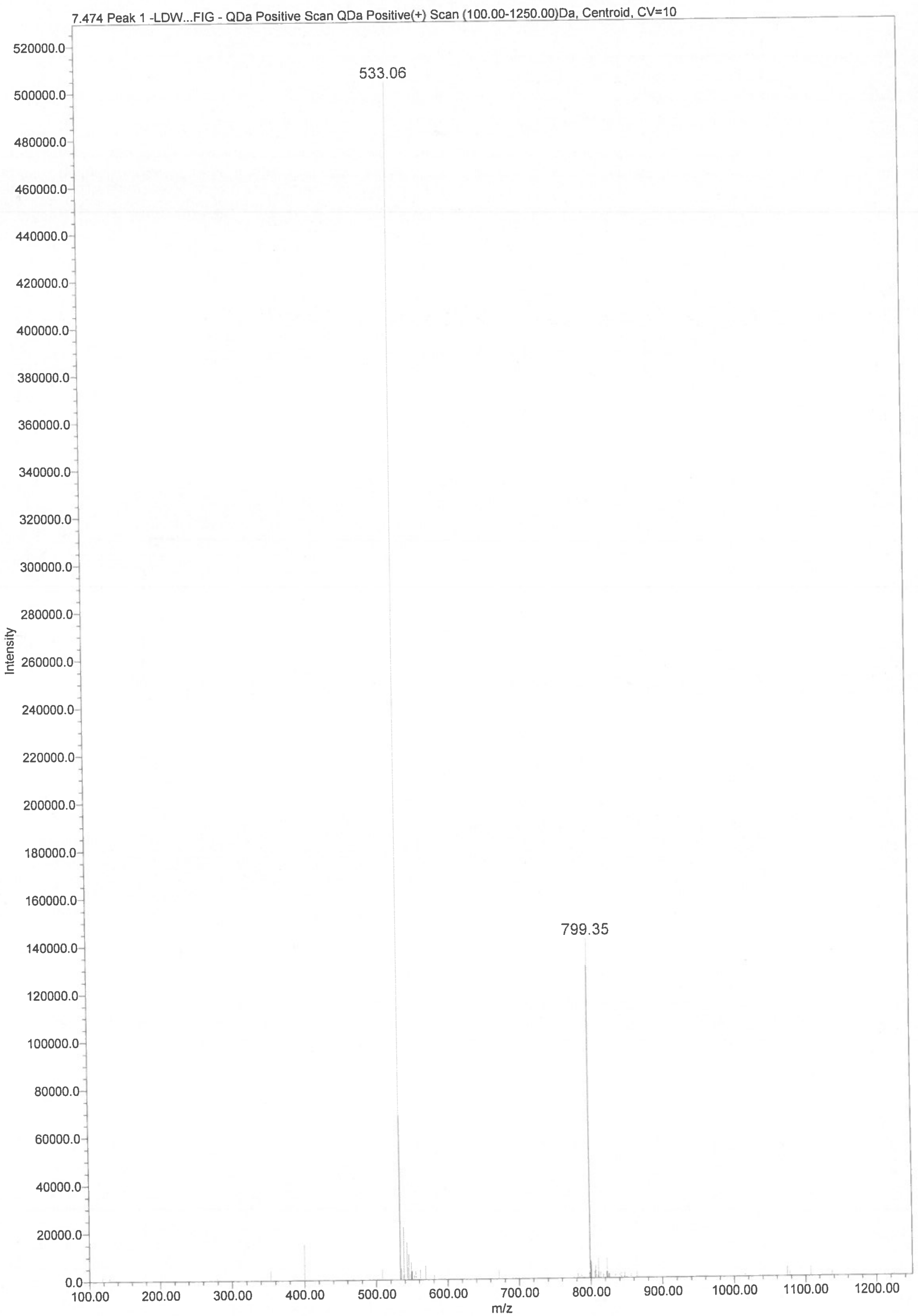
7.474 Peak 1 -LDW...FIG - QDa Positive Scan QDa Positive(+) Scan (100.00-1250.00)Da, Centroid, CV=10
533.06
799.35
Intensity
m/z
520000.0
500000.0
480000.0
460000.0
440000.0
420000.0
400000.0
380000.0
360000.0
340000.0
320000.0
300000.0
280000.0
260000.0
240000.0
220000.0
200000.0
180000.0
160000.0
140000.0
120000.0
100000.0
80000.0
60000.0
40000.0
20000.0
0.0
100.00
200.00
300.00
400.00
500.00
600.00
700.00
800.00
900.00
1000.00
1100.00
1200.00

Phone: +48 783 522 366
E-mail: info@peptideweb.com
Website: www.peptideweb.com

Bysewska 26b
80-298 Gdansk
Poland

Chromatographic analysis

RP-HPLC

Column:	Phenomenex, Luna C18(2), 5 µm, 100 Å, 4,6x150 mm
Mobile phase:	A – water, B – acetonitrile
Gradient:	10% -100%, 10 minutes
Flow:	2 ml/min
Detection:	UV, 214 nm

Chromatogram : RhB-LDW...FIG, 10-100%, 2ml, 214nm, 10 min58_channel1

System : HPLC
Method : 10-100%, 2ml, 214nm, 10 min
User : User1

Acquired : 2020-01-19 15:44:04
Processed : 2020-01-19 16:01:33
Printed : 2020-01-19 16:01:36

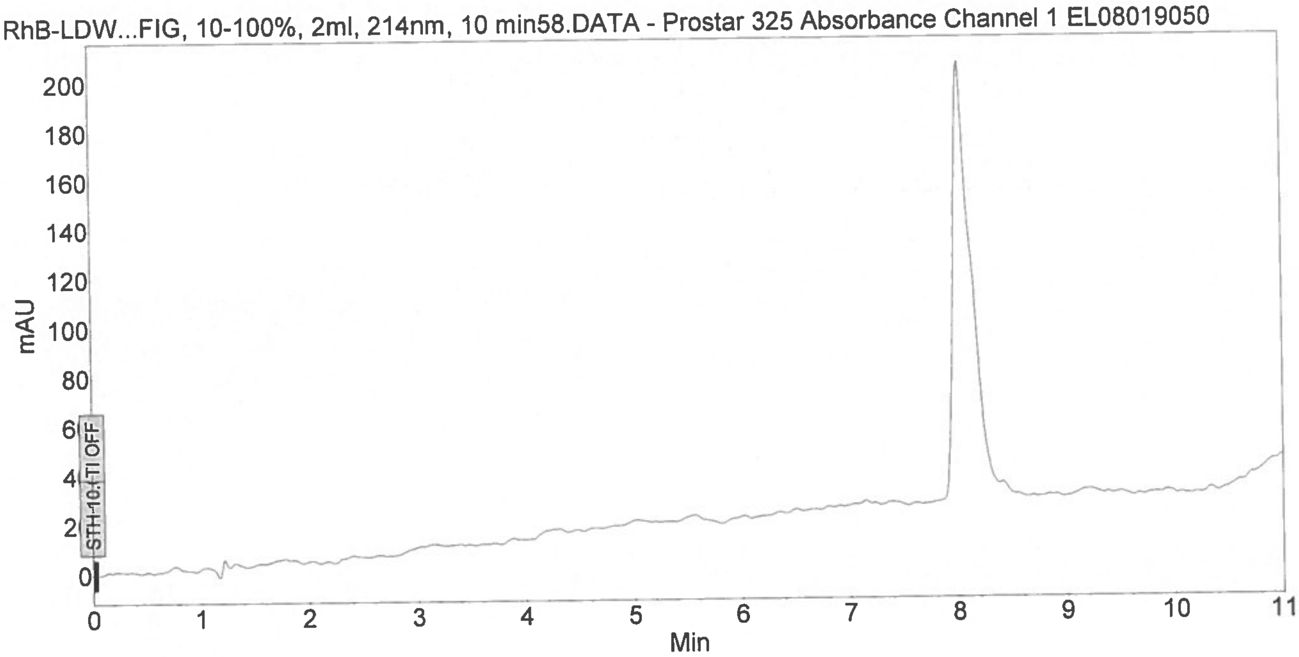

Peak results :

Index	Name	Time [Min]	Quantity [% Area]	Height [mAU]	Area [mAU.Min]	Area % [%]
Total			0.00	0.0	0.0	0.000

PEPTIDEWEB.COM

Phone: +48 783 522 366
E-mail: info@peptideweb.com
Website: www.peptideweb.com

Bysewska 26b
80-298 Gdansk
Poland

21-01-2020

Sequence: RhB-YEWRFYHG RhB-AP8ii

Average mass: 1582.777 Da
Monoisotopic mass: 1581.731 Da

Positive scan
m/z: 100-1250

z	Calculated m/z	Measured m/z
1	1582.74	-
2	791.87	791.73
3	528.25	528.36

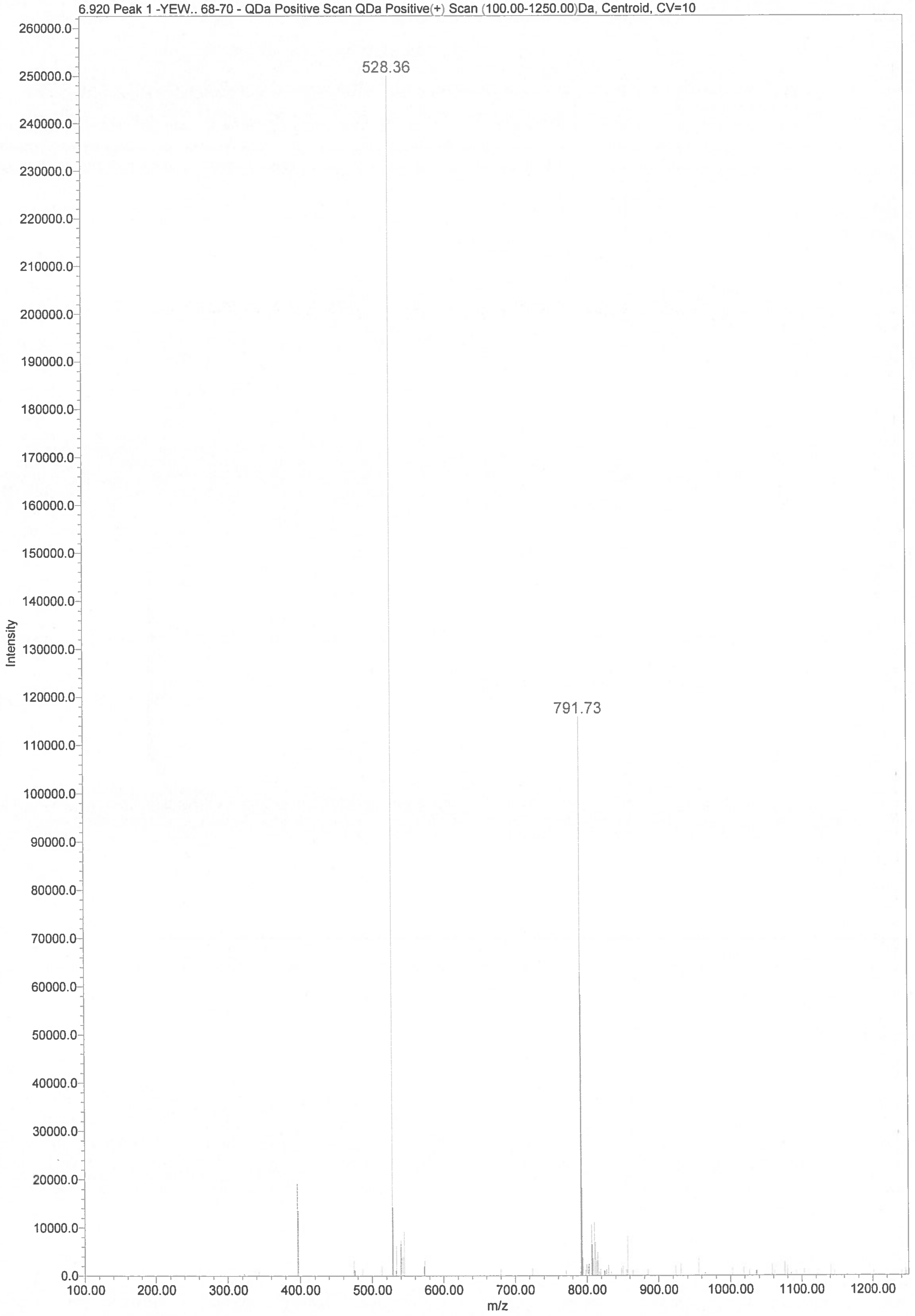
6.920 Peak 1 -YEW.. 68-70 - QDa Positive Scan QDa Positive(+) Scan (100.00-1250.00)Da, Centroid, CV=10
528.36
791.73
Intensity
m/z
260000.0
250000.0
240000.0
230000.0
220000.0
210000.0
200000.0
190000.0
180000.0
170000.0
160000.0
150000.0
140000.0
130000.0
120000.0
110000.0
100000.0
90000.0
80000.0
70000.0
60000.0
50000.0
40000.0
30000.0
20000.0
10000.0
0.0
100.00
200.00
300.00
400.00
500.00
600.00
700.00
800.00
900.00
1000.00
1100.00
1200.00

Chromatographic analysis

RP-HPLC

Column:	Phenomenex, Luna C18(2), 5 µm, 100 Å, 4,6x150 mm
Mobile phase:	A – water, B – acetonitrile
Gradient:	10% -100%, 10 minutes
Flow:	2 ml/min
Detection:	UV, 214 nm

Chromatogram : RhB-YEW..YHG, 10-100%, 2ml, 214nm, 10 min58_channel1

System : HPLC
Method : 10-100%, 2ml, 214nm, 10 min
User : User1

Acquired : 2020-01-19 15:27:07
Processed : 2020-01-19 15:42:23
Printed : 2020-01-19 15:42:29

RhB-YEW..YHG, 10-100%, 2ml, 214nm, 10 min58.DATA - Prostar 325 Absorbance Channel 1 EL08019050

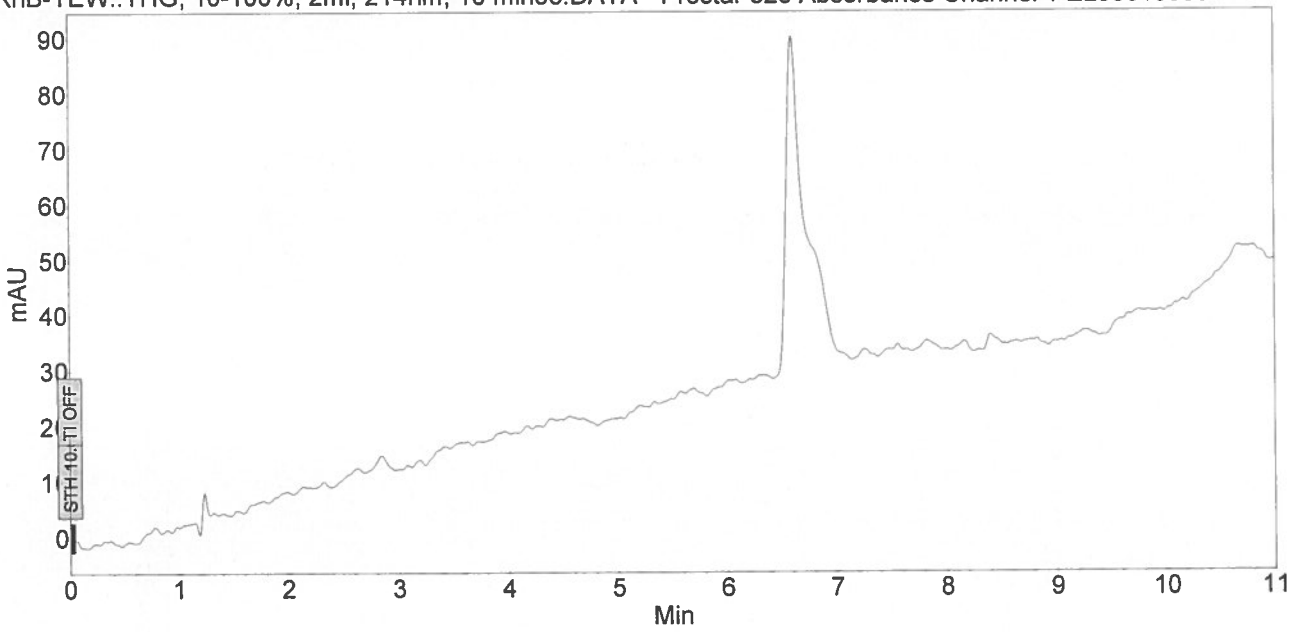

Peak results :

Index	Name	Time [Min]	Quantity [% Area]	Height [mAU]	Area [mAU.Min]	Area % [%]
Total			0.00	0.0	0.0	0.000

PEPTIDEWEB.COM
Phone: +48 783 522 366
E-mail: info@peptideweb.com
Website: www.peptideweb.com
Bysewska 26b
80-298 Gdansk
Poland

21-01-2020

Sequence: RhB-EFHKDWG RhB- AP8iii

Average mass: 1343.505 Da
Monoisotopic mass: 1342.626 Da

Positive scan
m/z: 100-1250

z	Calculated m/z	Measured m/z
1	1343.63	-
2	672.32	672.30
3	448.55	448.62
4	336.66	336.93

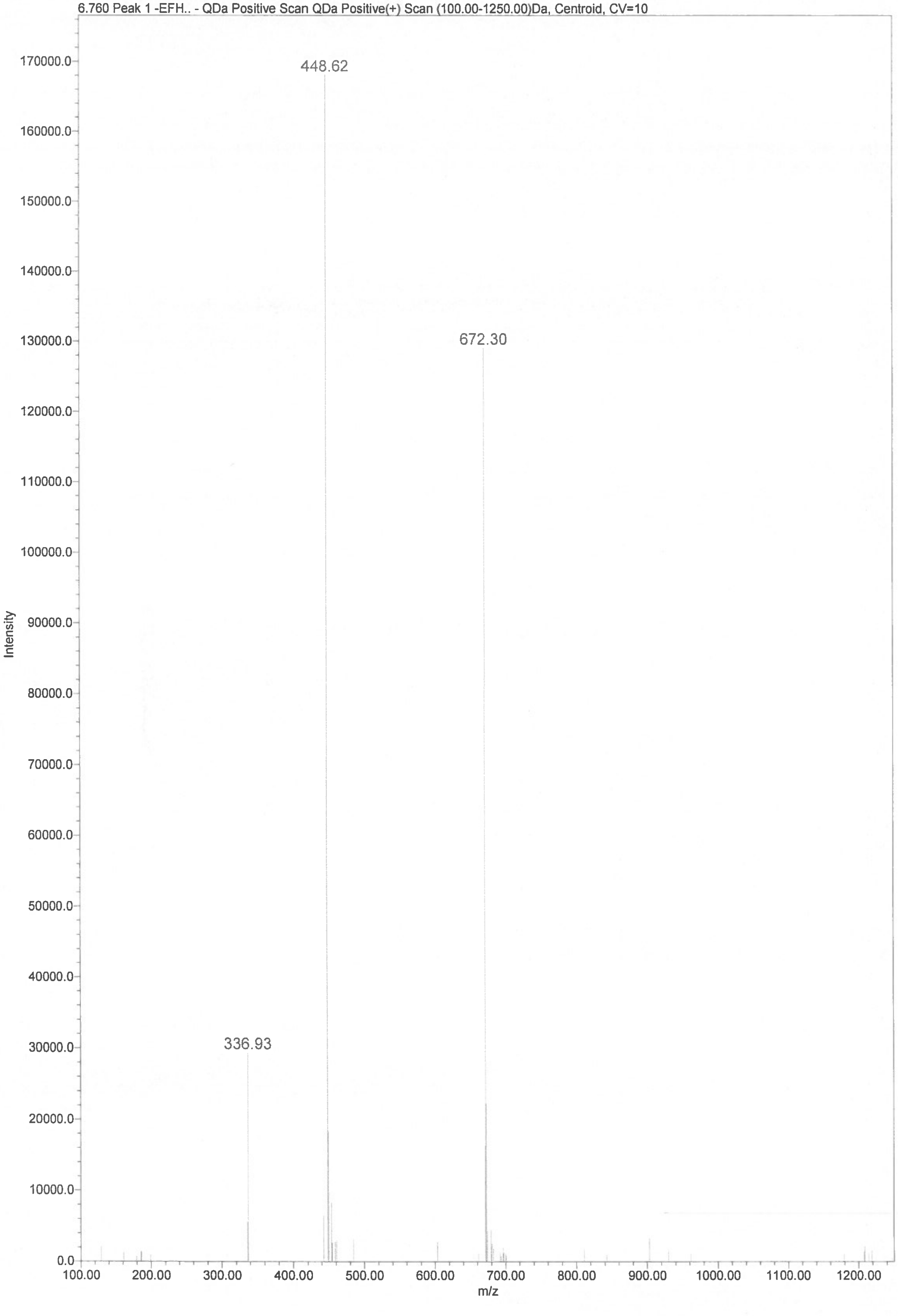
6.760 Peak 1 -EFH.. - QDa Positive Scan QDa Positive(+) Scan (100.00-1250.00)Da, Centroid, CV=10
448.62
672.30
336.93
Intensity
m/z
0.0
10000.0
20000.0
30000.0
40000.0
50000.0
60000.0
70000.0
80000.0
90000.0
100000.0
110000.0
120000.0
130000.0
140000.0
150000.0
160000.0
170000.0
100.00
200.00
300.00
400.00
500.00
600.00
700.00
800.00
900.00
1000.00
1100.00
1200.00

PEPTIDEWEB.COM
Phone: +48 783 522 366
E-mail: info@peptideweb.com
Website: www.peptideweb.com
Bysewska 26b
80-298 Gdansk
Poland

Chromatographic analysis

RP-HPLC

Column:	Phenomenex, Luna C18(2), 5 µm, 100 Å, 4,6x150 mm
Mobile phase:	A – water, B – acetonitrile
Gradient:	10% -100%, 10 minutes
Flow:	2 ml/min
Detection:	UV, 214 nm

Chromatogram : RhB-EFHKDWG, 10-100%, 2ml, 214nm, 10 min58_channel1

System : HPLC
Method : 10-100%, 2ml, 214nm, 10 min
User : User1

Acquired : 2020-01-19 15:12:36
Processed : 2020-01-19 15:41:50
Printed : 2020-01-19 15:42:06

RhB-EFHKDWG, 10-100%, 2ml, 214nm, 10 min58.DATA - Prostar 325 Absorbance Channel 1 EL08019050

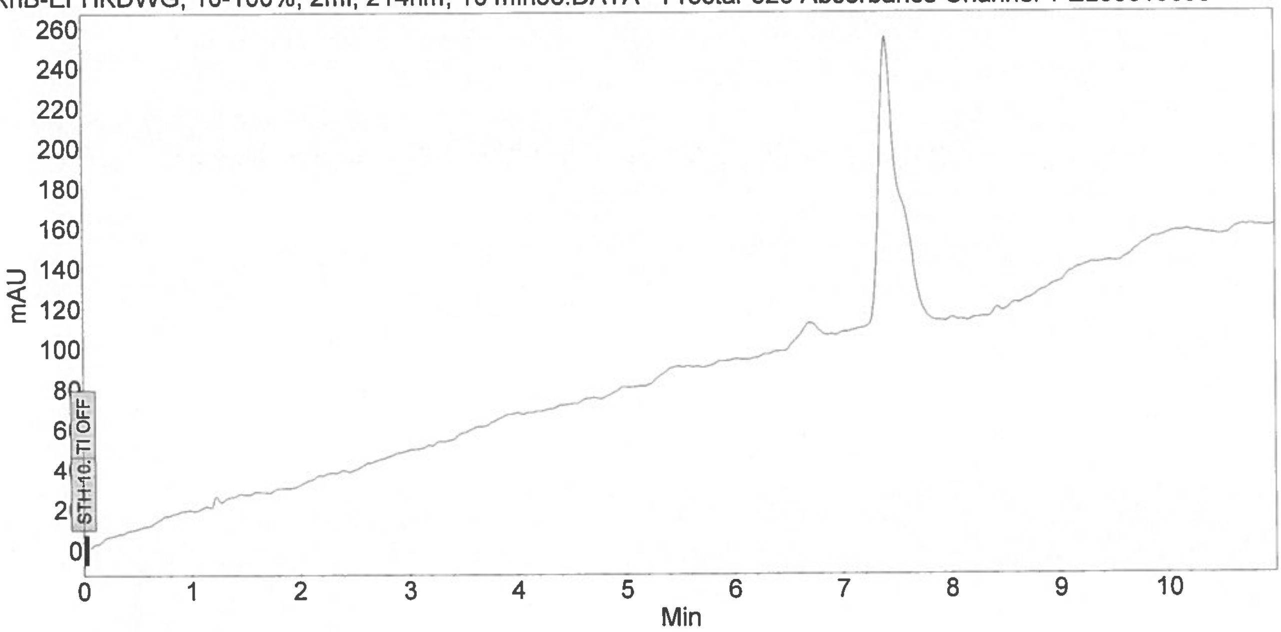

Peak results :

Index	Name	Time [Min]	Quantity [% Area]	Height [mAU]	Area [mAU.Min]	Area % [%]
Total			0.00	0.0	0.0	0.000

PEPTIDEWEB.COM
Phone: +48 783 522 366
E-mail: info@peptideweb.com
Website: www.peptideweb.com
Bysewska 26b
80-298 Gdansk
Poland

21-01-2020

Sequence: RhB-EWRFKG - RhB-AP8iv

Average mass: 1247.462 Da
Monoisotopic mass: 1246.641 Da

Positive scan
m/z: 100-1250

z	Calculated m/z	Measured m/z
1	1247.65	1246.85
2	624.33	624.31
3	416.55	416.63

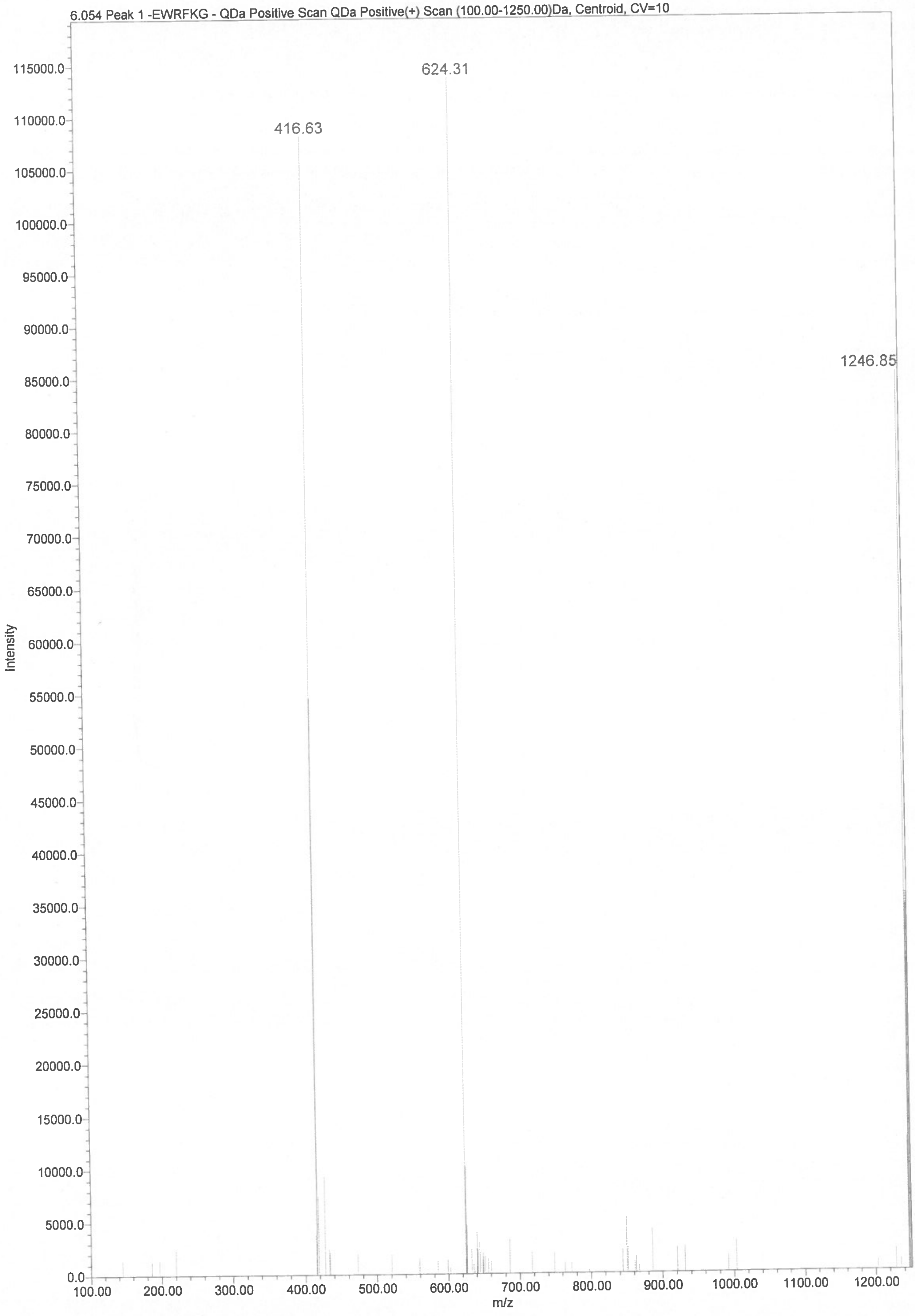
6.054 Peak 1 -EWRFKG - QDa Positive Scan QDa Positive(+) Scan (100.00-1250.00)Da, Centroid, CV=10
624.31
416.63
1246.85
Intensity
m/z
115000.0
110000.0
105000.0
100000.0
95000.0
90000.0
85000.0
80000.0
75000.0
70000.0
65000.0
60000.0
55000.0
50000.0
45000.0
40000.0
35000.0
30000.0
25000.0
20000.0
15000.0
10000.0
5000.0
0.0
100.00
200.00
300.00
400.00
500.00
600.00
700.00
800.00
900.00
1000.00
1100.00
1200.00

Chromatographic analysis

RP-HPLC

Column:	Phenomenex, Luna C18(2), 5 µm, 100 Å, 4,6x150 mm
Mobile phase:	A – water, B – acetonitrile
Gradient:	10% -100%, 10 minutes
Flow:	2 ml/min
Detection:	UV, 214 nm

Chromatogram : RhB-EWRFKG, 10-100%, 2ml, 214nm, 10 min58_channel1

System : HPLC
Method : 10-100%, 2ml, 214nm, 10 min
User : User1

Acquired : 2020-01-19 16:00:55
Processed : 2020-01-19 16:12:52
Printed : 2020-01-19 18:13:33

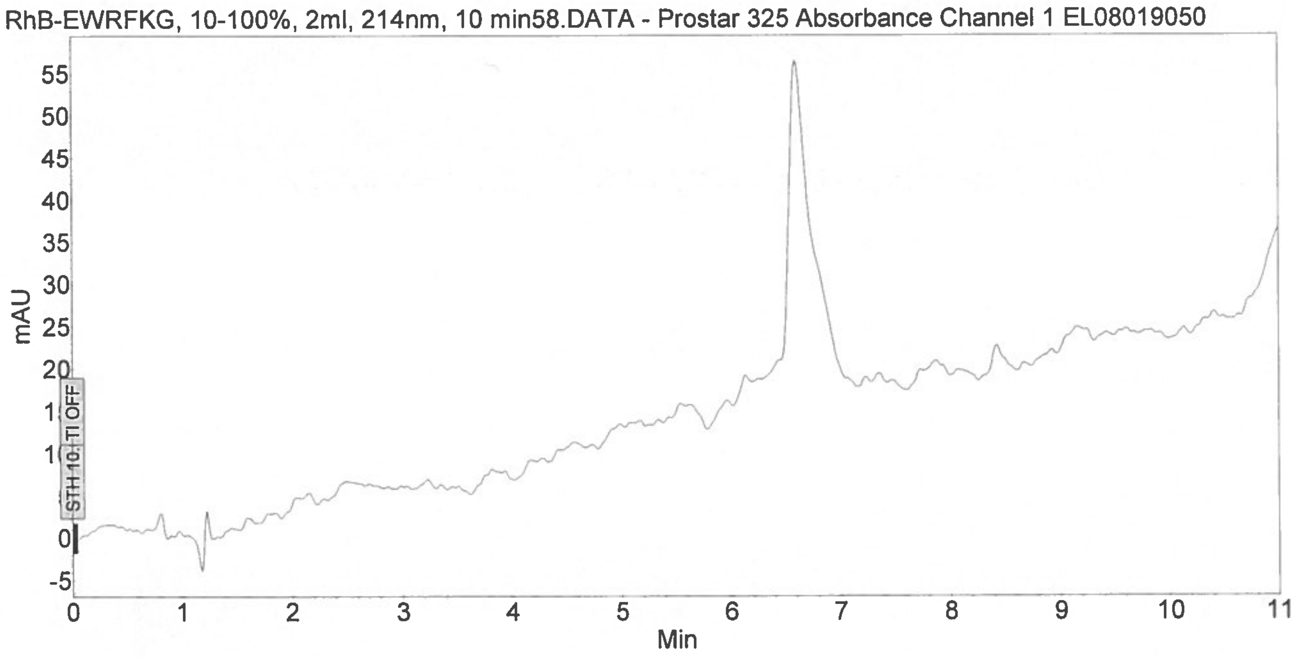

Peak results :

Index	Name	Time [Min]	Quantity [% Area]	Height [mAU]	Area [mAU.Min]	Area % [%]
Total			0.00	0.0	0.0	0.000

18-04-2024

Sequence: Biotin-YEWRFYHG
Molecular mass: 1385.545 Da
Exact mass: 1384.819 Da

Range *m/z*: 50-1250

z	Positive ionization	
	Calculated m/z	Measured m/z
1	**1385.82**	-
2	**693.41**	692.77
3	**462.61**	-

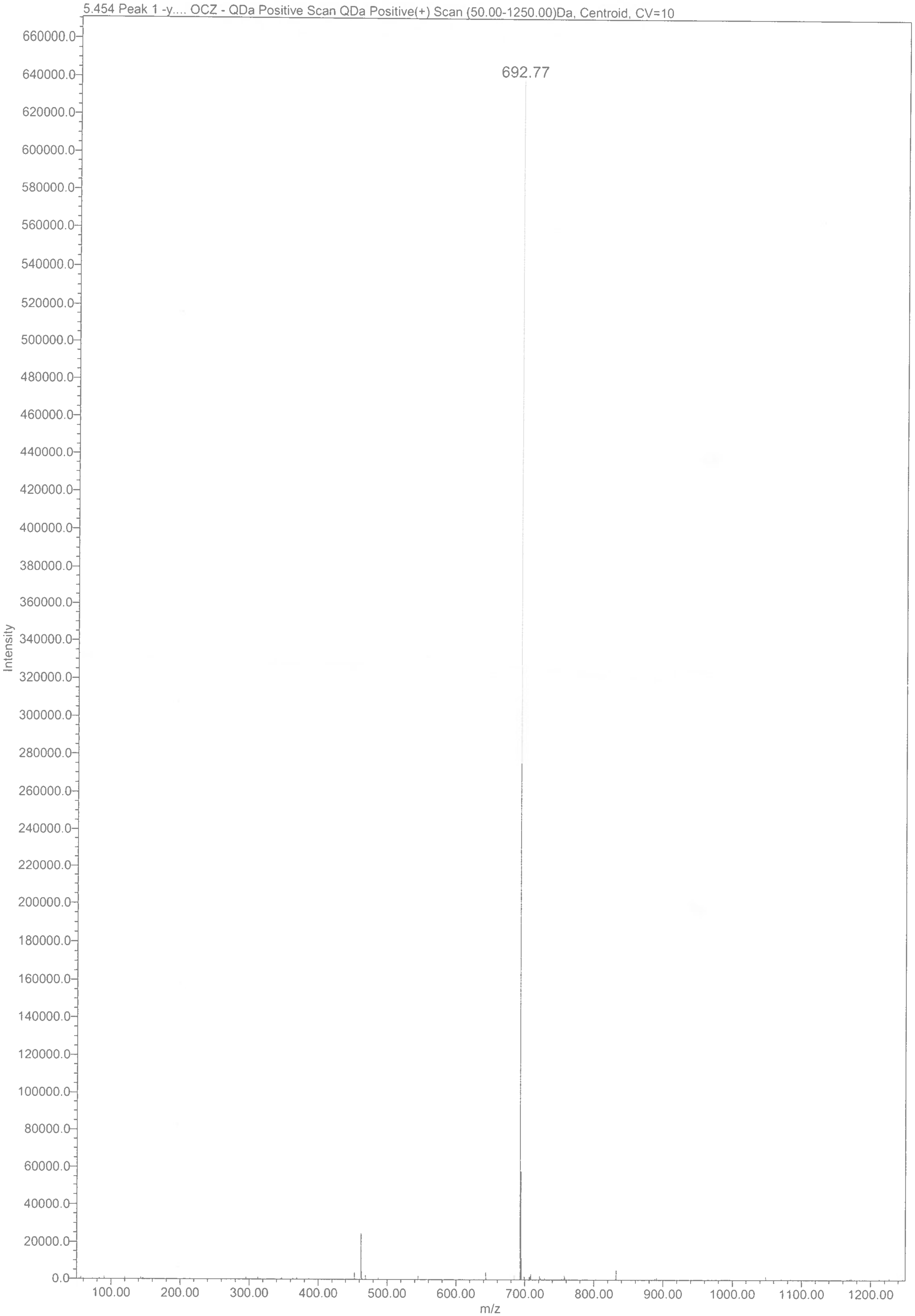
5.454 Peak 1 -y.... OCZ - QDa Positive Scan QDa Positive(+) Scan (50.00-1250.00)Da, Centroid, CV=10
692.77
Intensity
m/z
0.0
20000.0
40000.0
60000.0
80000.0
100000.0
120000.0
140000.0
160000.0
180000.0
200000.0
220000.0
240000.0
260000.0
280000.0
300000.0
320000.0
340000.0
360000.0
380000.0
400000.0
420000.0
440000.0
460000.0
480000.0
500000.0
520000.0
540000.0
560000.0
580000.0
600000.0
620000.0
640000.0
660000.0
100.00
200.00
300.00
400.00
500.00
600.00
700.00
800.00
900.00
1000.00
1100.00
1200.00

Phone: +48 783 522 366
E-mail: info@peptideweb.com
Website: www.peptideweb.com

Bysewska 26b
80-298 Gdansk
Poland

Chromatographic analysis

RP-HPLC

Column: Nucleosil 120, C18, 5 µm, 4.0x125 mm

Mobile phase: A – water+ 0.1% TFA, v/v, B – acetonitrile + 0.1% TFA, v/v;

Gradient: 10-90%, 10 min

Flow: 2 ml/min

UV detection: UV, 214 nm

Chromatogram : Biot-Y.......10-90%, 10min, 214nm, 2ml4_channel1

System : HPLC
Method : 10-90%, 10min, 214nm, 2ml
User : User1

Acquired : 2024-04-18 10:24:20
Processed : 2024-04-18 11:41:30
Printed : 2024-04-18 11:41:40

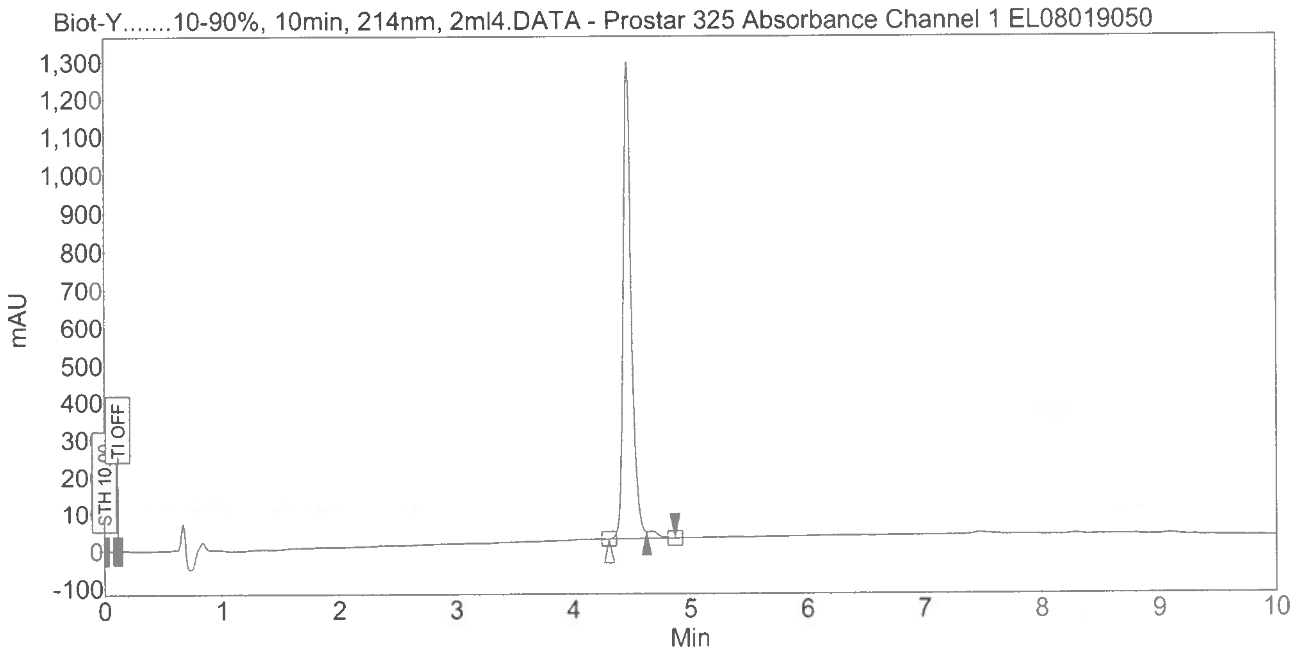

Peak results :

Index	Name	Time [Min]	Quantity [% Area]	Height [mAU]	Area [mAU.Min]	Area % [%]
1	UNKNOWN	4.47	98.15	1267.5	102.7	98.150
2	UNKNOWN	4.67	1.85	18.5	1.9	1.850
Total			100.00	1285.9	104.7	100.000
